# Dual-Phase Multi-Stimuli-Responsive Luminescence from a Pentiptycene-Linked Binuclear Cyclometalated Platinum(II) Complex

**DOI:** 10.1021/acs.inorgchem.5c02560

**Published:** 2025-07-31

**Authors:** Ying-Feng Hsu, Yu-Chieh Ho, Yi-Hung Liu, Jye-Shane Yang

**Affiliations:** † Department of Chemistry, 33561 National Taiwan University, Taipei 10617, Taiwan; ‡ Center for Emerging Material and Advanced Devices, National Taiwan University, Taipei 10617, Taiwan

## Abstract

While numerous stimuli-responsive molecular systems have been reported, materials that respond to external stimuli in both solution and solid phases remain rare. Herein, we report a bis­(phenylsulfonyl)­pentiptycene (BPSP)-linked binuclear N^∧^C^∧^N cyclometalated Pt­(II) acetylide complex that exhibits dual-phase luminochromic responses (DPLR) to multiple stimuli. These include temperature changes and the presence of Ag­(I), Cu­(I), dimethyl sulfoxide (DMSO), or O_2_ in tetrahydrofuran (THF) solution, as well as mechanical grinding and benzene vapor in the solid state. The luminochromic behaviors arise from switching between green monomer and red excimer emission of the NCNPt-acetylide chromophores. In solution, excimer emission dominates due to the folded conformation of the BPSP linker but can be suppressed by external stimuli, resulting in red-to-green switching. In the solid state, the BPSP linker adopts an extended conformation that promotes intermolecular π–π interactions. The initial green monomer emission shifts to red emission upon mechanical grinding (green-to-red switching), and partial recovery of monomer emission, manifested as red-to-orange switching, can be achieved by fuming the ground samples with benzene or ethyl acetate vapors. This work highlights the utility of a conformationally flexible linker for designing binuclear Pt­(II) complexes capable of luminochromic behavior across both solution and solid phases.

## Graphical abstract

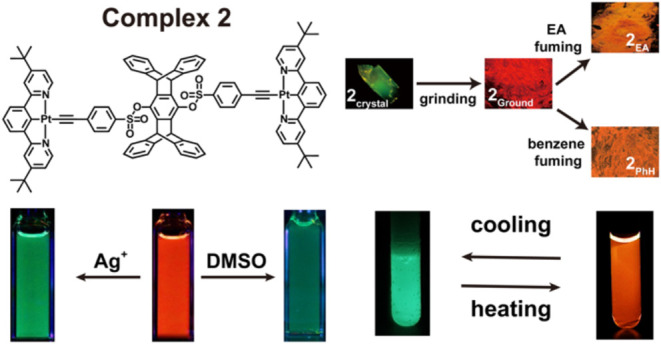

## Introduction

Stimuli-responsive luminescent materials that exhibit changes in emission color in response to external stimuli such as mechanical stress, light, heat, or chemical agents have attracted significant attention due to their potential applications in sensing, anticounterfeiting, and bioimaging.^1–8^ For molecular design, square-planar Pt­(II) complexes are particularly attractive because their photoluminescence can be tuned between monomer and excimer emission, as well as between metal-to-ligand charge transfer (MLCT) and metal–metal-to-ligand charge transfer (MMLCT) emission, by controlling π–π and Pt­(II)–Pt­(II) interactions.^9,10^ For solution-state applications, molecular designs often incorporate flexible linkers connecting two Pt­(II) centers (i.e., binuclear Pt­(II) complexes), enabling modulation of intramolecular π–π and/or Pt­(II)–Pt­(II) interactions through stimulus-induced conformational changes.^11–14^ In the solid state, both intramolecular and intermolecular π–π and Pt­(II)–Pt­(II) interactions can contribute to luminochromic behavior.^15–22^ However, despite numerous reports of stimuli-responsive Pt­(II) complexes, examples that exhibit luminochromic responses in both solution and solid phases (i.e., dual-phase luminochromic responses, DPLR) remain scarce.^23^

We recently demonstrated that attaching a bulky H-shaped pentiptycene scaffold to an organic^24–27^ or organometallic^28–30^ luminophore via an acetylene or isocyanate linker enables solid-state luminescence behaviors such as mechanochromism, vapochromism, thermochromism, photochromism, and/or photomechanochromism. In Pt­(II) systems, the *tert*-butyl-substituted N^∧^C^∧^N ligand, as found in complex **1** (Figure 1), provides a “just right” steric profile that facilitates stimulus-induced switching between the monomer (green) and excimer (red) emission, resulting in high-contrast luminochromic responses.^29^ In this paper, we report a binuclear Pt­(II) complex (**2**) featuring a bis­(phenylsulfonyl)­pentiptycene (BPSP) linker that exhibits DPLR. In solution, the relative intensity of green monomer and red excimer emission in complex **2** can be modulated by external stimuli such as temperature, Ag­(I), O_2_, and dimethyl sulfoxide (DMSO). In the solid state, its luminescence color switches among green, orange, and red in response to mechanical grinding and solvent vapor. To elucidate the structural features responsible for these behaviors, a mononuclear analogue **3** was also investigated for comparison. This work demonstrates that binuclear Pt­(II) complexes with flexible linkers are promising candidates for exhibiting DPLR in response to multiple external stimuli.

**Figure 1:**
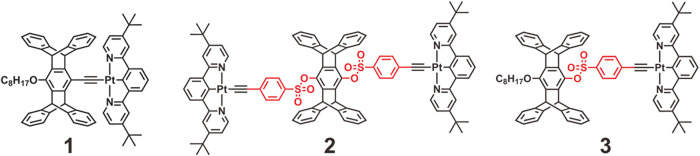
Chemical structures of N^∧^C^∧^N-cyclometalated Pt­(II) complexes **1**–**3**, with the phenylsulfonate moiety highlighted.

## Results and Discussion

### Molecular Design and Synthesis

To enable DPLR in a binuclear Pt­(II) complex, the linker must support tunable intra- and/or intermolecular interactions in both solution and solid states. In solution, a foldable linker is needed to bring the two Pt centers into close proximity, facilitating interactions that give rise to excimer or MMLCT emission.^11–14^ Luminochromic responses emerge from the modulation, either weakening or strengthening, of these interactions via intramolecular monomer–excimer or MLCT-MMLCT mechanisms. In the solid state, where molecular motion is restricted by the rigid matrix, the linker’s conformation plays a critical role. If it remains folded, a finely tuned balance of intramolecular and intermolecular interactions is required for emission switching.^14^ Alternatively, if the linker adopts an extended conformation, intermolecular interactions can dominate, allowing monomer–excimer or MLCT-MMLCT transitions to drive luminochromic behavior.^15^

The molecular design of **2** incorporates both the high-contrast monomer–excimer emission properties of the NCNPt-acetylide chromophore, as demonstrated by **1**,^29^ and the foldable-and-extendable dual function of the BPSP linker, inspired by its bis­(phenylsulfonate)­triptycene counterpart.^31^ We envisioned that replacing the triptycene unit with pentiptycene could enhance the linker’s folding ability, as folding is facilitated not only by the sulfonate groups but also by C–H···π interactions between the triptycene unit and the phenyl rings. With one additional iptycenyl substituent in pentiptycene compared to triptycene, the conformational freedom of the sulfonate groups is likely reduced. Consequently, the probability of intramolecular excimer formation from the NCNPt cores is increased, paving the way for luminochromic performance in solution. On the other hand, example of an extended conformation for the BPSP linker in the solid state is known,^32^ which holds promise for **2** to perform solid-state luminochromic responses via intermolecular monomer–excimer mechanisms.

The synthesis of Pt­(II) complex **2** starting from pentiptycene hydroquinone is outlined in Scheme 1. The reaction between pentiptycene hydroquinone and 4-bromobenzenesulfonyl chloride in the presence of potassium carbonate in acetone afforded compound **4**. The subsequent Sonogashira coupling with trimethylsilylacetylene provided compound **5**. The silyl group was then removed by potassium carbonate to obtain compound **6**, followed by the reaction with the known NCNPt­(II) complex **7** ^29^ in the presence of CuI and di­(isopropyl)­amine to produce the target complex **2**. For the preparation of complex **3** (Scheme S1), the first step involves octylation prior to sulfonylation with bromobenzenesulfonyl chloride. The subsequent synthetic steps follow a protocol similar to that used for complex **2**, as described above. Detailed synthetic procedures and characterization data are provided in the Experimental Section and Supporting Information.

**Scheme 1:**
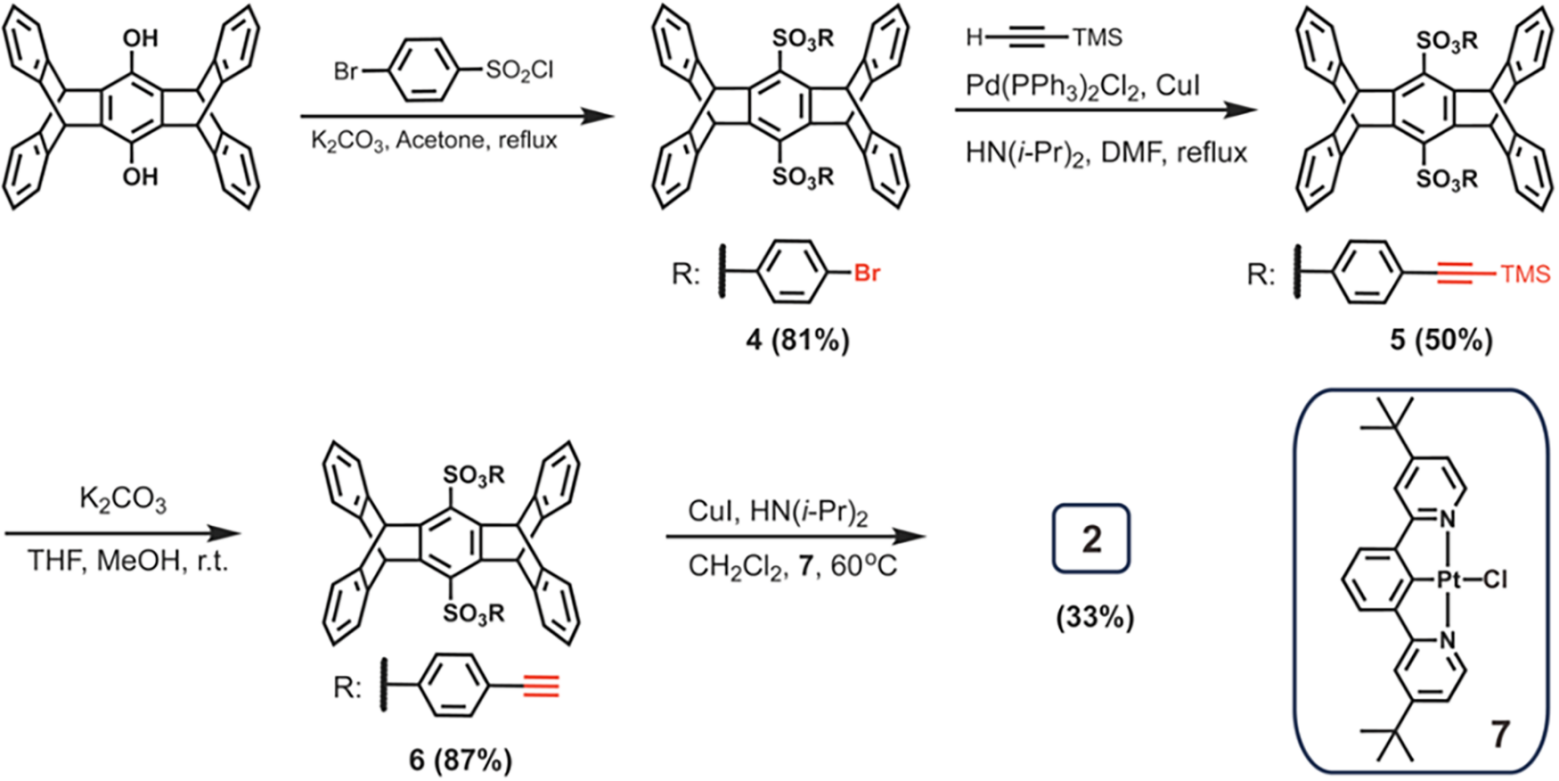
Synthesis of **2**

### Molecular and Crystal Structure

The molecular structure of **2** in the gas phase has been investigated through DFT calculations (M06-2X//SDD for Au, 6-31G­(d,p) for other atoms).^33–35^ The BPSP linker can be extended or folded, in which the folded conformation is calculated to be 18.8 kcal/mol more stable than the extended one (Figure 2a). In the folded form, the geometry of the two phenylsulfonyl groups is asymmetric, with C–O–S bond angles of 120 and 117°, O–S–C bond angles of 95 and 104°, and C–O–S–C torsional angles of 146 and 29°, respectively. Additionally, one phenylene ring of the phenylsulfonate units fits into the U-shaped cavity of the pentiptycene scaffold with weak C–H···π interactions (3.33–3.92 Å) (Figure 2b). The two NCNPt cores exhibit partial π–π stacking (∼10% overlap) with a small dihedral angle (∼2°) and a plane-to-plane distance of 3.7–3.9 Å (Figure 2c). These intramolecular C–H···π and π–π interactions contribute to the stabilization of the folded conformation over the extended form. However, Pt­(II)–Pt­(II) interactions are negligible due to the relatively long Pt–Pt separation of ∼6.11 Å. These observations suggest that the folded form is more likely to exhibit excimer emission than MMLCT emission (vide infra).

**Figure 2:**
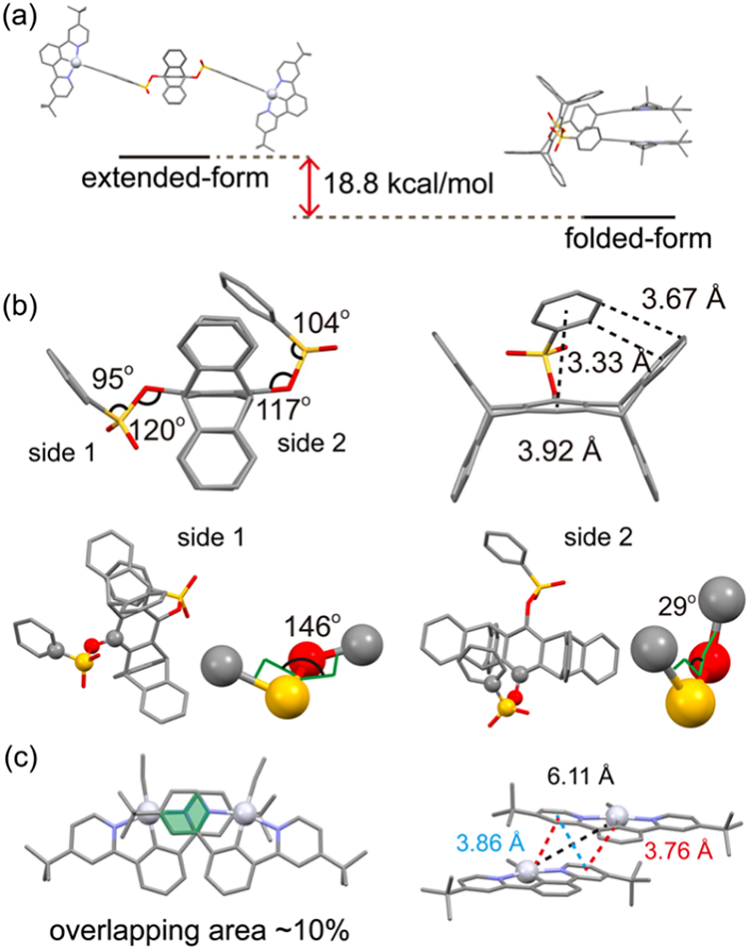
DFT-optimized molecular structure of **2**: (a) energy difference between the extended and folded conformations; selected structural parameters of (b) the BPSP linker and (c) the NCNPt-acetylide chromophore in the folded conformation.

The ROESY NMR spectrum of **2** in CD_2_Cl_2_ also supports a folded conformation of the BPSP linker (Figure S1). The doublet signals at δ8.13 and δ7.84 for the phenylsulfonate groups exhibit cross-peaks with the protons of the pentiptycene peripheral rings at δ7.28−δ7.32 and the bridgehead protons at δ5.73, consistent with the DFT-predicted geometry (side 2 in Figure 2b). Interestingly, the corresponding cross-peaks are also observed for **3** in CD_2_Cl_2_ (Figure S2), indicating that the folding observed in **2** is primarily driven by the intrinsic conformational preference of the BPSP linker, rather than by the π–π interactions of the NCNPt centers.

Regarding the solid-state structure, suitable single crystals of **2** were obtained for X-ray crystallographic analysis via slow evaporation of a dichloromethane/methanol (DCM/MeOH) mixed solvent, whereas **3** did not yield crystals of sufficient quality. Complex **2** crystallizes in the *P*1̅ space group with an extended molecular conformation (Figure 3a), and the crystallographic data are summarized in Table S1. The centrosymmetric molecular structure features a C–O–S–C dihedral angle of 162°, a C–O–S bond angle of 116°, and O–S–C bond angles of 98°. Intermolecular interactions are primarily governed by pairwise π–π stacking between adjacent NCNPt cores, with an interplanar distance of approximately 3.655 Å and an overlapping area of around 44% (Figure 3b). The relatively large Pt–Pt distance of 5.608 Å suggests that Pt­(II)–Pt­(II) interactions are negligible. Additionally, the *tert*-butyl groups fit into the U-shaped cavities of neighboring pentiptycene units, forming the characteristic C–H···π interactions commonly observed in alkyl-pentiptycene crystal packing.^25–30^ Interestingly, the crystal structure is porous, with channels measuring approximately 14.7 Å × 8.5 Å and a total void volume of 26.8% per unit cell (Figure 3c). These voids are occupied by DCM molecules within the crystal lattice.

**Figure 3:**
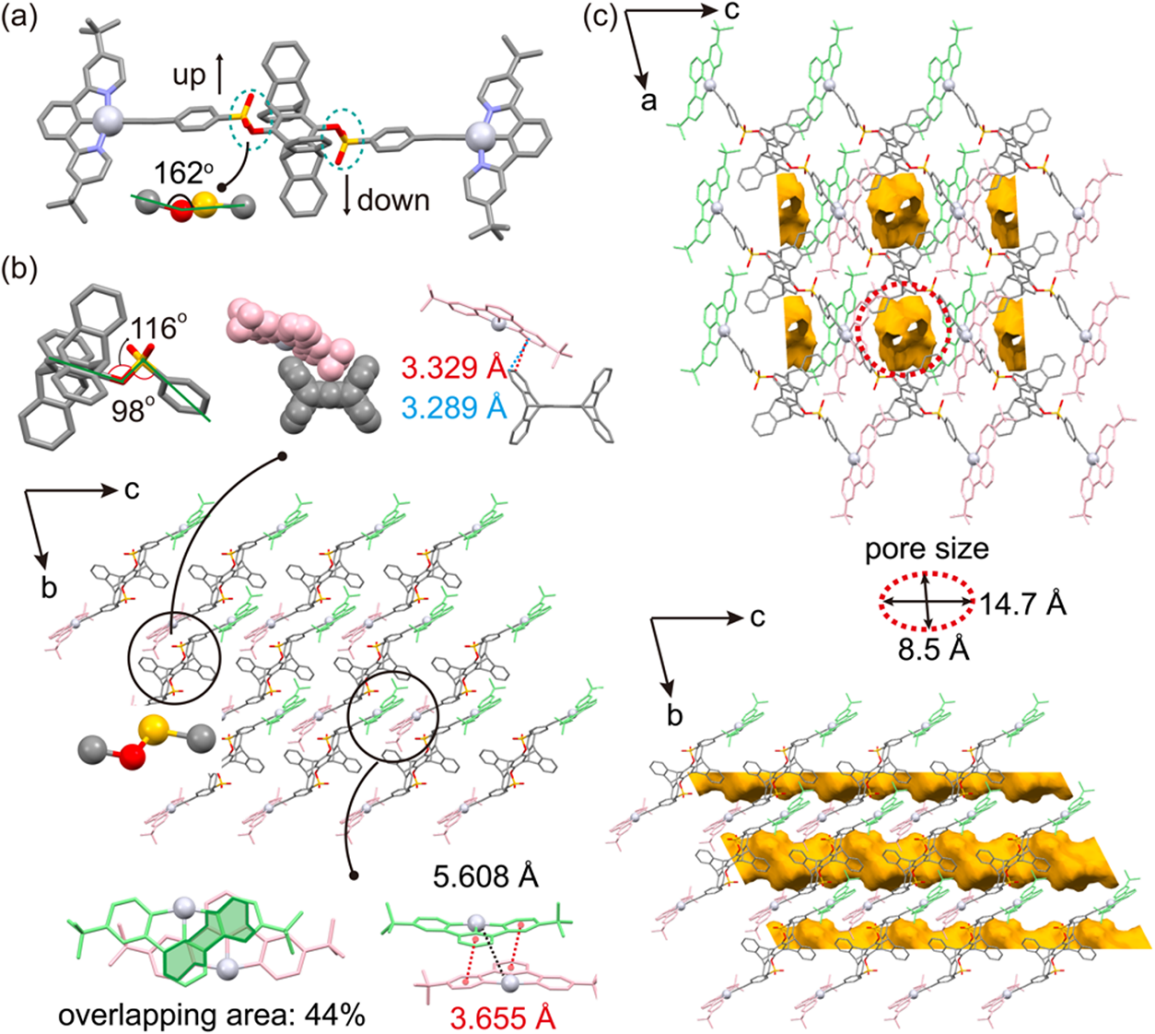
(a) Crystal structure of **2**, showing the extended molecular conformation; (b) crystal packing highlighting tongue-and-groove connectivity and intermolecular π–π interactions; (c) two views of one-dimensional porous channels.

### Photoluminescence Properties

The absorption and emission spectra of complexes **2** and **3** in tetrahydrofuran (THF, 10 μM) are shown in Figure 4a, with the corresponding photophysical data summarized in Table 1. A comparison of the absorption spectra in THF reveals nearly identical features for both complexes, characterized by a strong absorption band in the 250–350 nm region and a moderate, long-wavelength band between 350 and 450 nm. This similarity, particularly in the long-wavelength region, suggests that intramolecular ground-state interactions between the two NCNPt cores are negligible in **2**. Additionally, the absorption spectra of **2** and **3** show minimal dependence on solvent polarity (Figures 4b and S3). Like the absorption spectra, the emission spectra of **2** also show little solvent dependence, except in DMSO (Figure 4c). In solvents such as THF, 1,4-dioxane, DCM, ethyl acetate, and dimethylformamide (DMF), the emission spectra of **2** feature a weak, structured band in the 470–600 nm region and a dominant, structureless band with a maximum (λ_p_) near 663 nm. These two emission bands share a common excitation spectrum (Figure 4d), indicating the same electronically excited state origin. In contrast, complex **3** emits only in the 470–600 nm region in THF (Figure 4a). Notably, despite the distinct emission spectra of **2** and **3**, their emission quantum yields (Φ_p_) in THF are identical (62%). In DMSO, both **2** and **3** exhibit only the higher-energy structured emission band. This change is accompanied by a significant reduction in emission quantum yield: 9% for **2** and 6% for **3**. A similar trend is observed in the luminescence lifetimes (τ_p_). In THF, the lifetimes for **2** are 0.22 and 3.82 μs at 490 nm and 1.56 μs at 663 nm, while **3** shows a lifetime of 3.70 μs at 492 nm (Table 1). However, in DMSO, the lifetimes decrease to 0.027 and 0.689 μs (monitored at 500 nm) for **2** and 0.0015 and 0.030 μs for **3**. The quenching mechanism is likely associated with DMSO chelation to the Pt cores.^36,37^

**Figure 4:**
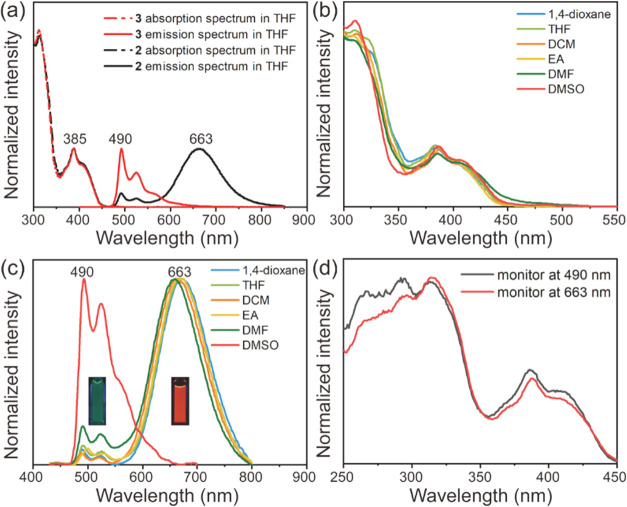
(a) Absorption and emission spectra of **2** and **3** in THF; (b) absorption spectra and (c) emission spectra of **2** in different solvents; (d) excitation spectra of **2** monitored at 490 and 663 nm.

**Table 1:** Photophysical Data of **2** and **3** in Solution and Solid-State

compound	λ_abs_ ^a^ ^,^ ^b^ (nm)	λ_p_ ^a^ ^,^ ^c^ (nm)	Φ_p_ ^a^ (%)	τ_p_ ^d^ ^,^ ^e^ (μs)
**2** in THF	385	490 (663)	62	0.43^f^ (1.56)^g^
**2** in DMSO	387	500	9	0.031^h^
**2**_**Crystal**_		505 (653)	10	0.32 (0.90)
**2**_**Gound**_ ^k^		500 (664)	23	0.13 (0.74)
**2**_**PhH**_ ^l^		502 (658)	14	0.14 (1.11)
**2**_**EA**_ ^m^		502 (660)	11	0.06 (0.70)
**3** in THF	385	492	62	3.70^i^
**3** in DMSO	387	492	6	0.0099^j^
**3**_**Powder**_		489 (621)	9	0.95 (1.45)
**3**_**Gound**_ ^k^		496 (660)	35	0.15 (1.00)
**3**_**EA**_ ^m^		491 (650)	17	0.75 (1.38)

a Excitation at 390 nm.

b Maximum of absorption spectrum.

c Maximum of monomer emission; excimer maximum shown in parentheses.

d Average lifetime, calculated as ∑_*i*_^*n*^ *A_i_* τ*_i_*/∑_*i*_^*n*^ *A*_*i*_, where *A_i_* and τ*_i_* are the amplitude and lifetime of each component, respectively. Individual components for the solution phase are provided as footnotes; those for the solid state are listed in Table S4.

e Excitation using a 405 nm pulsed laser.

f τ_1_ = 0.22 (*A*_1_ = 0.63) and τ_2_ = 3.82 (*A*_2_ = 0.04).

g τ_1_ = 0.20 (*A*_1_ = −0.82) and τ_2_ = 1.56 (*A*_2_ = 0.84).

h τ_1_ = 0.027 (*A*_1_ = 0.50) and τ_2_ = 0.69 (*A*_2_ = 0.003).

i Single component (monoexponential decay).

j τ_1_ = 0.0015 (*A*_1_ = 0.24) and τ_2_ = 0.030 (*A*_2_ = 0.10).

k Sample after mechanical grinding.

l Sample after fuming with benzene vapor.

m Sample after fuming with ethyl acetate vapor.

Time-dependent density functional theory (TDDFT) calculations and natural transition orbital (NTO) analyses for complexes **2** (in folded form) and **3** based on their DFT optimized structures in both singlet and triplet excited states are summarized in Table S2. For both complexes, the high-energy intense absorption band is primarily attributed to locally excited (LE) π–π transitions of the phenylacetylene moiety. In contrast, the lower-energy absorption band displays greater charge-transfer character, including intraligand charge transfer (ILCT) and ligand-to-metal charge transfer (LMCT) contributions. Notably, the low-energy transitions involve only the NCNPt-acetylide moiety (i.e., the chromophore), and the BPSP linker is nearly silent. Although the orbital configurations differ significantly between **2** and **3**, their predicted absorption maxima are similar: namely, 329 and 271 nm for **2**, and 327 and 273 nm for **3**, which aligns well with the experimental data. In the T_1_ state, complex **3** exhibits a ligand-centered (LC) nature in the NCNPt-acetylide chromophore, whereas **2** exhibits an intraligand charge transfer (ILCT) nature across the two NCNPt cores.

Several pieces of evidence indicate that the short- and long-wavelength emission bands of **2** in THF can be assigned to the monomer and intramolecular excimer emissions of the NCNPt-acetylide chromophore, respectively. First, the emission band at 490 nm exhibits both a spectral profile and a lifetime (3.82 μs) that closely match those of the 492 nm emission band of **3** in THF (3.70 μs). Second, excimer formation aligns with the preferred folded conformation predicted by DFT calculations (Figure 2). Third, at a concentration of 1 × 10^–5^ M in THF, the decay profile of the 663 nm band for **2** shows two components, one with a lifetime of 0.22 μs and a negative pre-exponential factor, indicative of a rise time. This rise time closely matches the 0.20 μs decay observed at 490 nm, consistent with the precursor–successor dynamics typical of excimer formation (Table 1). This assignment is further supported by the negligible ground-state interactions between the chromophores and by the nearly identical excitation spectra recorded at 663 and 490 nm (Figure 4d). The excimer nature of the 663 nm band in THF also explains its absence in DMSO: specifically, the monomer lifetime in DMSO is too short (∼0.031 μs) to allow excimer formation (∼0.20 μs). To rule out any contribution from intermolecular excimer emission to the 663 nm band, we examined possible concentration effects. As the concentration of **2** increased from 1 to 100 μM, the absorption at 410 nm followed the Beer–Lambert law, and the intensity ratio of the monomer to excimer bands remained essentially unchanged (Figure S4a). For comparison, no intermolecular excimer emission is observed for **3** up to 100 μM (Figure S4b). Together, these results confirms that the red-shifted band at 663 nm observed for **2** originates from intramolecular excimer emission. Since the emission spectrum of **2** in THF is dominated by this excimer emission, its high quantum yield (Φ_p_ = 62%) indicates that the intramolecular excimer is highly emissive, unlike typical cases where π–π stacking interactions leads to emission quenching.^38,39^ The strongly emissive nature of the intramolecular excimer makes **2** a promising candidate for stimuli-induced excimer–monomer switching in THF solution (vide infra).

In the solid state, both **2** (as a crystal, **2**_**Crystal**_) and **3** (as a powder, **3**_**Powder**_) exhibit two emission bands, an intense, structured band near 490 nm and a slightly weaker, broad band near 650 nm (Figure 5). The resemblance of these bands to the monomer and excimer emissions of **2** in THF suggests that the structured band originates from monomer emission, while the broad band has a bimolecular nature. Because the monomer emission dominates, the overall emission color appears green for both **2**_**Crystal**_ and **3**_**Powder**_. The excitation spectra recorded at the broad band (653 nm for **2**_**Crystal**_ and 621 nm for **3**_**Powder**_) are red-shifted compared to those recorded at the monomer band (505 nm for **2**_**Crystal**_ and 489 nm for **3**_**Powder**_), indicating the presence of ground-state intermolecular interactions. This interpretation is supported by TDDFT calculations on two neighboring molecules in the crystal structure of **2**, which reveal orbital interactions between adjacent chromophores (Table S3). Such ground-state interactions may give rise to dimer emission (i.e., without further excited-state structural relaxation) or promote excimer emission (i.e., with additional enhancement of intermolecular interactions in the excited state) to account for the broad band. In general, dimer emission from Pt­(II) complexes resulting in broad bands is attributed to the formation of a MMLCT state.^40^ However, given the absence of a well-defined MMLCT absorption band, the relatively large Pt–Pt distance of 5.608 Å, and the dominance of monomer emission in **2**_**Crystal**_, the contribution of MMLCT emission to the broad band is likely negligible. Alternatively, the “preassociated” dimer may further approach in the excited state to form an excimer, similar to the process observed for excimer formation in solution. Since solid-state molecular mobility depends on lattice rigidity and the strength of intermolecular interactions, this could explain why the broad band emission occurs at shorter wavelengths (653 nm for **2**_**Crystal**_ and 621 nm for **3**_**Powder**_) than in solutions (663 nm) and why the peak positions differ slightly between **2**_**Crystal**_ and **3**_**Powder**_. Although these solid-state excimers differ from conventional excimers, which involves repulsive ground-state interactions, “excimer emission” is used hereafter for simplicity to describe the broad band of **2**_**Crystal**_ and **3**_**Powder**_. For comparison, emission and excitation spectra of poly­(methyl methacrylate) (PMMA) films doped with **2** (1–5 w%) were recorded, showing only monomer emission and excitation (Figure S5). This indicates that intramolecular excimer formation is either inhibited in the PMMA matrix (if **2** remains folded) or that **2** adopts an unfolded conformation.

**Figure 5:**
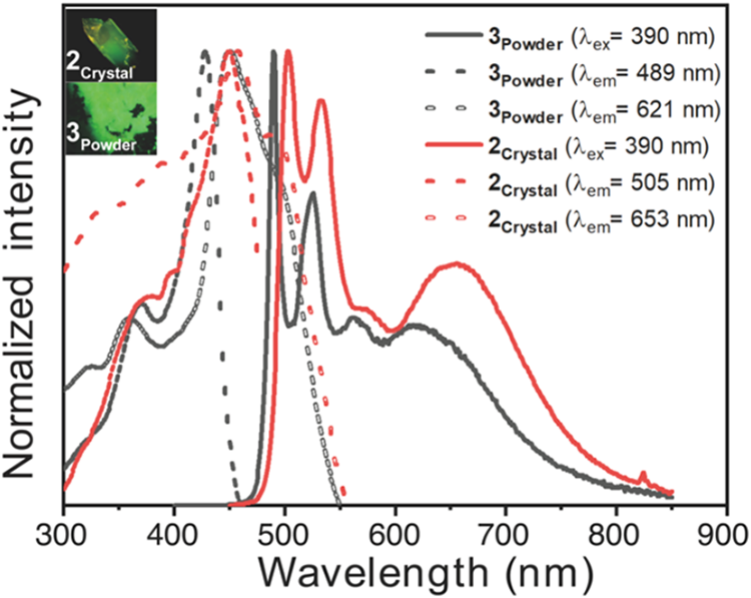
Excitation and emission spectra of **2**_**Crystal**_ and **3**_**Powder**_. Insets: luminescence images of the corresponding samples.

### Stimuli-Responsive Luminescence in Solution

The strong excimer emission for **2** in most of the tested organic solvents provides an opportunity for stimuli-responsive luminescence in solutions based on the mechanism of excimer-to-monomer switching. First, upon lowering the temperature of the solution of **2** in 2-methyltetrahydrofuran (MTHF), we observed a growth of the green monomer emission at the expense of the excimer emission, leading to nearly disappearance of the excimer emission band at 180 K (Figure 6). Notice that temperature has little effects on the excitation spectra, and the spectra recorded at λ_em_ = 490 nm are similar to that at 663 nm in all temperatures (Figure S6), denoting a temperature-induced switching between monomer and excimer emission. These results indicate that excimer formation is a thermally activated process, and the barrier for excimer formation cannot be overcome at low temperatures (<180 K).

**Figure 6:**
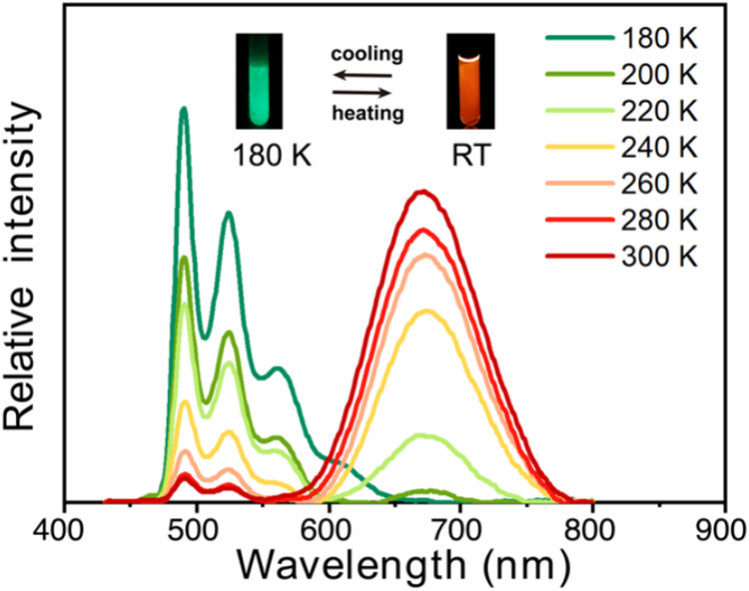
Temperature-dependent emission spectra of **2** in 2-Methyltetrahydrofuran. Inset: luminescence images at room temperature (RT) and 180 K.

The red excimer emission of **2** in THF can also be switched to green monomer emission by Ag­(I) and Cu­(I) ions (Figure 7). Among the tested cations, including Li­(I), Na­(I), K­(I), NH_4_^+^, Ag­(I), Cu­(I), Cu­(II), and Zn­(II), only Ag­(I) and Cu­(I) induce a complete switching from excimer to monomer emission. While Cu­(II) and Zn­(II) cause a slight enhancement of the monomer emission, resulting in orange color, the other metal ions and ammonium do not perturb the emission spectra. The corresponding absorption spectra show a clear blue shift in the presence of Ag­(I), Cu­(I), Cu­(II), and Zn­(II), but not with the other tested species (Figure S7). Additionally, Job plot analyses of luminescence at different Ag­(I) and Cu­(I) concentrations indicate a 1:1 binding stoichiometry between **2** and each metal ion (Figure S8). These effects of Ag­(I) and Cu­(I) arise from conformational perturbation and “locking”, which prevent excimer formation. Notably, the monomer emission spectrum induced by Ag­(I) is structureless, suggesting perturbation of the NCNPt-acetylide chromophore. This likely arises from interactions between the acetylene units and Ag­(I),^14,41,42^ as depicted in the inset of Figure 7. In contrast, the Cu­(I)-induced monomer emission is structured, similar to the monomer bands observed in the presence of Cu­(II) and Zn­(II), implying that these ions may interact with the BPSP linker rather than the chromophore itself. The much lower emission quantum yield for the Ag­(I)-induced monomer emission (3%) compared to the Cu­(I)-induced one (15%) indicates that Ag­(I)-acetylide interactions promote nonradiative decay of the chromophore. The stronger effect of Ag­(I) compared to Cu­(I), Cu­(II), and Zn­(II) is likely due to its larger ionic radius (1.00 Å vs 0.60 Å vs 0.57 Å and 0.60 Å, respectively)^43^ and its lower charge density (i.e., its greater “softness” as a Lewis acid).

**Figure 7:**
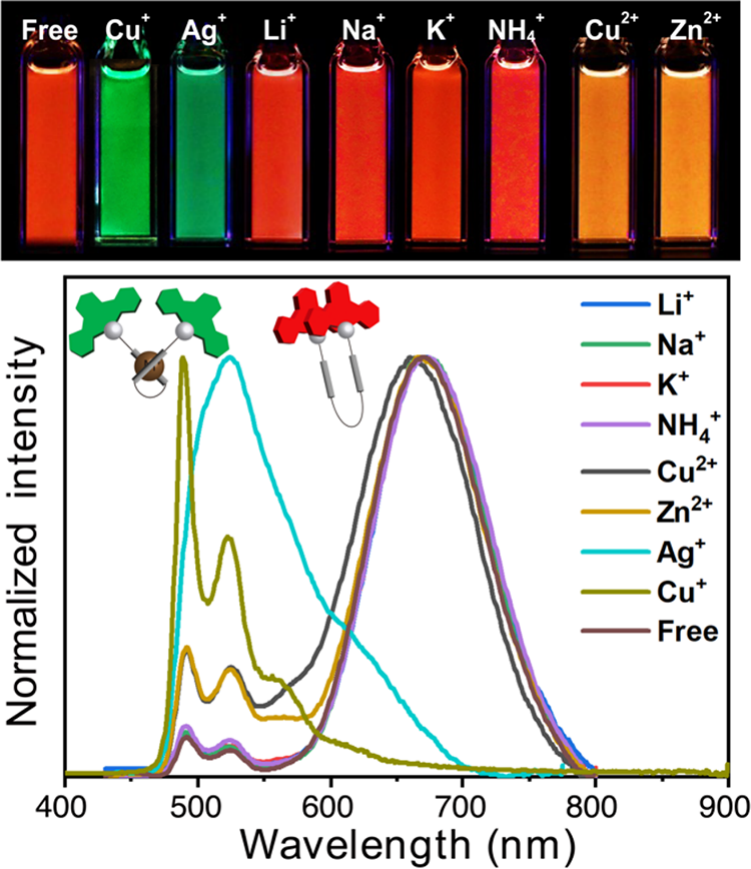
Photographs (top) of **2** in THF with various metal ions and the nonmetal ion NH_4_^+^, along with their corresponding emission spectra (bottom). Each salt (LiBF_4_, NaBF_4_, KBF_4_, NH_4_BF_4_, CuI, Cu­(NO_3_)_2_, Zn­(BF_4_)_2_ and AgBF_4_) was added at ∼10 equiv. Inset: proposed bonding mode between **2** and the Ag^+^ ion and the excimer state.

In addition to cooling and Ag­(I) or Cu­(I) addition, excimer formation for **2** in THF can also be perturbed by small amounts of DMSO or O_2_. As shown in Figure 8a, titration of **2** in THF (3 mL) with 5–15 μL DMSO causes a pronounced decrease in the excimer emission band, while the monomer emission band remains largely unaffected. When the amount of DMSO reaches 150 μL or more, the monomer emission intensity also begins to decrease. At 500 μL of DMSO (0.17% v/v), the excimer emission is nearly quenched, resulting in a luminescence color change from red to green. Figure 8b shows that molecular oxygen (0–100 μL) induces greater quenching of excimer band relative to the monomer band, leading to red-to-yellowish green switching. Given that the excimer formation for **2** in THF occurs within ∼0.20 μs at ambient temperature (Table 1), it is inhibited when the excited monomer precursor has a short lifetime (e.g., 0.031 μs in DMSO). While shortening of the monomer excited-state lifetime also reduces the monomer emission intensity, inhibition of excimer formation imposes a larger quenching effect on the excimer band, explaining the greater quenching of excimer relative to monomer emission.

**Figure 8:**
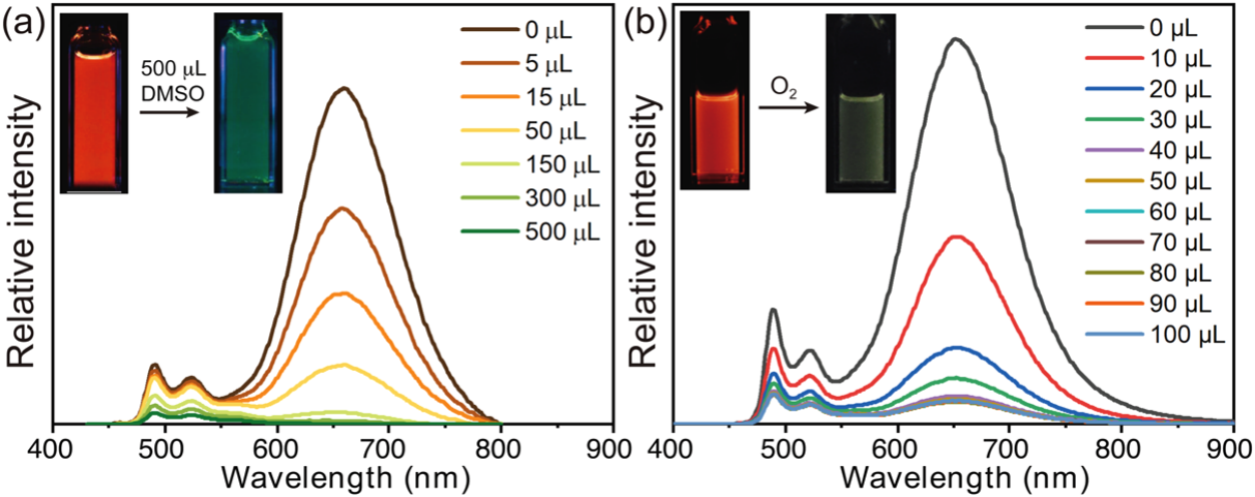
Luminescence spectra of **2** in THF in the presence of varying amounts of (a) DMSO and (b) O_2_. Insets: photographs of the solutions before and after the addition of DMSO or O_2_.

For comparison, **3** does not exhibit luminescence color changes in response to these stimuli, consistent with the absence of intramolecular excimer emission under the same conditions (Figure S9). The multistimuli-responsive luminescence behavior observed in **2**, but not in **3**, highlights the utility of the BPSP linker in constructing Pt­(II)-based stimuli-responsive luminescent materials.

### Stimuli-Responsive Luminescence in Solid State

The solid-state emission behavior of complexes **2** and **3** in response to external stimuli is illustrated in Figure 9. For complex **2**, the crystalline form (**2**_**Crystal**_) exhibits green emission, which undergoes a pronounced shift to red upon grinding (**2**_**Ground**_), demonstrating a clear mechanochromic luminescence (MCL) response (Figure 9a). Subsequent exposure to either benzene (PhH) or ethyl acetate (EA) vapor partially restores the monomer emission, resulting in orange luminescence (**2**_**PhH**_ and **2**_**EA**_). Due to the lack of suitable single crystals for **3**, its powder form (**3**_**Powder**_) was used to investigate stimuli-responsive behavior. As shown in Figure 9b, **3**_**Powder**_ also displays MCL characteristics: it transitions from green to red-emission upon grinding (**3**_**Ground**_), while fuming with EA vapor restores the green monomer emission. For both complexes, these external stimuli affect ground-state intermolecular interactions, as confirmed by excitation spectra monitored at the monomer and excimer emission bands (Figure S10). The excitation profiles exhibit varying degrees of change, indicating modifications in molecular packing. These structural rearrangements alter the relative contributions of monomer and excimer emissions, resulting in the observed emission switching.

**Figure 9:**
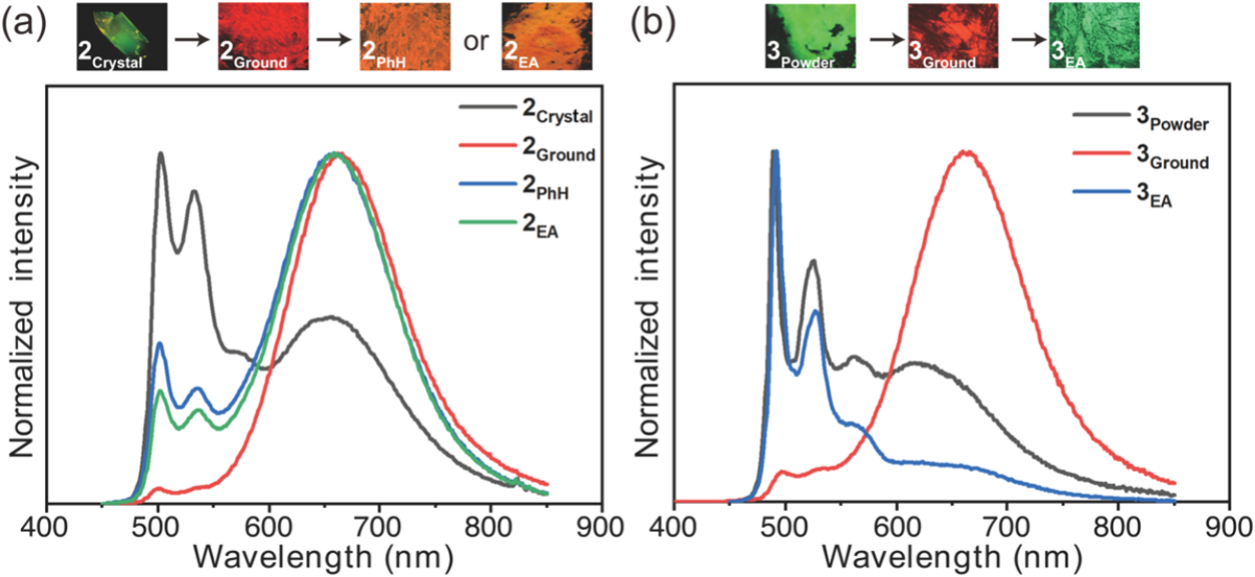
Luminescence images and emission spectra of (a) **2**_**Crystal**_ and (b) **3**_**Powder**_ before and after mechanical grinding, followed by fuming with benzene (PhH) or ethyl acetate (EA) vapor.

The relative quantum yield of excimer vs monomer emission in the solid state warrants further discussion. Upon transitioning from **2**_**Crystal**_ to **2**_**Ground**_, the overall emission quantum yield (Φ_p_) increases from 10 to 23%, indicating that the solid-state intermolecular excimer emission is more efficient than the monomer emission. This behavior contrasts with that in THF solution, where the monomer and intramolecular excimer emission exhibit comparable quantum efficiencies. This difference may arise from two main factors. First, the nature of the excimer differs between the two phases, not only in being intramolecular (THF) vs intermolecular (in the solid state), but also in the orientation of the NCNPt-acetylide chromophores: syn in solution (Figure 2) vs anti in the crystal (Figure 3). Second, in the solid phase, the monomer excited state may be more prone to aggregation-caused quenching than the excimer excited state. Support for enhanced intermolecular interactions upon grinding comes from the red-shift observed in the excitation spectrum monitored at 660 nm, relative to that at 490 nm (Figure S10), indicating stronger ground-state π–π interactions in the excimer region after mechanical treatment. This higher excimer efficiency also explains the reduction in Φ_p_ upon exposure of **2**_**Ground**_ to EA and PhH vapors: Φ_p_ decreases from 23 to 14% (**2**_**PhH**_) and 11% (**2**_**EA**_), corresponding to a relative increase in monomer emission intensity. A similar trend is observed for **3**. Upon grinding, Φ_p_ increases from 9% (**3**_**Powder**_) to 35% (**3**_**Ground**_), accompanied by changes in the excitation spectrum. Fuming with EA reverses this effect, decreasing Φ_p_ from 35 to 17% (**3**_**EA**_), again correlating with enhanced monomer emission. Notably, the emission lifetimes of both monomer and excimer states remain largely unchanged throughout these treatments, indicating that the observed luminochromic responses result primarily from a straightforward monomer–excimer switching mechanism.

## Conclusions

We have demonstrated that complex **2** exhibits dual-phase, multistimuli-responsive luminescence through the interplay between high-contrast monomer (green) and excimer (red) emissions. In THF solution, excimer-to-monomer switching can be triggered by lowering the temperature or by adding Ag­(I), DMSO, or O_2_, all of which disrupt intramolecular excimer formation promoted by the prefolded molecular conformation. In contrast, in the solid phase, switching from monomer to intermolecular excimer emission is induced by mechanical grinding and can be partially reversed by fuming with benzene or ethyl acetate vapors. The BPSP linker’s ability to adopt a folded conformation in solution and an extended conformation in the solid state plays a critical role in enabling these responsive luminochromic behaviors. Altogether, our findings not only provide a new example of DPLR (dual-phase luminochromic response) in Pt­(II) complexes but also highlight the utility of the rigid H-shaped pentiptycene scaffold in designing multistimuli-responsive luminescent materials.

## Experimental Section

### Materials and Chemical Safety Information

All commercial reagents, catalysts, solvents (HPLC grade for photophysical measurements) were used as received. Column chromatography was carried out on silica gel (Geduran SI 60). No unusual safety precautions were required for this work beyond standard laboratory practices. Many of the chemicals used are potentially hazardous and were therefore handled in small quantities under a fume hood, in accordance with standard safety protocols for organic and inorganic synthesis. Notably, 4-bromobenzenesulfonyl chloride can release SO_2_ and HCl vapors upon reaction, and thus must be handled with particular care under a fume hood.

### Synthesis of Complex **2**

A mixture of compound **6** (100 mg, 0.13 mmol), compound **7** ^29^ (152 mg, 0.27 mmol) and CuI (1.2 mg, 6 μmol) in degassed CH_2_Cl_2_ (5 mL) and diisopropylamine (8 mL) was stirred at 60 °C for 48 h under nitrogen atmosphere. After cooling to room temperature, the mixture was filtered and washed with water, methanol, and diethyl ether. The collected solid was precipitated with CH_2_Cl_2_ /methanol to afford **2** as yellow solid (77 mg, 33%). Mp: >300 °C; ^1^H NMR (400 MHz, CD_2_Cl_2_): δ = 9.31–9.20 (m, 4H), 8.13 (d, *J* = 8.6 Hz, 4H), 7.84 (d, *J* = 8.6 Hz, 4H), 7.72 (d, *J* = 2.0 Hz, 4H), 7.58 (d, *J* = 7.7 Hz, 4H), 7.32–7.28 (m, 8H), 7.27–7.24 (m, 6H), 7.00–6.95 (m, 8H), 5.73 (s, 4H), 1.42 (s, 36H); ^13^C­{^1^H} NMR (100 MHz, CD_2_Cl_2_): δ = 178.4, 169.7, 164.4, 155.0, 148.5, 145.0, 144.2, 139.5, 138.7, 136.7, 133.0, 132.0, 128.8, 125.8, 124.8, 124.1, 123.8, 121.6, 117.4, 110.9, 49.7, 36.1, 30.7; IR (KBr): 670, 754, 819, 859, 972, 1092, 1169, 1232, 1379, 1460, 1485, 1581, 1615, 2076, 2962 cm^–1^; HRMS (ESI-TOF): *m*/*z* calculated for C_98_H_83_N_4_O_6_Pt_2_S_2_^+^ ([M + H]^+^): 1865.5045. Found: 1865.5060.

### Synthesis of Compound **4**

Pentiptycene hydroquinone (1 g, 2.16 mmol) and K_2_CO_3_ (598 mg, 4.32 mmol) were stirred in acetone (50 mL) at 50 °C for 30 min under a nitrogen atmosphere. 4-bromobenzenesulfonyl chloride (1.94 g, 7.57 mmol) in acetone (15 mL) was then slowly added to the mixture. The reaction was stirred at 75 °C for 5 days. After cooling to room temperature, the mixture was poured into water (600 mL). The resulting white precipitate was filtered, washed with water, methanol, diethyl ether, and hexane, and dried under vacuum to yield compound **4** as a white solid (1.58 g, 81%). Mp: >300 °C; ^1^H NMR (400 MHz, CD_2_Cl_2_): δ = 8.11 (d, *J* = 8.6 Hz, 4H), 7.94 (d, *J* = 8.6 Hz, 4H), 7.25–7.20 (m, 8H), 6.97–6.92 (m, 8H), 5.60 (s, 4H); ^13^C­{^1^H} NMR (100 MHz, CD_2_Cl_2_): δ = 144.8, 140.0, 138.4, 136.0, 133.9, 130.8, 130.5, 126.0, 124.7, 49.7; IR (KBr): 751, 764, 820, 865, 971, 1008, 1068, 1091, 1175, 1194, 1231, 1352, 1391, 1460, 1574, 3040, 3068 cm^–1^; HRMS (ESI-TOF): *m*/*z* calculated for C_46_H_29_Br_2_O_6_S_2_^+^ ([M + H]^+^): 898.9767. Found: 898.9758.

### Synthesis of Compound **5**

A mixture of compound **4** (1.5 g, 1.67 mmol), Pd­(PPh_3_)_2_Cl_2_ (58.4 mg, 0.08 mmol), CuI (16 mg, 0.08 mmol), and trimethylsilylacetylene (1.17 mL, 8.32 mmol) in degassed DMF (8 mL) and diisopropylamine (50 mL) was refluxed at 90 °C for 18 h under a nitrogen atmosphere. DMF and diisopropylamine were purged for 20 min prior to use. After cooling to room temperature, the mixture was extracted with water and CH_2_Cl_2_. The organic layer was collected, dried over MgSO_4_, filtered, and concentrated under reduced pressure. The crude product was purified by silica gel column chromatography using hexane/CH_2_Cl_2_ (6:1, v/v) and then hexane/CH_2_Cl_2_ (3:1, v/v) as the eluent, yielding compound **5** as a white solid (765 mg, 50%). Mp: >300 °C; ^1^H NMR (400 MHz, CDCl_3_): δ = 8.12 (d, *J* = 8.6 Hz, 4H), 7.78 (d, *J* = 8.6 Hz, 4H), 7.21–7.17 (m, 8H), 6.95–6.91 (m, 8H), 5.60 (s, 4H), 0.33 (s, 18H); ^13^C­{^1^H} NMR (100 MHz, CDCl_3_): δ = 144.1, 138.8, 138.2, 135.8, 133.0, 130.0, 128.3, 125.3, 124.2, 102.7, 100.4, 49.1, −0.27; IR (KBr): 755, 813, 865, 973, 1089, 1177, 1229, 1255, 1352, 1459, 1592, 2157, 2955, 3070 cm^–1^; HRMS (ESI-TOF): *m*/*z* calculated for C_56_H_47_O_6_S_2_Si_2_^+^ ([M + H]^+^): 935.2347. Found: 935.2330.

### Synthesis of Compound **6**

Compound **5** (190 mg, 0.20 mmol) was dissolved in a mixed solution of THF (12 mL) and methanol (3 mL). K_2_CO_3_ (70 mg, 0.51 mmol) was then added, and the mixture was stirred at room temperature for 1 h. After removing the volatile solvent under reduced pressure, the residue was extracted with saturated brine and CH_2_Cl_2_. The collected organic layer was dried over MgSO_4_, filtered, and concentrated under reduced pressure. The crude product was purified by silica gel column chromatography using hexane/CH_2_Cl_2_/EA (5:1:0.05, v/v/v) as the eluent, yielding compound **6** as a white solid (140 mg, 87%). Mp: >300 °C; ^1^H NMR (400 MHz, CD_2_Cl_2_): δ = 8.22 (d, *J* = 8.5 Hz, 4H), 7.90 (d, *J* = 8.5 Hz, 4H), 7.24–7.22 (m, 8H), 6.96–6.93 (m, 8H), 5.65 (s, 4H), 3.50 (s, 2H); ^13^C­{^1^H} NMR (100 MHz, CD_2_Cl_2_): δ = 144.8, 139.6, 138.4, 136.8, 134.0, 129.6, 129.0, 125.9, 124.7, 82.6, 82.1, 49.7; IR (KBr): 756, 821, 866, 974, 1090, 1173, 1229, 1306, 1380, 1459, 1595, 2120, 3073 cm^–1^; HRMS (ESI-TOF): *m*/*z* calculated for C_50_H_31_O_6_S_2_^+^ ([M + H]^+^): 791.1557. Found: 791.1557.

## Supplementary Material



## Notes

Experimental and computational details, materials, and methods, including crystallographic data, electronic absorption, excitation, and emission spectra of **2** and **3**, ^1^H and ^13^C­{^1^H} NMR spectra for all new compounds, and Cartesian coordinates of DFT-optimized structures of the complexes (PDF)
