# Supplementary material for: Dual-Phase Multi-Stimuli-Responsive Luminescence from a Pentiptycene-Linked Binuclear Cyclometalated Platinum(II) Complex
Source: Inorg Chem. 2025 Jul 31;64(32):16527–36. doi: 10.1021/acs.inorgchem.5c02560 (PMC12365873; doi:10.1021/acs.inorgchem.5c02560)
Supplement: Supplementary file 1 [file ic5c02560_si_001.pdf]

(Supporting Information)

Dual-Phase Multi-Stimuli-Responsive Luminescence from a Pentiptycene-Linked Binuclear Cyclometalated Platinum(II) Complex

Ying-Feng Hsu [a], Yu-Chieh Ho [a], Yi-Hung Liu [a], and Jye-Shane Yang [a,b*]

[a] Department of Chemistry, National Taiwan University, Taipei 10617, Taiwan

[b] Center for Emerging Material and Advanced Devices, National Taiwan University, Taipei 10617, Taiwan

*Email: jsyang@ntu.edu.tw

Index

S3	**General methods**
S4-S7	**Materials and Synthesis.**
S8	**Table S1.** X-ray crystallographic data of **2**.
S9-S10	**Table S2**. TDDFT-derived energy, oscillator strength (*f*), and natural transition orbital (NTO) pairs with transition character analysis for **3** and the folded form of **2**.
S11-S12	**Table S3**. TDDFT-derived energy, oscillator strength (*f*), and natural transition orbital (NTO) pairs with transition character analysis for the extended form of **2** in crystal structure.
S13	**Figure S1.** 500 MHz ^{1}H–^{1}H ROESY spectrum of compound **2** in CD_2Cl_2.
S14	**Figure S2.** 500 MHz ^{1}H–^{1}H ROESY spectrum of compound **3** in CD_2Cl_2.
S14	**Figure S3.** (a) Absorption and (b) emission spectra of **3** in solvents of different polarity.
S15	**Figure S4.** (a)Absorbance at 410 nm and (b) emission profile of **2** at concentrations of 1−100 μM in THF.
S15	**Figure S5.** Excitation spectra (dashed lines) and emission spectra (solid lines) of compound **2** in PMMA films at 1–5 wt% loadings. Excitation spectra were monitored at 490 nm.
S16	**Figure S6.** Excitation spectra of **2** at different temperatures in 2-methyltetrahydrofuran, monitored at (a) 490 nm and (b) 662 nm.
S16	**Figure S7.** Absorption spectra of **2** with the addition of (a) Cu^{2+}, Zn^{2+}, Ag^+ and Cu^+, and (b) Li^+, Na^+, K^+, and NH_4^+, compared to the stock solution.

S17	**Figure S8.** Emission spectra of compound **2** in degassed THF with varying equivalents of (a) Ag^+ and (b) Cu^+ ions (left), and corresponding Job plots of emission intensity at 520 nm (right).
S18	**Figure S9.** Emission spectra for the stimuli-responsive properties of **3** under various conditions: (a) temperature variation, (b) addition of silver ions, (c) addition of DMSO, and (d) exposure to oxygen.
S19	**Figure S10.** Excitation spectra of compound **2** monitored at (a) 490 nm and (b) 663 nm, and compound **3** monitored at (c) 489 nm and (d) 621 nm
S20-S21	**Table S4.** Multiple luminescence lifetimes of **2** and **3** in the solid state under different external stimuli, measured using a 405 nm pulsed laser excitation source.
S22-S39	**Figure S11-28.** ^{1}H-NMR and $^{13}C\{^{1}H\}$-NMR spectra of related compounds.
S40-S57	**Table S5-S8.** Cartesian coordinates of the ground-state and excited-state optimized structures of **2** and **3**.
S58	**References**

Experimental Section

General Methods.

The ^{1}H-NMR and ^{13}C-NMR spectra were recorded using a Bruker AVIII-400 MHz or Bruker AVIII-500 MHz spectrometer. Chemical shifts (δ) are reported in parts per million (ppm) relative to $CDCl_3$ (^{1}H: δ = 7.26, ^{13}C: δ = 77.00) or CD_2Cl_2 (^{1}H: δ = 5.32, ^{13}C: δ = 54.00).

High-resolution mass spectra were obtained using fast-atom bombardment (FAB) ionization with a JEOL JMS-700 spectrometer or electrospray ionization (ESI) with a Bruker microTOF-QII spectrometer.

UV/visible spectra were recorded on a Cary300 double-beam spectrophotometer.

Emission and excitation spectra were measured with an Edinburgh FS5 spectrometer at ambient temperature and corrected for the R980 detector. A solution of coumarin 153 [Φ_f = 0.54 in EtOH] was used as the standard for determining luminescence quantum yield, with compounds in solution purged with nitrogen for 5 minutes. The optical density of all solutions was approximately 0.1 at the excitation wavelength, and an error of 5% was estimated for luminescence quantum yields.

Absolute luminescence quantum yields for solid samples were determined using an integrating sphere (PTFE based material, 150mm inner diameter) with an Edinburgh FS5 spectrometer.

Lifetime measurements were conducted using a picosecond pulsed diode laser (EPL-405) as the light source. The goodness of the nonlinear least-squares fit for phosphorescence was judged by the χ^2 value ($0.8 < \chi^2 < 1.2$).

Luminescence images were captured using an Olympus IX73 inverted microscope equipped with a DP73 color camera. The microscope's light source was a halogen lamp with an Olympus U-FUW filter, passing light in the range of 340–390 nm.

Crystal structure was determined using a Rigaku Oxford Diffraction diffractometer (Xcalibur, Atlas, Gemini) with Mo-Kα radiation (λ = 0.71073 Å).

PMMA film preparation: A 4% w/w solution of poly(methyl methacrylate) (PMMA, averaged Mw = 97,000 g/mol) was first prepared in THF and sonicated to complete dissolution. Dye-doped PMMA samples were then prepared by mixing the PMMA solution with compound **2** at concentrations of 1, 2.5, and 5 wt% relative to the weight of PMMA. After an additional 10 minutes of sonication, the mixtures were drop-cast onto quartz plates and allowed to dry in covered dishes at room temperature.

Computational Method. The density functional theory (DFT) and time dependent density functional theory (TD-DFT) calculations were performed using the Gaussian 16, Reversion A. 03 program package.[S1] For all the calculations, the M06-2X functional[S2] was employed for energy and orbital in the singlet and triplet excited states.

In addition, the effective core potential (ECP) SDD[S3] basis set is used for Pt and the 6-31G(d,p) basis set for the ligand atoms. TDDFT-derived state energy, oscillator strength, configuration interactions, and electronic character of the lowest singlet excited states (S_1) and the lowest triplet excited state (T_1) are used in the same method as DFT. The natural transition orbitals (NTOs)[S4] analysis were performed with Multiwfn [S5].

Materials, and Synthesis

All commercial reagents, catalysts, solvents (HPLC grade for photophysical measurements) were used as received. Column chromatography was carried out on silica gel (Geduran® SI 60). The synthetic schemes for complexes **2** and **3** are shown in Scheme 1 and Scheme S1, respectively.

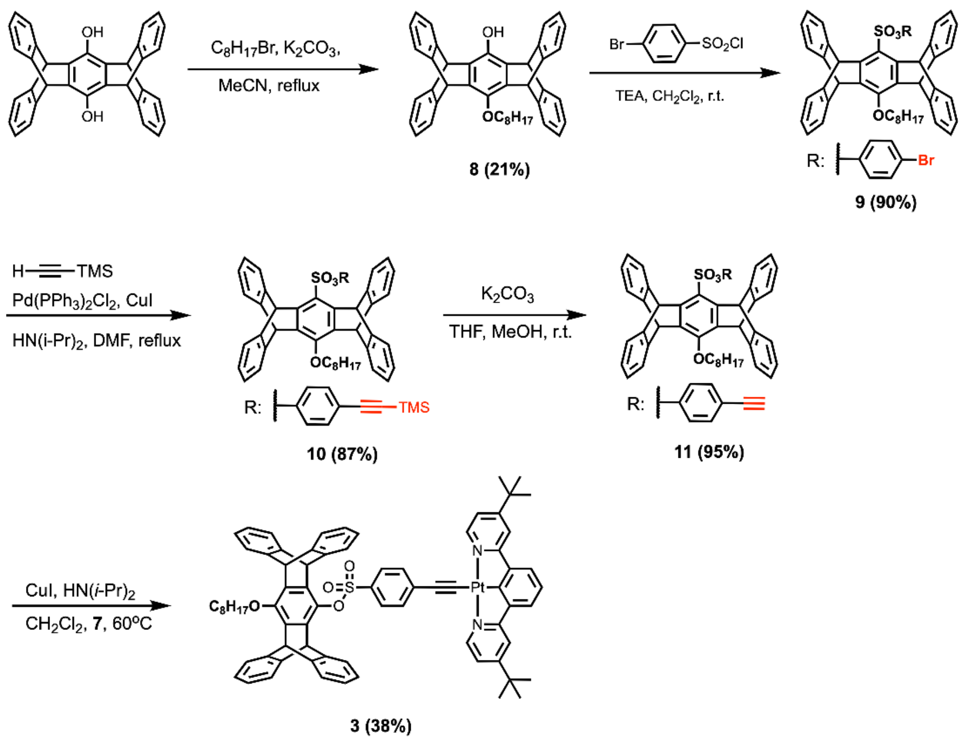

Scheme S1. Synthesis of **3**.

Synthesis of Compound 8

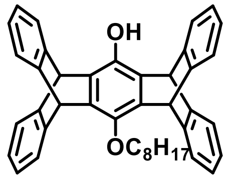

A mixture of pentiptycene hydroquinone (1 g, 2.16 mmol) and K_2CO_3 (448 mg, 3.24 mmol) in acetonitrile (50 mL) was stirred at 60 °C for 30 minutes under nitrogen atmosphere. 1-bromooctane (0.373 mL, 2.16 mmol) in acetonitrile (5 mL) was dropped

into the mixture. The reaction was stirred at 90 °C for another 16 h. After cooling to room temperature, the mixture was neutralized with 5 % $HCl_{(aq)}$, and extracted with saturated brine and CH_2Cl_2. The collected organic layer was dried over $MgSO_4$, filtered and concentrated under reduced pressure. The crude product was purified by silica gel column chromatography with hexane/CH_2Cl_2 (10:1, v/v) and then hexane/CH_2Cl_2 (3:1, v/v) eluent to afford compound **8** as white solid (257 mg, 21%). Mp: >300 °C; ^{1}H NMR (400 MHz, $CDCl_3$): δ = 7.30-7.35 (m, 8H), 6.96-6.92 (m, 8H), 5.65 (s, 2H), 5.62 (s, 2H), 4.72 (s, 1H), 3.90 (t, *J* = 6.8 Hz, 2H), 2.01 (quin, *J* = 7.3 Hz, 2H), 1.68 (quin, *J* = 7.3 Hz, 2H), 1.54-1.39 (m, 8H), 0.96 (t, *J* = 6.6 Hz, 3H); ^{13}C{^{1}H} NMR (100 MHz, $CDCl_3$): δ = 145.3, 145.2, 144.2, 141.6, 136.3, 130.6, 125.1, 125.1, 123.5, 123.5, 76.3, 48.4, 47.6, 31.9, 30.5, 29.6, 29.4, 26.4, 22.7, 14.1; IR (KBr): 751, 1021, 1089, 1109, 1149, 1197, 1246, 1297, 1460, 1598, 2854, 2925, 3014, 3070, 3328 cm^{-1}; HRMS (ESI-TOF): m/z calculated for $C_{42}H_{39}O_2^+$ ([M+H]$^+$): 575.2945. Found: 575.2926.

Synthesis of Compound 9

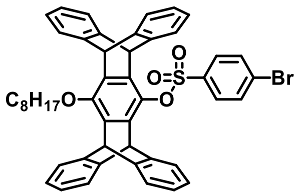

Compound **8** (400 mg, 0.70 mmol) in CH_2Cl_2 (40 mL) and triethylamine (4 mL) was stirred at room temperature for 30 minutes. 4-Bromobenzenesulfonyl chloride (213 mg, 0.84 mmol) in CH_2Cl_2 (10 mL) was then added dropwise to the mixture. The reaction was stirred at room temperature for 16 hours. The mixture was neutralized with 5% HCl(aq) and extracted with saturated brine and CH_2Cl_2. The collected organic layer was dried over $MgSO_4$, filtered, and concentrated under reduced pressure. The crude product was purified by silica gel column chromatography using hexane/ CH_2Cl_2 (10:1, v/v) followed by hexane/ CH_2Cl_2 (5:1, v/v) to afford compound **9** as a white solid (501 mg, 90%). Mp: 215 °C; ^{1}H NMR (400 MHz, CD_2Cl_2): δ = 8.10 (d, *J* = 8.7 Hz, 2H), 7.92 (d, *J* = 8.7 Hz, 2H), 7.35-7.30 (m, 4H), 7.26-7.21 (m, 4H), 6.97-6.94 (m, 8H), 5.69 (s, 2H), 5.61 (s, 2H), 3.96 (t, *J* = 6.7 Hz, 2H), 2.04 (quin, *J* = 7.1 Hz, 2H), 1.70 (quin, *J* = 7.8 Hz, 2H), 1.51-1.38 (m, 8H), 0.96 (t, *J* = 6.8 Hz, 3H); ^{13}C{^{1}H} NMR (100 MHz, CD_2Cl_2): δ = 148.9, 145.6, 145.2, 138.7, 138.1, 136.3, 136.0, 133.8, 130.5, 130.4, 125.9, 125.7, 124.8, 124.0, 76.9, 49.7, 48.8, 32.5, 31.1, 30.2, 30.0, 27.0, 23.3, 14.5; IR (KBr): 702, 737, 819, 861, 986, 1011, 1069, 1111, 1182, 1243, 1303, 1380, 1460, 1575, 2855, 2928, 3022, 3068 cm^{-1}; HRMS (ESI-TOF): m/z calculated for $C_{48}H_{42}BrO_4S^+$ ([M+H]$^+$): 793.1982. Found: 793.1971.

Synthesis of Compound 10

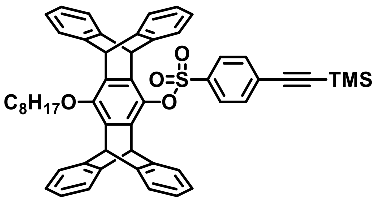

A mixture of compound **9** (1 g, 1.26 mmol), $Pd(PPh_3)_2Cl_2$ (44.2 mg, 0.06 mmol), CuI (12 mg, 0.06 mmol), and trimethylsilylacetylene (0.2 mL, 1.51 mmol) in degassed toluene (8 mL) and triethylamine (40 mL) was refluxed at 90 °C for 18 hours under a nitrogen atmosphere. Toluene and triethylamine were purged for 20 minutes prior to the reaction. After cooling to room temperature, the mixture was extracted with saturated brine and CH_2Cl_2. The collected organic layer was dried over $MgSO_4$, filtered, and concentrated under reduced pressure. The crude product was purified by silica gel column chromatography using hexane/ CH_2Cl_2 (10:1, v/v) followed by hexane/ CH_2Cl_2 (5:1, v/v) to afford compound **10** as a white solid (884 mg, 87%). Mp: 136 °C; ^{1}H NMR (400 MHz, CD_2Cl_2): δ = 8.18 (d, *J* = 8.4 Hz, 2H), 7.82 (d, *J* = 8.5 Hz, 2H), 7.34-7.32 (m, 4H), 7.25-7.23 (m, 4H), 6.97-6.92 (m, 8H), 5.70 (s, 2H), 5.62 (s, 2H), 3.97 (t, *J* = 6.7 Hz, 2H), 2.05 (quin, *J* = 7.8 Hz, 2H), 1.71 (quin, *J* = 7.6 Hz, 2H), 1.51-1.40 (m, 8H), 0.96 (t, *J* = 6.9 Hz, 3H), 0.33 (s, 9H); ^{13}C{^{1}H} NMR (100 MHz, CD_2Cl_2): δ = 148.9, 145.6, 145.3, 138.6, 138.2, 136.5, 136.1, 133.6, 130.5, 128.9, 125.9, 125.8, 124.8, 124.0, 103.3, 100.7, 76.9, 49.7, 48.8, 32.6, 31.1, 30.2, 30.0, 27.0, 23.4, 14.6, 0.0; IR (KBr): 755, 810, 862, 986, 1091, 1108, 1176, 1190, 1241, 1302, 1380, 1459, 1589, 2170, 2859, 2927, 3068 cm^{-1}; HRMS (ESI-TOF): m/z calculated for $C_{53}H_{51}O_4SSi^+$ ($[M+H]^+$): 811.3272. Found: 811.3231.

Synthesis of Compound 11

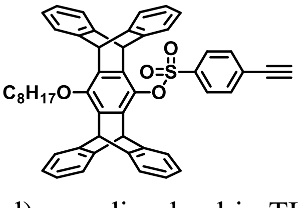

Compound **10** (300 mg, 0.37 mmol) was dissolved in THF (20 mL) and methanol (5 mL) mixed solution. Subsequently, K_2CO_3 (61 mg, 0.44 mmol) was added, and the mixture was stirred at room temperature for l h. After removal of the volatile solvent under reduced pressure, the residual was extracted with saturated brine and CH_2Cl_2. The collected organic layer was dried over $MgSO_4$, filtered and concentrated under reduced pressure. The crude product was purified by silica gel column chromatography with hexane/CH_2Cl_2 (10:1, v/v) and then hexane/CH_2Cl_2 (3:1, v/v) eluent to afford compound **11** as white solid (259 mg, 95%). Mp: 204 °C; ^{1}H NMR (400 MHz, CD_2Cl_2):

δ = 8.20 (d, *J* = 8.6 Hz, 2H), 7.88 (d, *J* = 8.5 Hz, 2H), 7.34-7.32 (m, 4H), 7.25-7.23 (m, 4H), 6.97-6.92 (m, 8H), 5.70 (s, 2H), 5.63 (s, 2H), 3.97 (t, *J* = 6.7 Hz, 2H), 3.49 (s, 1H), 2.04 (quin, *J* = 6.9 Hz, 2H), 1.71 (quin, *J* = 6.7 Hz, 2H), 1.51-1.41 (m, 8H), 0.97 (t, *J* = 7.0 Hz, 3H); $^{13}C\{^1H\}$ NMR (100 MHz, CD_2Cl_2): δ = 148.9, 145.6, 145.2, 138.6, 138.2, 137.1, 136.0, 133.9, 129.4, 128.9, 125.9, 125.7, 124.8, 124.0, 82.4, 82.2, 76.9, 49.7, 48.8, 32.6, 31.1, 30.2, 30.0, 27.0, 23.3, 14.5; IR (KBr): 756, 818, 859, 986, 1092, 1109, 1174, 1188, 1240, 1301, 1379, 1459, 1589, 2859, 2927, 3286 cm^{-1} (Figure S29); HRMS (ESI-TOF): m/z calculated for $C_{50}H_{43}O_4S^+$ ($[M+H]^+$): 739.2877. Found: 739.2842.

Synthesis of Compound 3

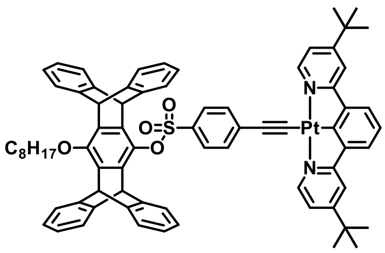

A mixture of compound **11** (100 mg, 0.14 mmol), compound **7**[S6] (85 mg, 0.15 mmol) and CuI (1.3 mg, 7 μmol) in degassed CH_2Cl_2 (5 mL) and diisopropylamine (5 mL) was stirred at 60 °C for 48h under nitrogen atmosphere. After cooling to room temperature, the mixture was extracted with water and CH_2Cl_2. The collected organic layer was dried over $MgSO_4$, filtered and concentrated under reduced pressure. The crude product was purified by Al_2O_3 column chromatography with hexane/CH_2Cl_2/ethyl acetate (30:10:1, v/v/v) to afford compound **3** as yellow solid (66 mg, 38%). Mp: 238 °C (decomposed); ^1H NMR (400 MHz, CD_2Cl_2): δ = 9.33-9.23 (m, 2H), 8.15 (d, *J* = 8.6 Hz, 2H), 7.86 (d, *J* = 8.6 Hz, 2H), 7.74 (d, *J* = 2.1 Hz, 2H), 7.61 (d, *J* = 7.7 Hz, 2H), 7.35-7.33 (m, 4H), 7.31-7.29 (m, 4H), 7.29-7.26 (m, 3H), 6.99-6.94 (m, 8H), 5.70 (d, *J* = 5.2 Hz, 4H), 3.98 (t, *J* = 6.8 Hz, 2H), 2.06 (quin, *J* = 7.9 Hz, 2H), 1.72 (quin, *J* = 7.7 Hz, 2H), 1.53-1.42 (m, 26H), 0.97 (t, *J* = 7.1 Hz, 3H); $^{13}C\{^1H\}$ NMR (100 MHz, CD_2Cl_2): δ = 178.4, 169.7, 164.5, 155.0, 148.7, 148.1, 145.6, 145.4, 144.2, 138.4, 138.3, 136.5, 136.4, 132.9, 132.4, 128.7, 125.8, 125.7, 124.9, 124.2, 123.9, 123.8, 121.6, 117.4, 110.9, 76.9, 49.7, 48.8, 36.1, 32.6, 31.1, 30.6, 30.2, 30.0, 27.0, 23.3, 14.5; IR (KBr): 755, 820, 986, 1092, 1170, 1189, 1240, 1300, 1379, 1458, 1483, 1583, 1616, 2078, 2926, 2958 cm^{-1}; HRMS (ESI-TOF): m/z calculated for $C_{74}H_{69}N_2O_4PtS^+$ ($[M+H]^+$): 1276.4626. Found: 1276.4570.

Table S1. X-ray crystallographic data of **2**.

Empirical formula	C98 H82 N4 O6 Pt2 S2	
Formula weight	1865.97	
Crystal system	Triclinic	
Space group	P-1	
Unit cell dimensions	a = 13.4440(9) Å	α= 62.381(8)°.
	b = 13.7731(12) Å	β= 84.897(6)°.
	c = 14.6877(11) Å	γ= 85.322(7)°.
Volume	2397.7(4) Å^3	
Z	1	
F(000)	934	
Density (calculated)	1.292 Mg/m^3	
Wavelength	0.71073 Å	
Cell parameters reflections used	5797	
Theta range for Cell parameters	3.6430 to 29.4110°.	
Absorption coefficient	3.008 mm^{-1}	
Temperature	100(2) K	
Crystal size	0.200 x 0.150 x 0.150 mm^3	
	Data collection	
Diffractometer	Xcalibur, Atlas, Gemini	
Absorption correction	Semi-empirical from equivalents	
Max. and min. transmission	1.00000 and 0.44965	
No. of measured reflections	18517	
No. of independent reflections	10909 [R(int) = 0.0507]	
No. of observed [I>2_igma(I)]	8336	
Completeness to theta = 25.242°	99.7 %	
Theta range for data collection	3.166 to 27.500°.	
	Refinement	
Final R indices [I>2sigma(I)]	R1 = 0.0504, wR2 = 0.0975	
R indices (all data)	R1 = 0.0736, wR2 = 0.1149	
Goodness-of-fit on F^2	1.004	
Largest diff. peak and hole	1.682 and -1.767 e.Å^{-3}	

Table S2. TDDFT-derived energy, oscillator strength (*f*), and natural transition orbital (NTO) pairs with transition character analysis for **3** and the folded form of **2**.

<table>
<tr><th>Compd.</th><th>λmax (nm)</th><th>f</th><th>Simulated absoption spectra</th><th>Character b,c</th></tr>
<tr><td rowspan="3">3</td><td>327 (S1)</td><td>0.1289</td><td rowspan="2">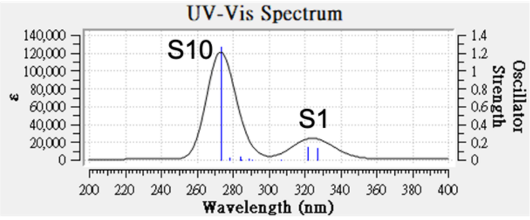
</td><td>ILCT
LMCT</td></tr>
<tr><td>273 (S10)</td><td>1.2700</td><td>LE</td></tr>
<tr><td>519 (T1)</td><td>0</td><td>--</td><td>LC</td></tr>
<tr><td colspan="5">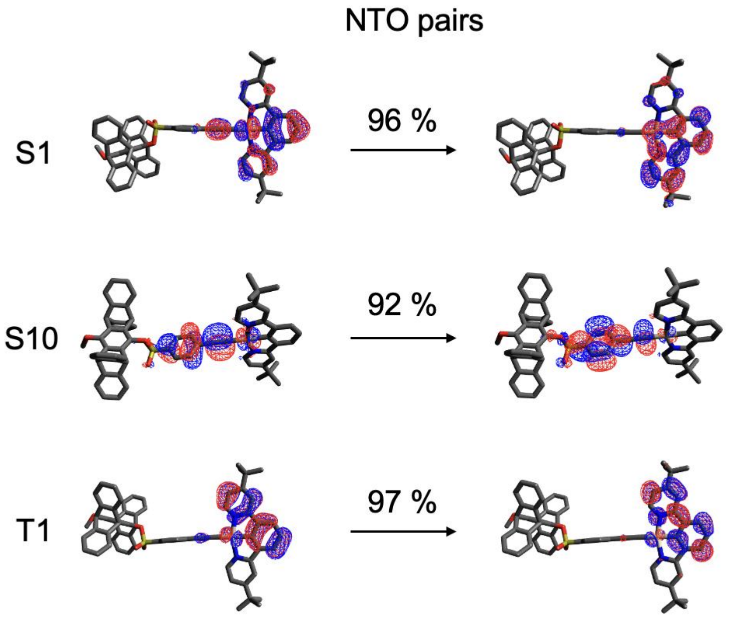
</td></tr>
<tr><td rowspan="3">2</td><td>329 (S1)</td><td>0.1062</td><td rowspan="2">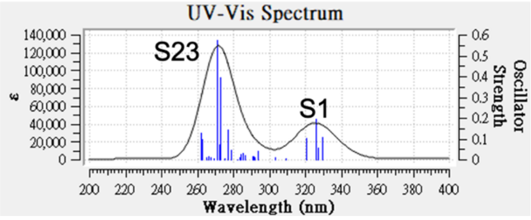
</td><td>ILCT
LMCT</td></tr>
<tr><td>271 (S23)</td><td>0.5729</td><td>LE
LMCT
ILCT</td></tr>
<tr><td>846 (T1)</td><td>0</td><td>--</td><td>ILCT</td></tr>
</table>

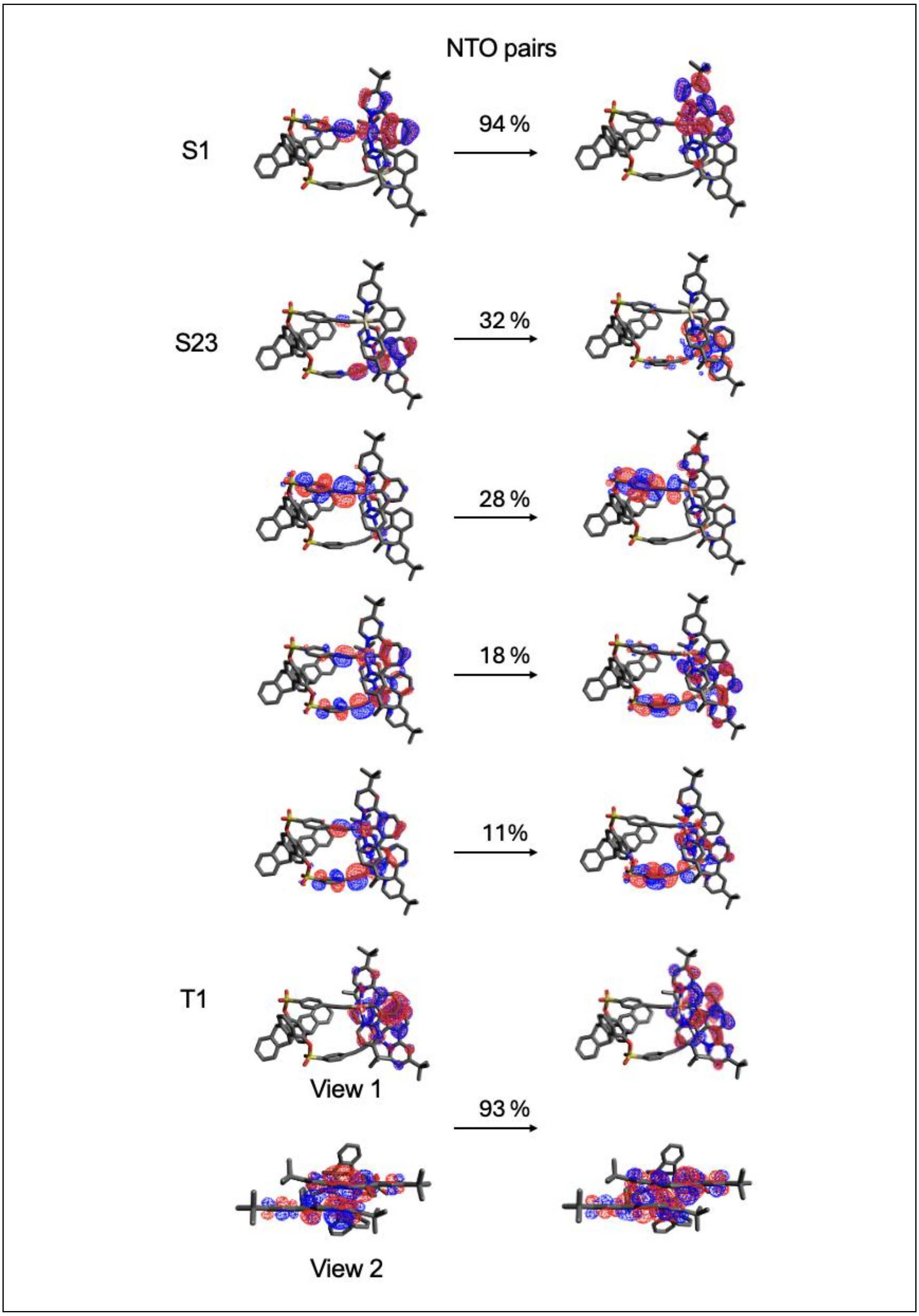

[a] Percentage weights of hole – electron.[b] LE denotes the transition of phenylacetylide: i.e., the phenylacetylide [ππ*] state, LC denotes the transition of NCN-ligand, and LMCT denotes the ligand-to-metal charge-transfer [π(NCN-ligand) → Pt] or [π(phenylacetylide) → Pt] transition. ILCT denotes the intra-ligand charge transfer. LLCT denotes the ligand-to-ligand charge transfer [c] Only CI > 10 % are listed.

Table S3. TDDFT-derived energy, oscillator strength (*f*), and natural transition orbital (NTO) pairs with transition character analysis for the extended form of **2** in crystal structure.

**Compd.**	**λ_{max} (nm)**	***f***	NTO pair [a]	**Character** [b,c]
**2**	344 (S_1)	0.2003	For clarity, only the key parts are shown below.	LC<br>ILCT
	399 (T_1)	0	For clarity, only the key parts are shown below.	LE<br>LMCT<br>LC

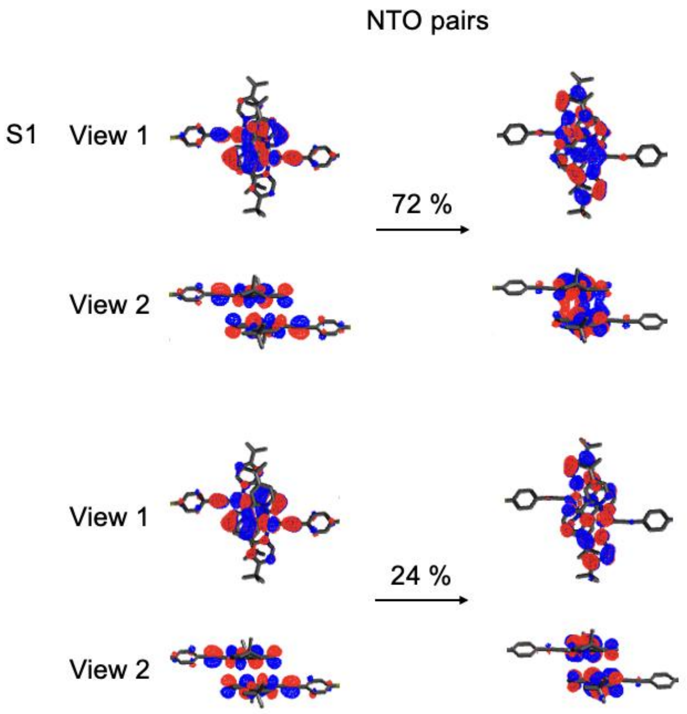

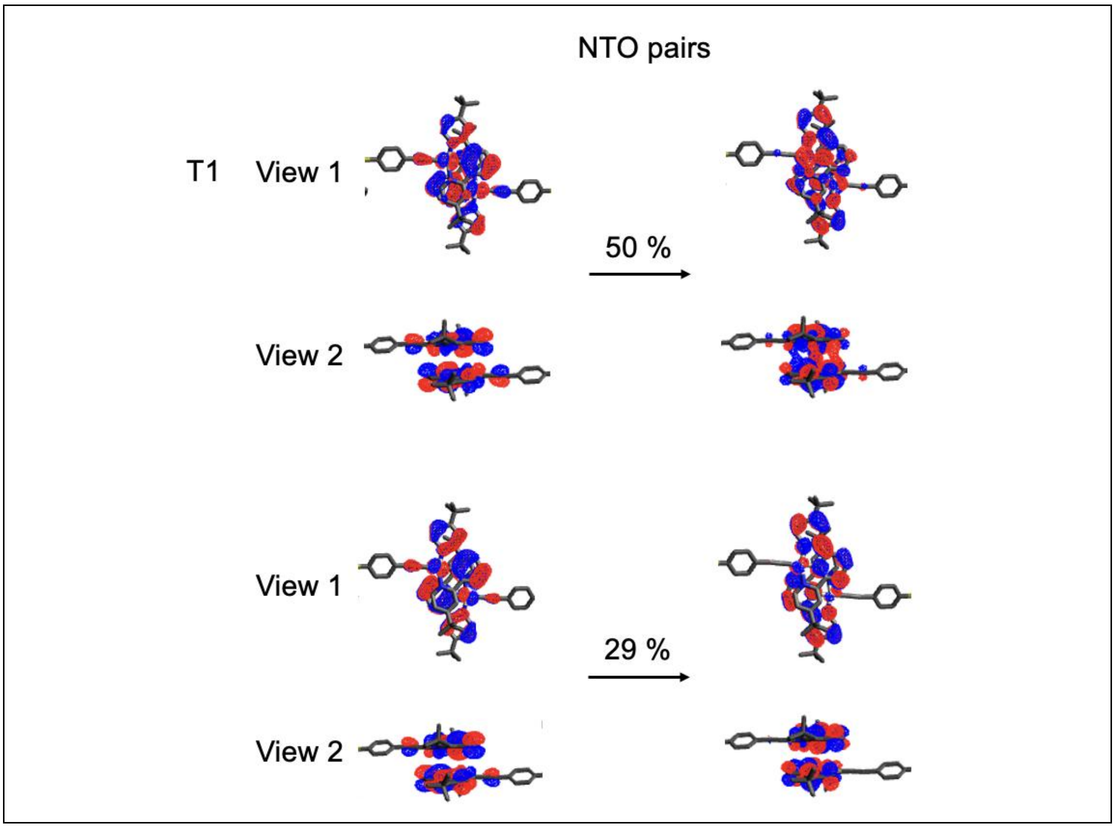

[a] Percentage weights of hole – electron.[b] LE denotes the transition of phenylacetylide: i.e., the phenylacetylide [ππ*] state, LC denotes the transition of NCN-ligand, and LMCT denotes the ligand-to-metal charge-transfer [π(NCN-ligand) → Pt] or [π(phenylacetylide) → Pt] transition, ILCT denotes the intra-ligand charge transfer. [c] Only CI > 10 % are listed.

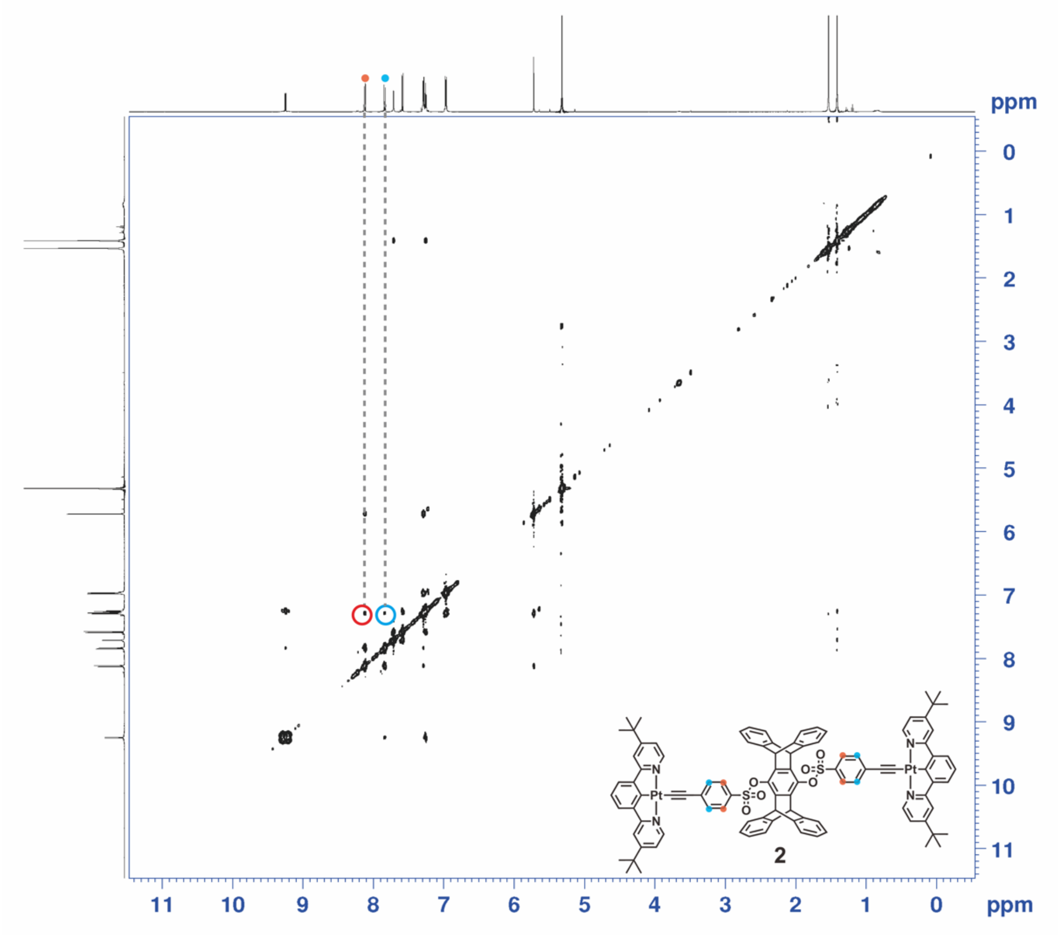

Figure S1. 500 MHz ^{1}H–^{1}H ROESY spectrum of compound **2** in CD_2Cl_2.

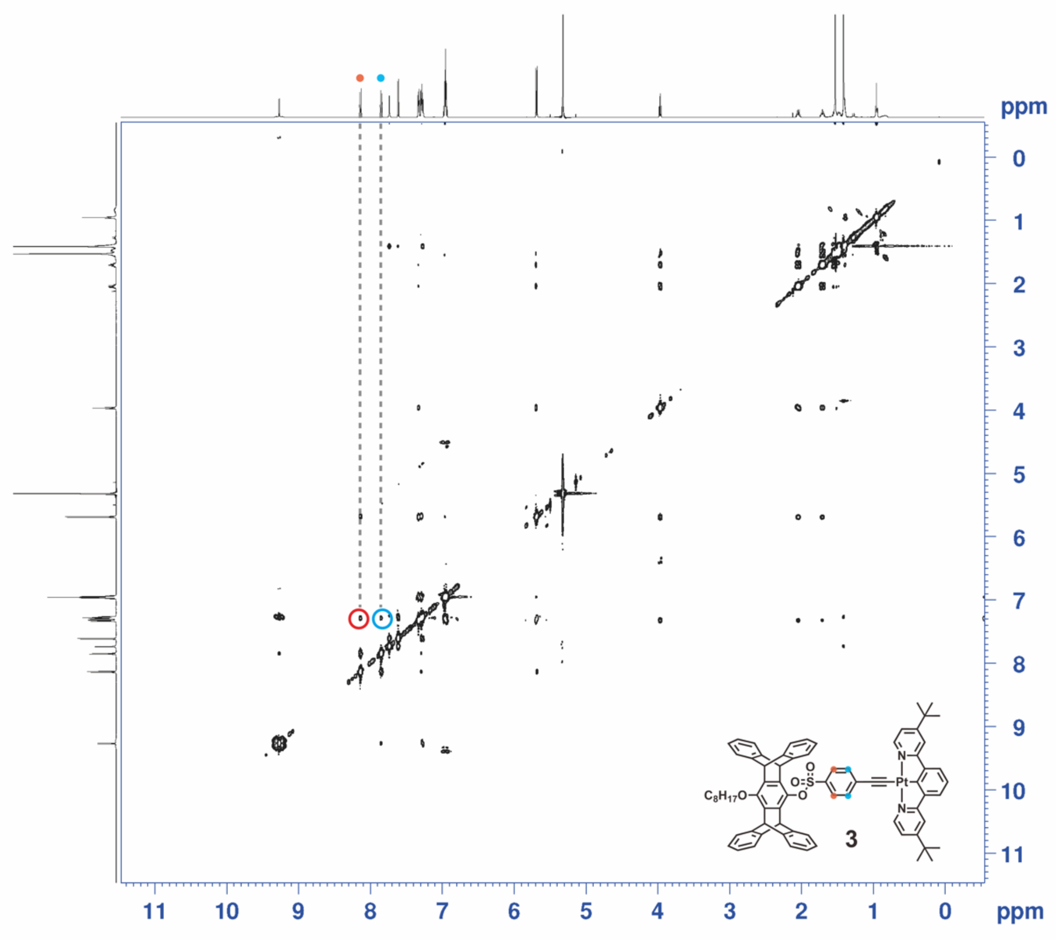

Figure S2. 500 MHz ^{1}H–^{1}H ROESY spectrum of compound **3** in CD_2Cl_2.

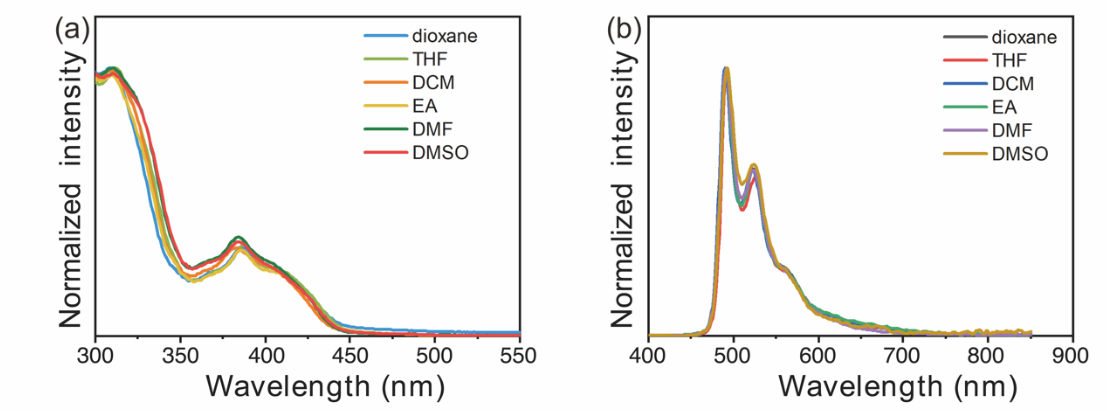

Figure S3. (a) Absorption and (b) emission spectra of **3** in solvents of different polarity.

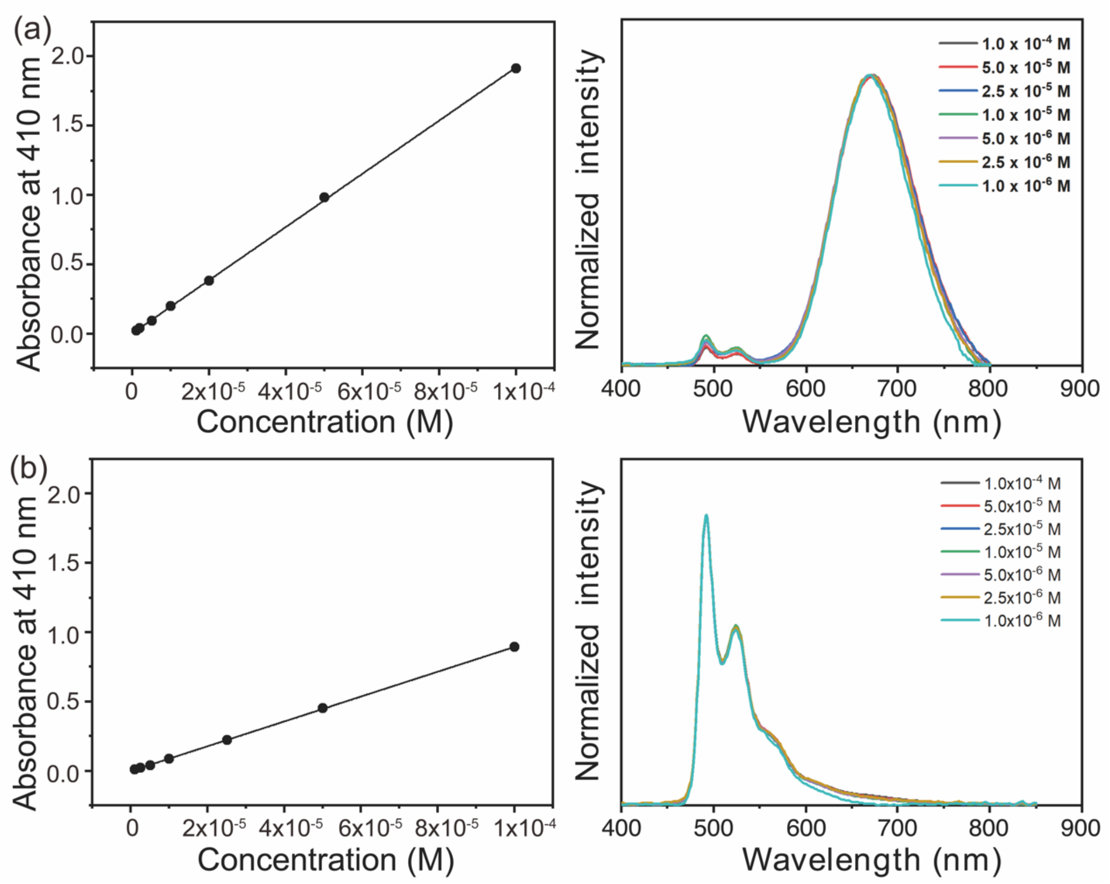

Figure S4. Absorbance at 410 nm (left) and emission profile (right) of (a) complex **2** and (b) complex **3** at concentrations of 1−100 µM in THF.

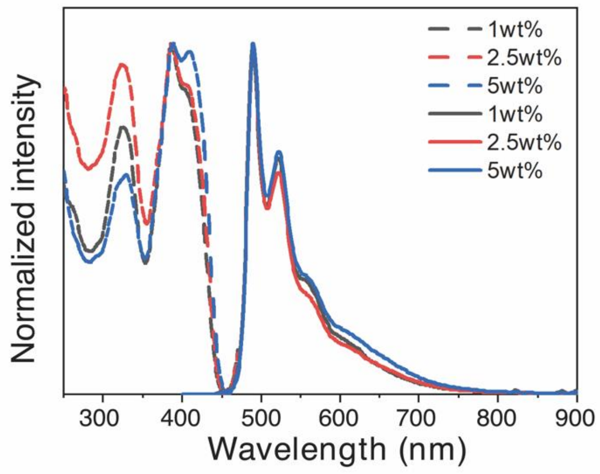

Figure S5. Excitation spectra (dashed lines) and emission spectra (solid lines) of compound **2** in PMMA films at 1–5 wt% loadings. Excitation spectra were monitored at 490 nm.

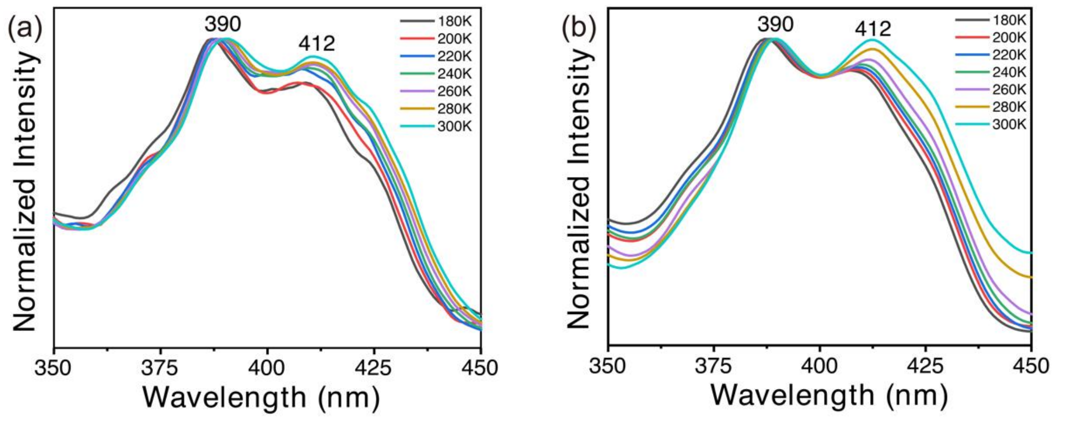

Figure S6. Excitation spectra of **2** at different temperatures in 2-methyltetrahydrofuran, monitored at (a) 490 nm and (b) 662 nm.

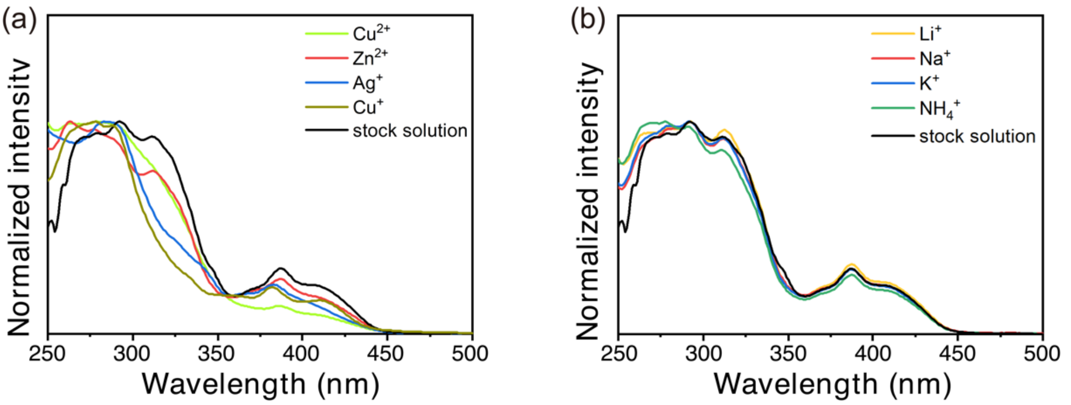

Figure S7. Absorption spectra of **2** with the addition of (a) Cu^{2+}, Zn^{2+}, Ag^{+} and Cu^{+}, and (b) Li^{+}, Na^{+}, K^{+}, and NH_4^{+}, compared to the stock solution.

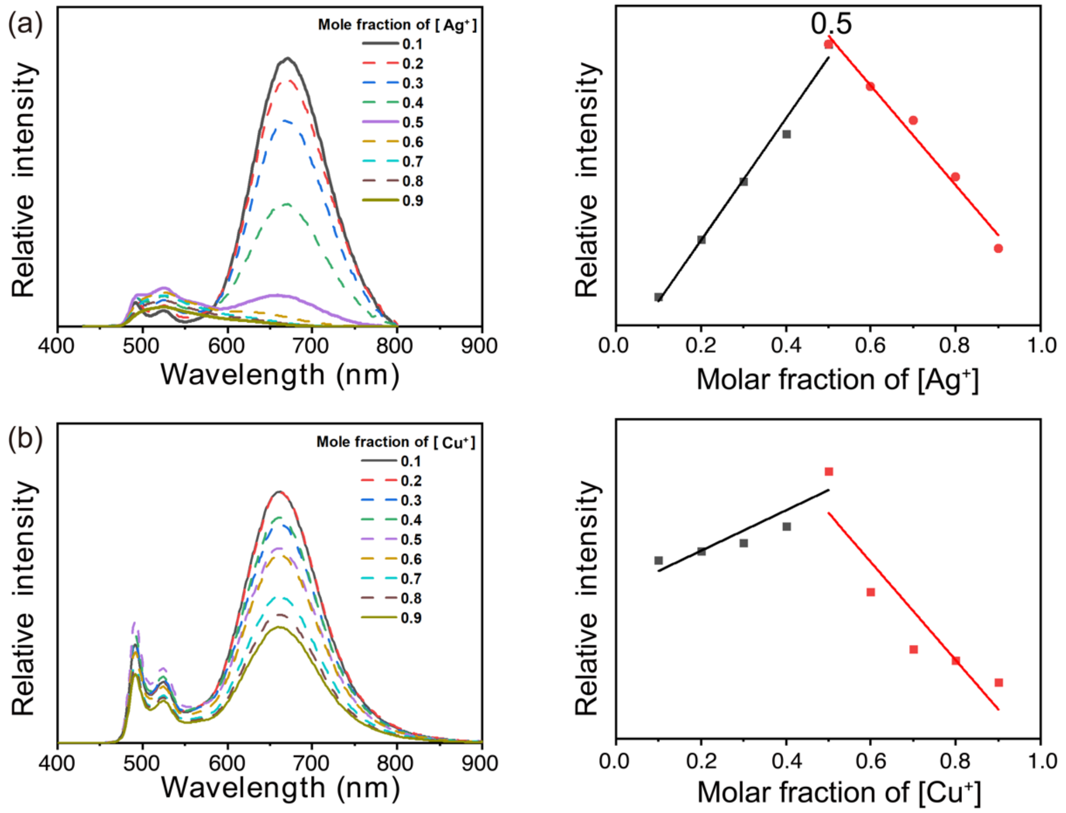

Figure S8. Emission spectra of compound **2** in degassed THF with varying equivalents of (a) Ag^+ and (b) Cu^+ ions (left), and corresponding Job plots of emission intensity at 520 nm (right).

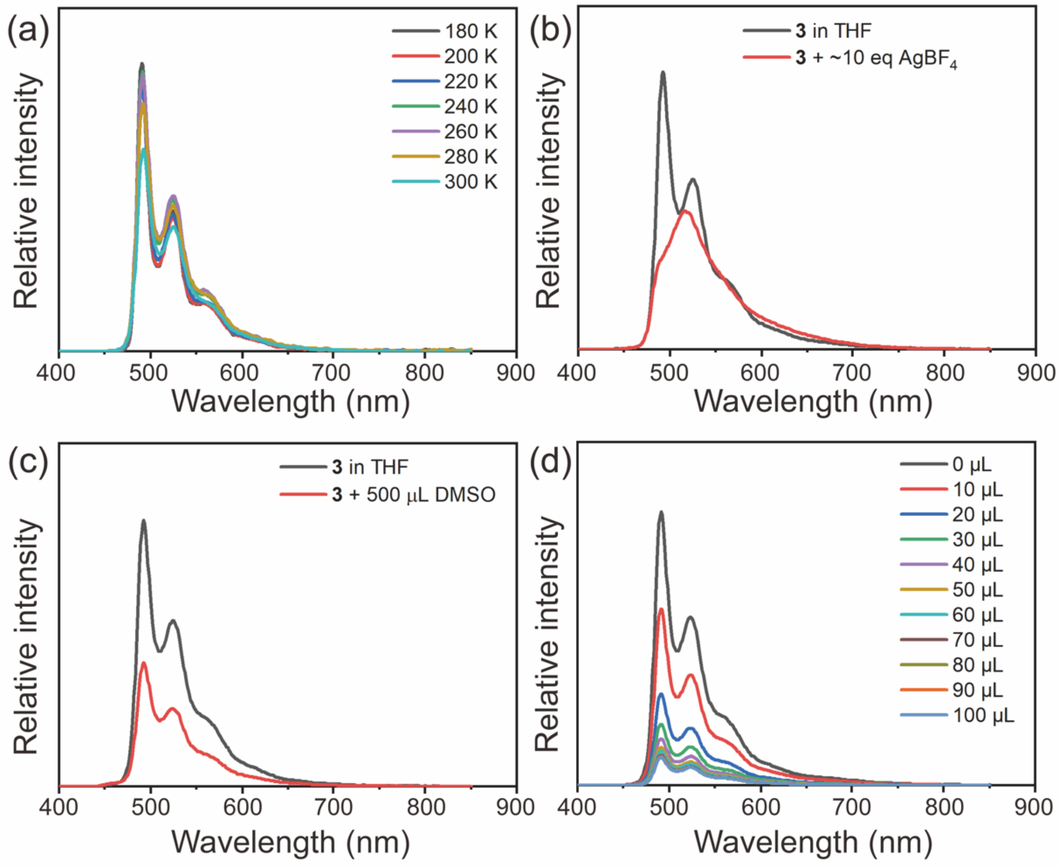

Figure S9. Emission spectra for the stimuli-responsive properties of **3** under various conditions: (a) temperature variation, (b) addition of silver ions, (c) addition of DMSO, and (d) exposure to oxygen.

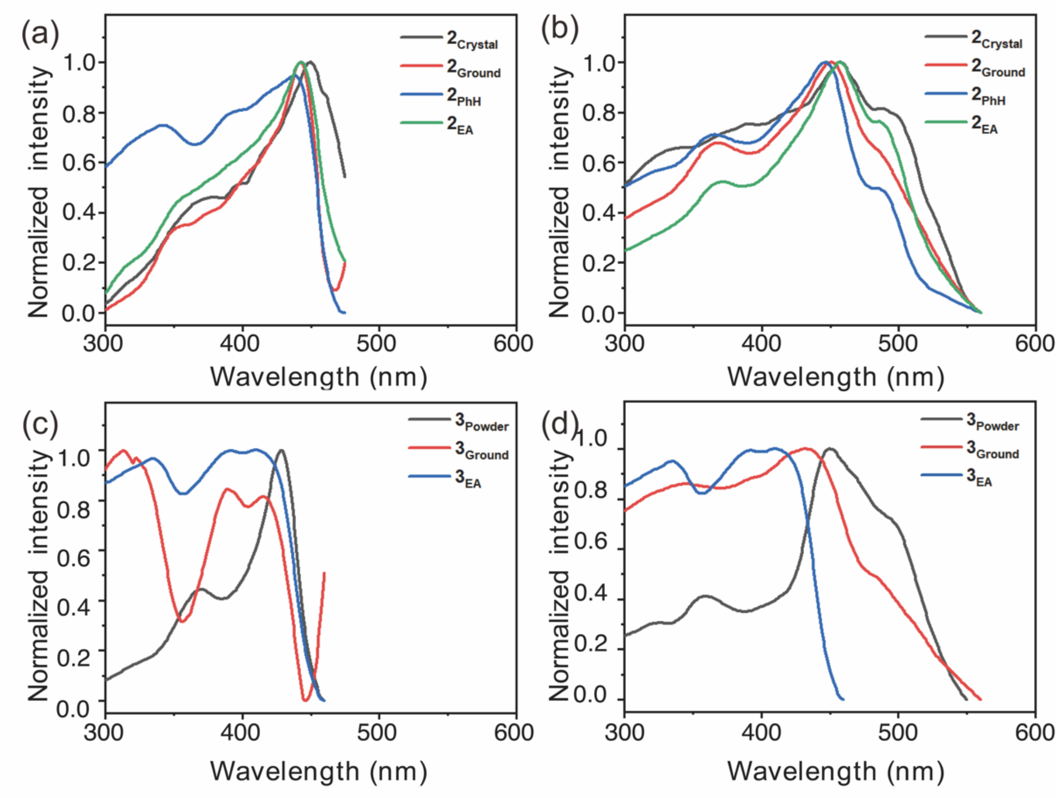

Figure S10. Excitation spectra of compound **2** monitored at (a) 490 nm and (b) 663 nm, and compound **3** monitored at (c) 489 nm and (d) 621 nm.

Table S4. Multiple luminescence lifetimes of **2** and **3** in the solid state under different external stimuli, measured using a 405 nm pulsed laser excitation source.

Compound	λ_p (nm)	τ_1 (μs)	τ_2 (μs)	τ_3 (μs)	τ_{ave} (μs) [a]
**2**$_{\mathbf{Crystal}}$	505	0.06 (0.23)	0.28 (0.48)	0.62 (0.25)	0.32
	653	0.07 (-0.74)	0.66 (0.33)	1.20 (0.26)	0.90
**2**$_{\mathbf{Gound}}$	500	0.02 (0.85)	0.16 (0.36)	0.47(0.26)	0.13
	664	0.002 (-0.96)	0.44 (0.31)	1.03 (0.31)	0.74
**2**$_{\mathbf{PhH}}$	502	0.04 (0.42)	0.16 (0.48)	0.42 (0.10)	0.14
	658	0.12 (-0.57)	0.95 (0.64)	1.51 (0.25)	1.11
**2**$_{\mathbf{EA}}$	502	0.03 (1.37)	0.11 (0.60)	0.30 (0.06)	0.06
	660	0.04 (-0.19)	0.43(0.42)	1.01 (0.36)	0.70
**3**$_{\mathbf{Powder}}$	489	0.33(0.27)	1.35 (0.42)	----	0.95
	621	0.78 (0.30)	1.90 (0.45)	----	1.45

**3_{Gound}**	496	0.04 (0.48)	0.19 (0.17)	1.38 (0.04)	0.15
	660	0.04 (-0.26)	0.73 (0.58)	1.54 (0.29)	1.00
**3_{EA}**	491	0.35 (0.23)	1.03 (0.32)	--	0.75
	650	0.12 (-0.21)	1.38 (0.64)	--	1.38

[a] The τ_{ave} refer to average lifetime, and it was calculated using $\frac{\sum_i^n A_i\tau_i}{\sum_i^n A_i}$, where A and τ are the amplitude and lifetime of each component, respectively. Negative amplitude values were excluded from the calculation.

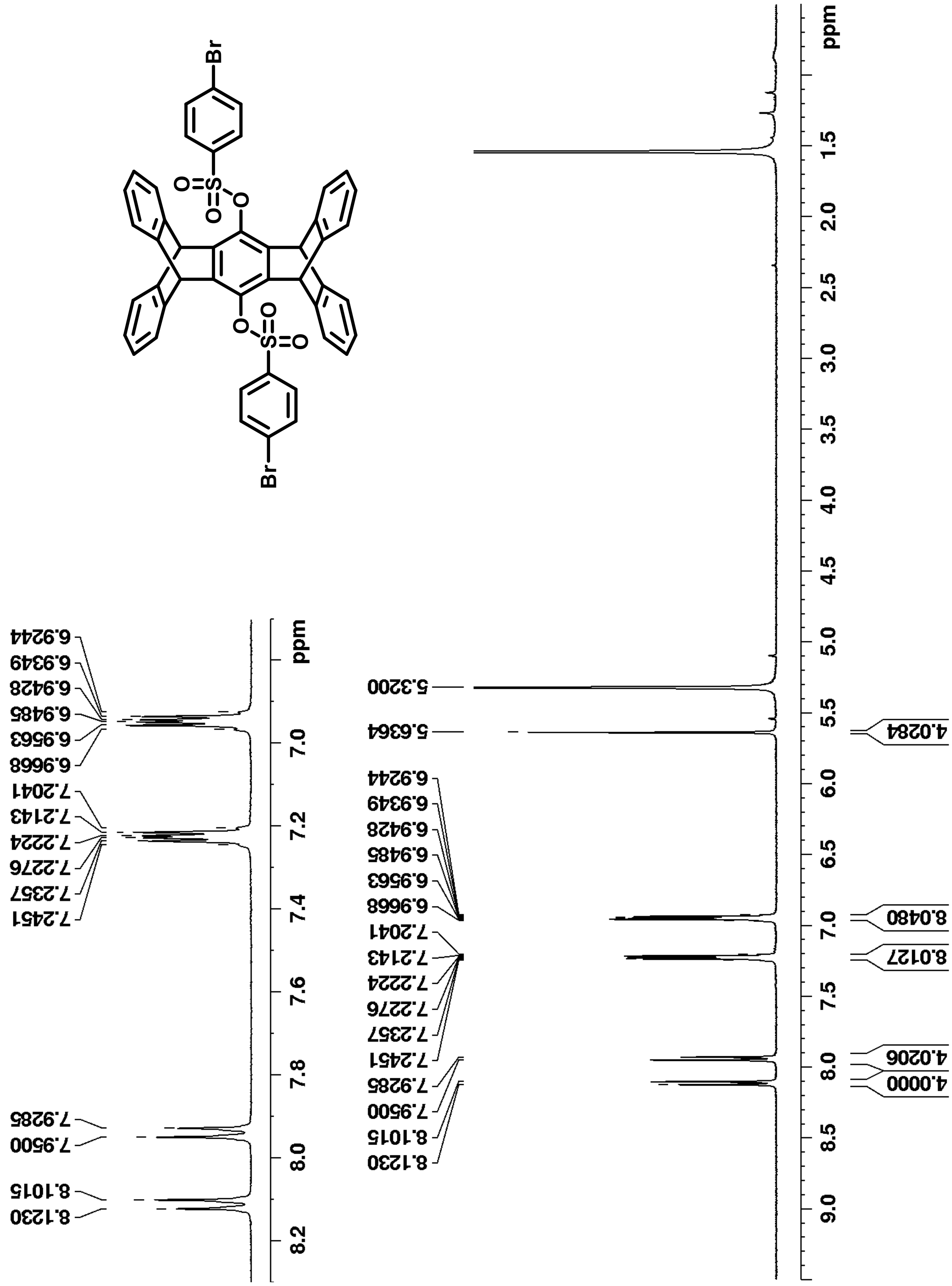

Figure S11. ^{1}H-NMR spectrum of compound **4** (400 MHz, CD_2Cl_2)

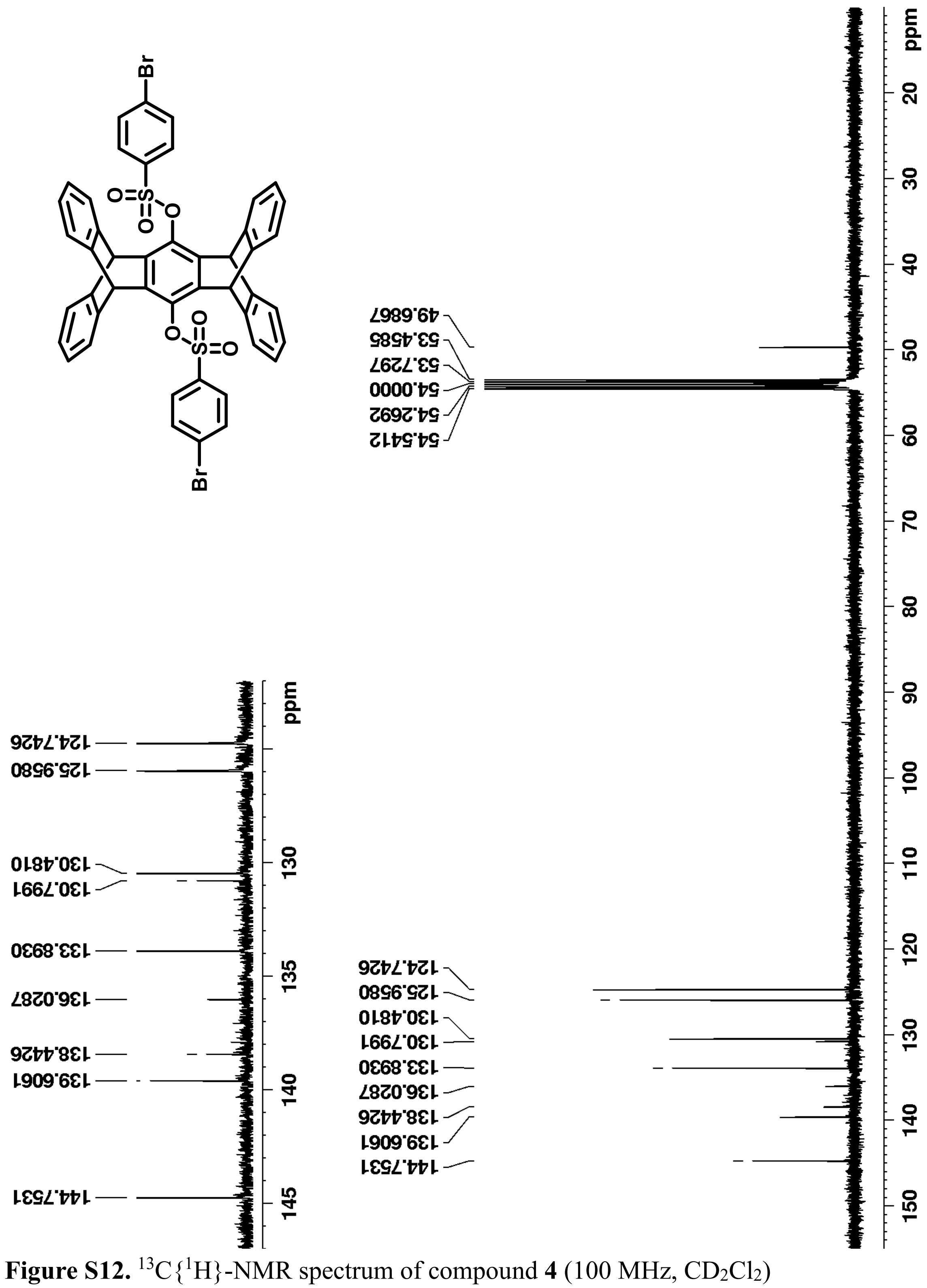

Figure S12. $^{13}C\{^{1}H\}$-NMR spectrum of compound **4** (100 MHz, CD_2Cl_2)

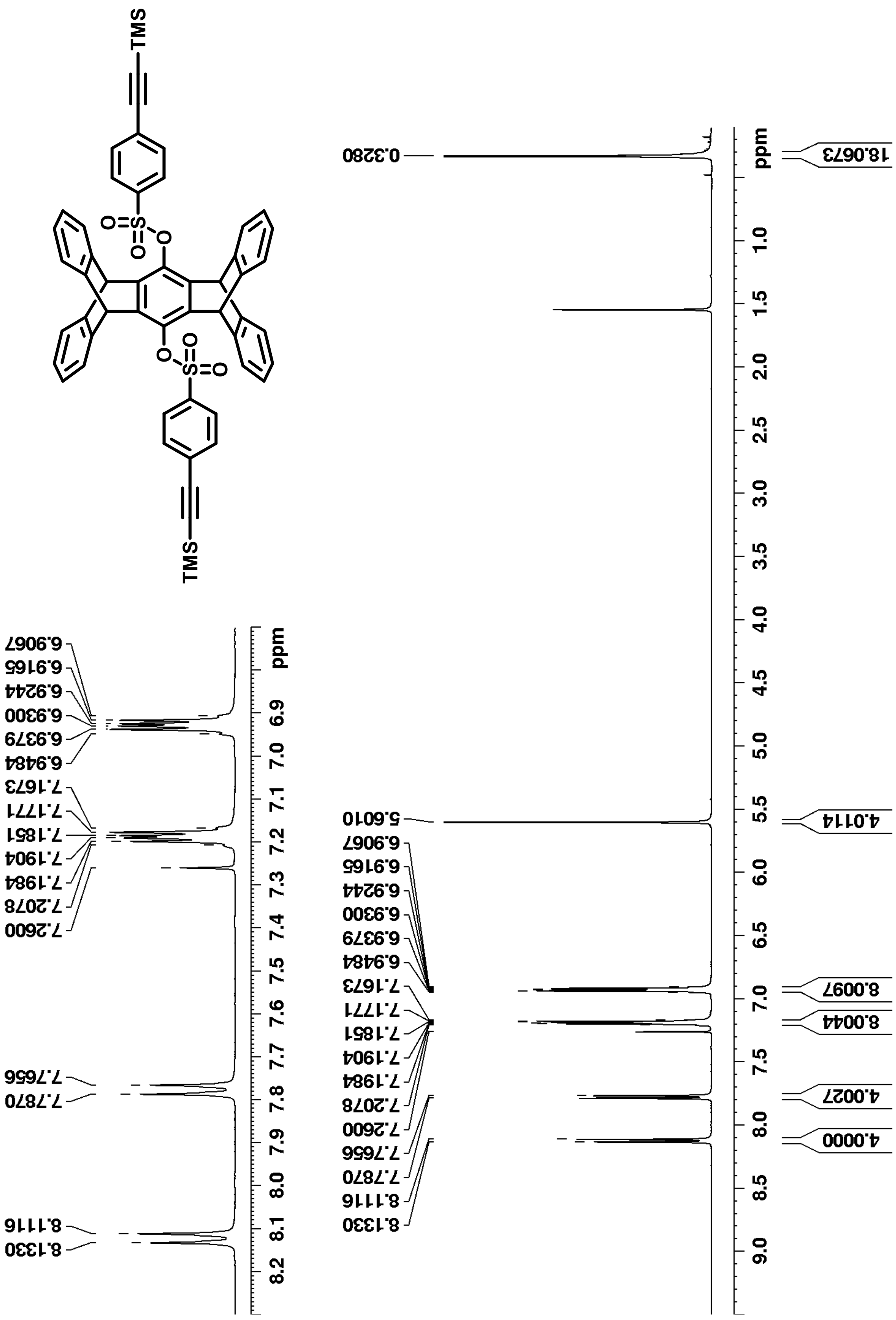

Figure S13. ^{1}H-NMR spectrum of compound **5** (400 MHz, $CDCl_3$)

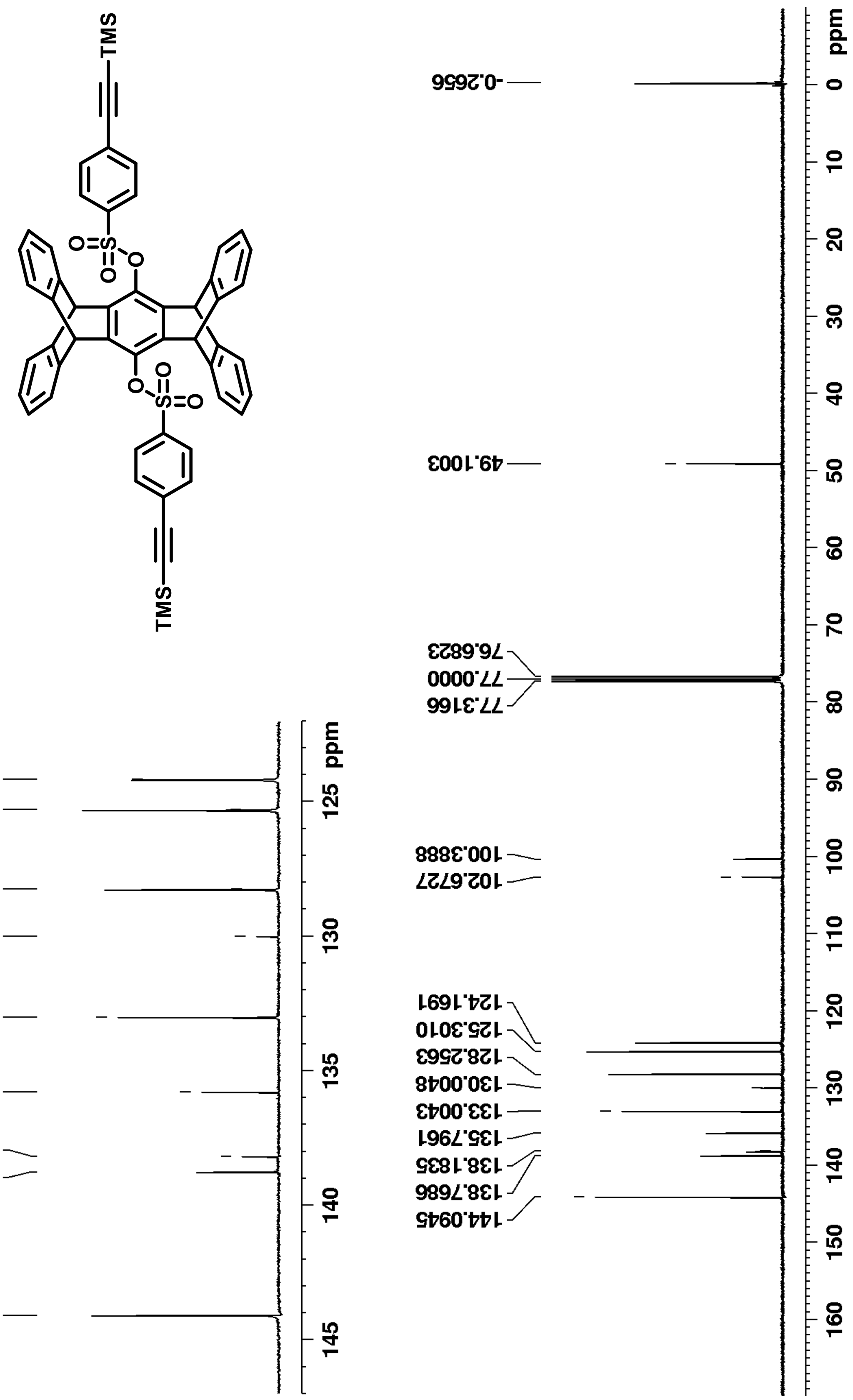

Figure S14. $^{13}C\{^{1}H\}$-NMR spectrum of compound **5** (100 MHz, $CDCl_3$)

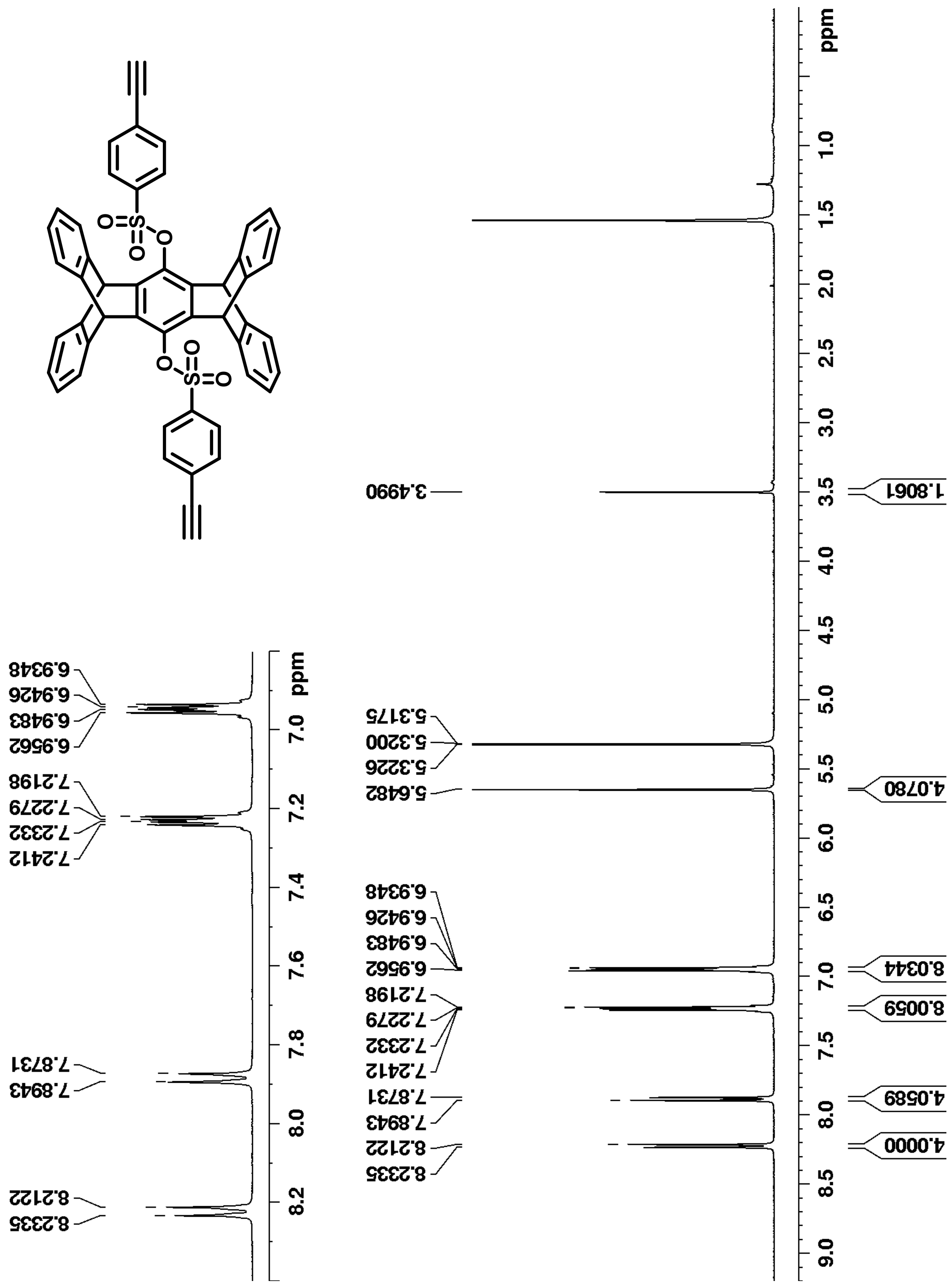

Figure S15. ^{1}H-NMR spectrum of compound **6** (400 MHz, CD_2Cl_2)

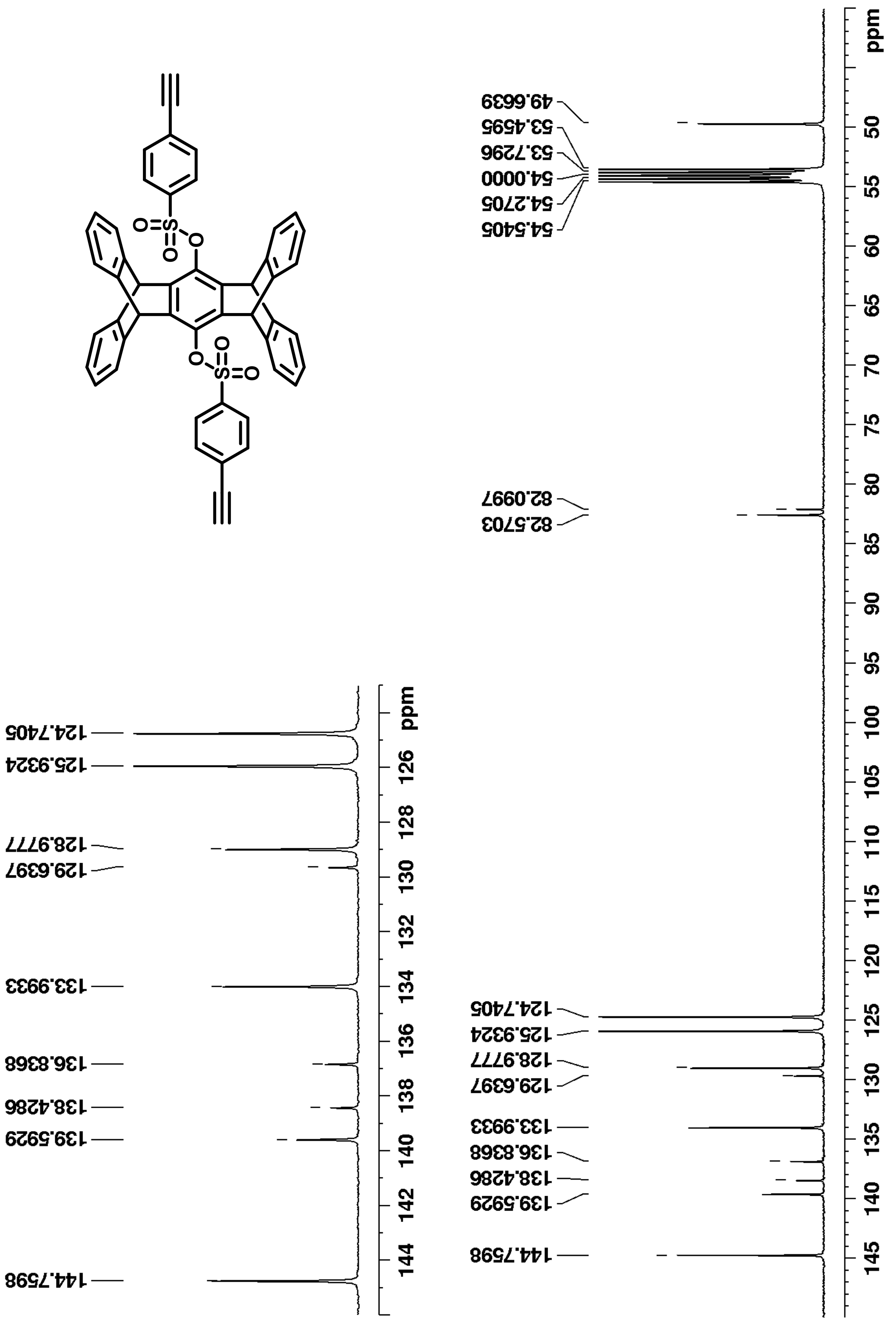

Figure S16. $^{13}C\{^{1}H\}$-NMR spectrum of compound **6** (100 MHz, CD_2Cl_2)

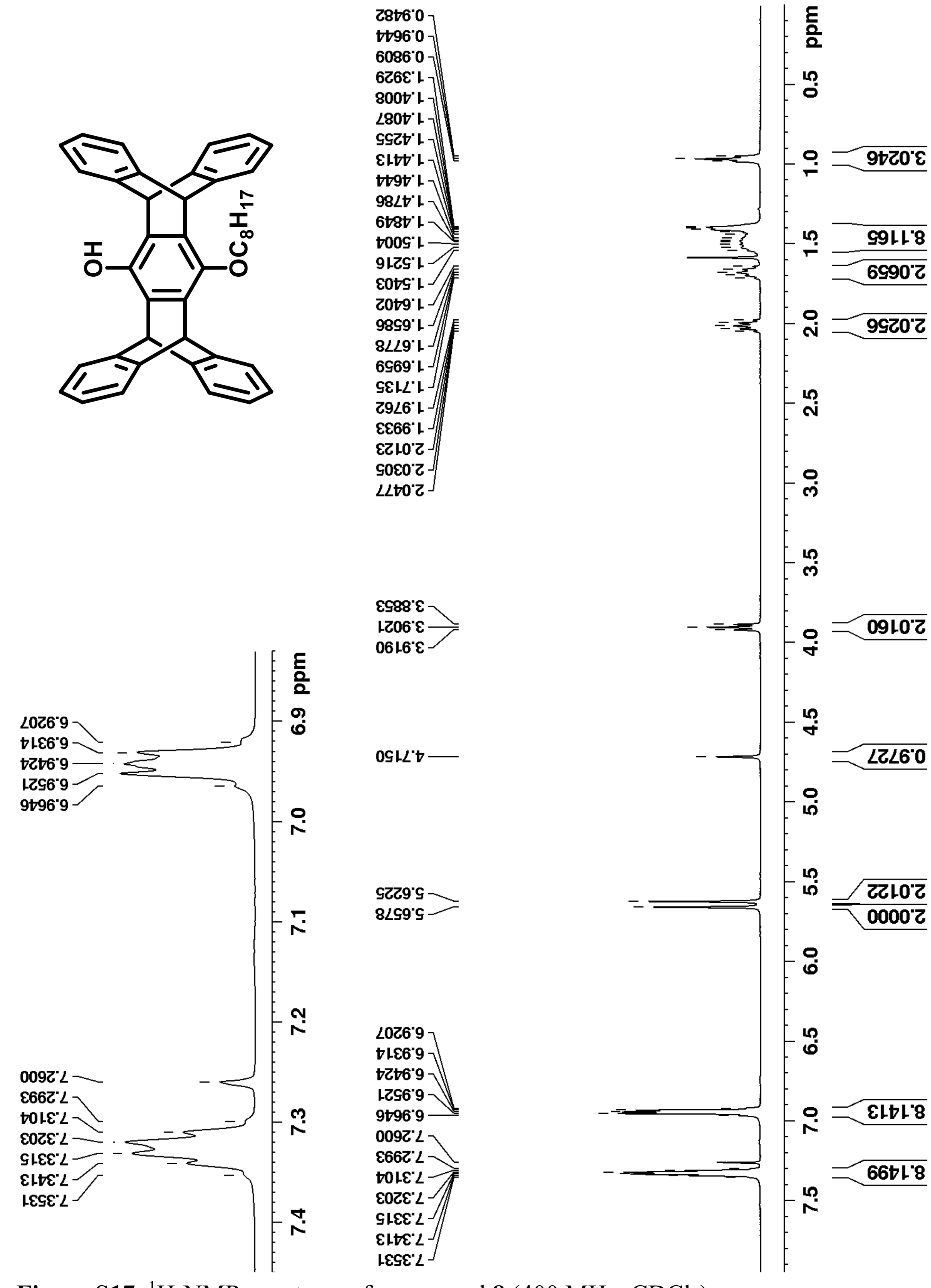

Figure S17. ^{1}H-NMR spectrum of compound **8** (400 MHz, $CDCl_3$)

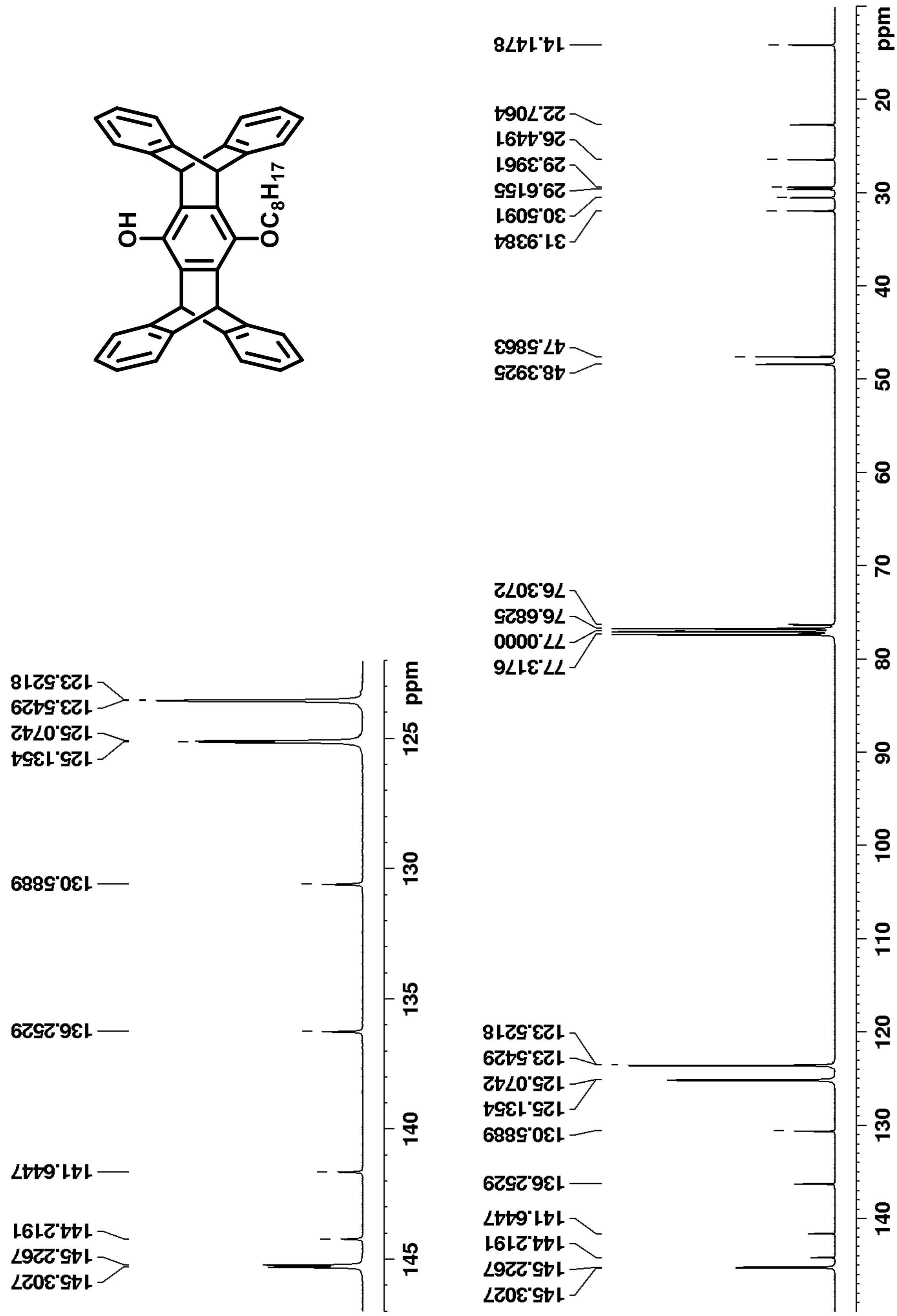

Figure S18. $^{13}C\{^1H\}$-NMR spectrum of compound **8** (100 MHz, $CDCl_3$)

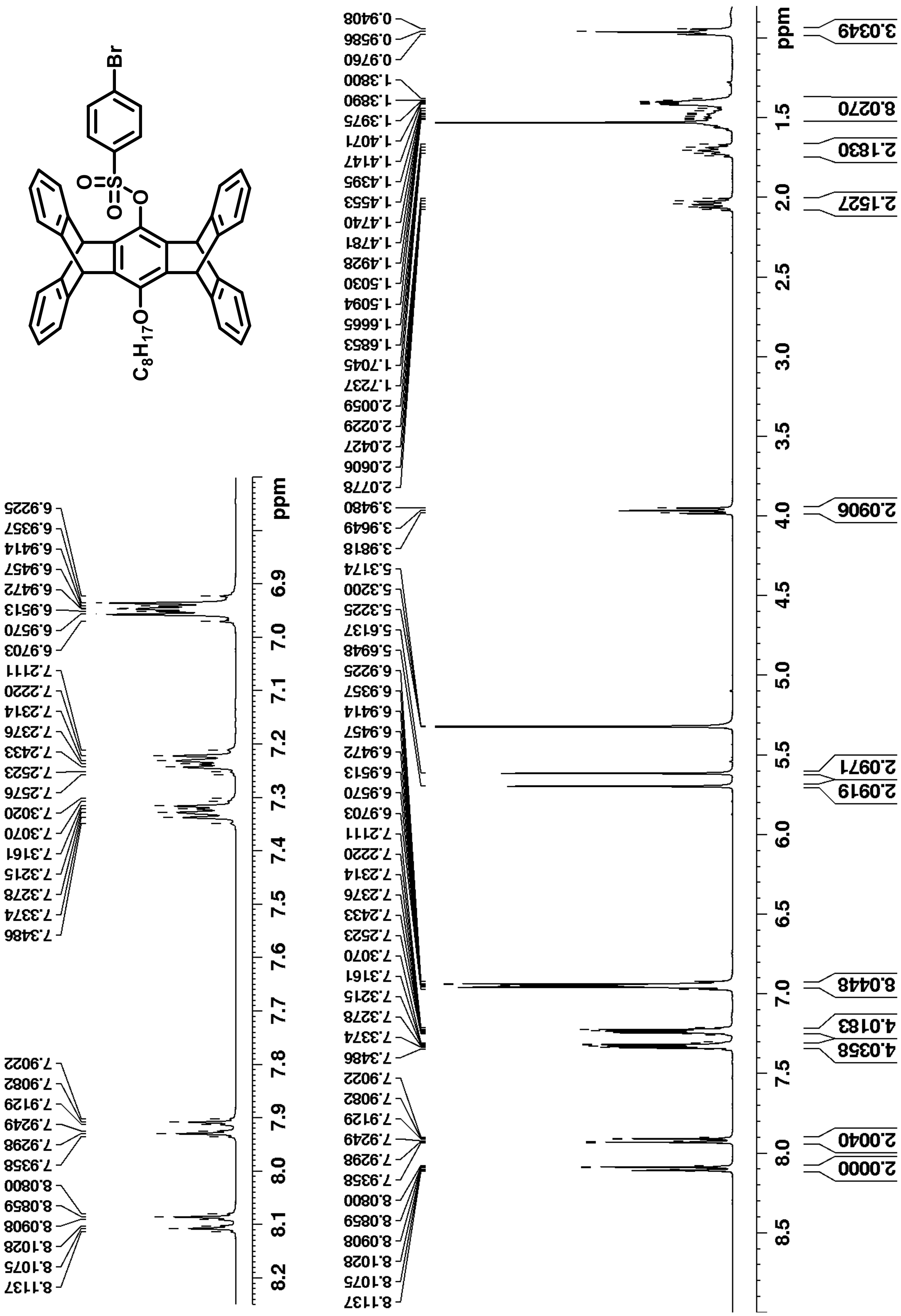

Figure S19. ^{1}H-NMR spectrum of compound **9** (400 MHz, CD_2Cl_2)

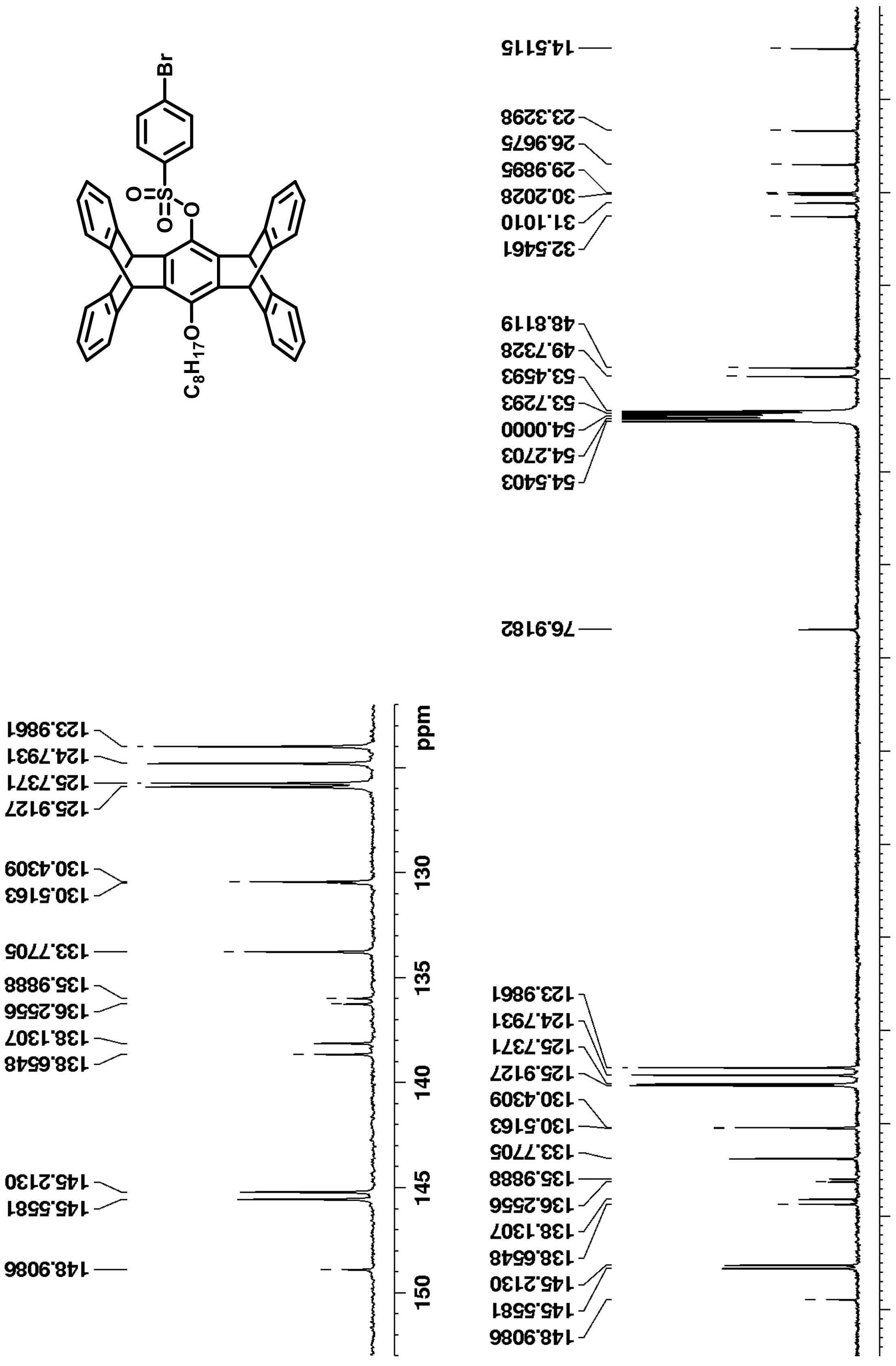

Figure S20. $^{13}C\{^{1}H\}$-NMR spectrum of compound **9** (100 MHz, CD_2Cl_2)

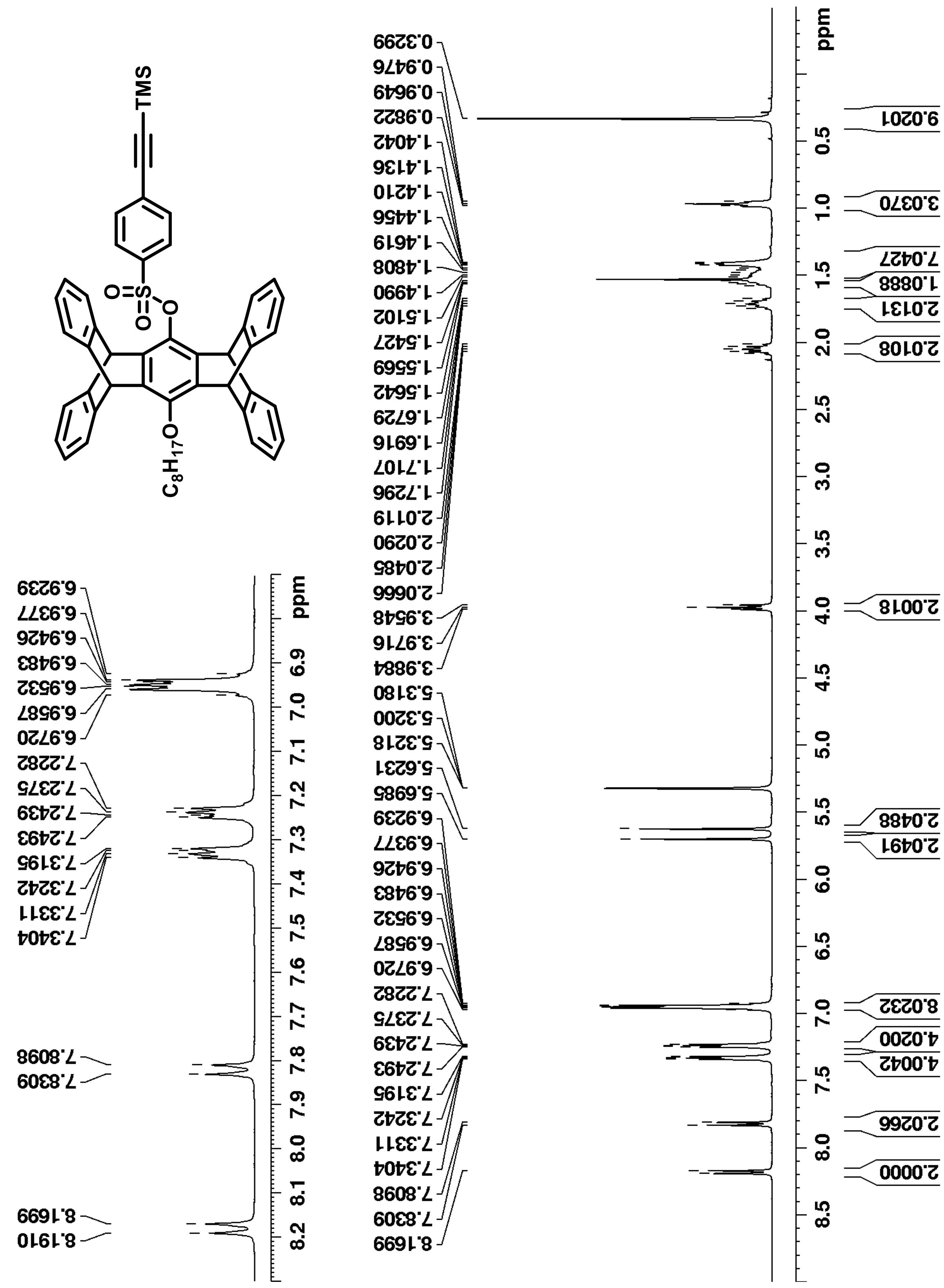

Figure S21. ^{1}H-NMR spectrum of compound **10** (400 MHz, CD_2Cl_2)

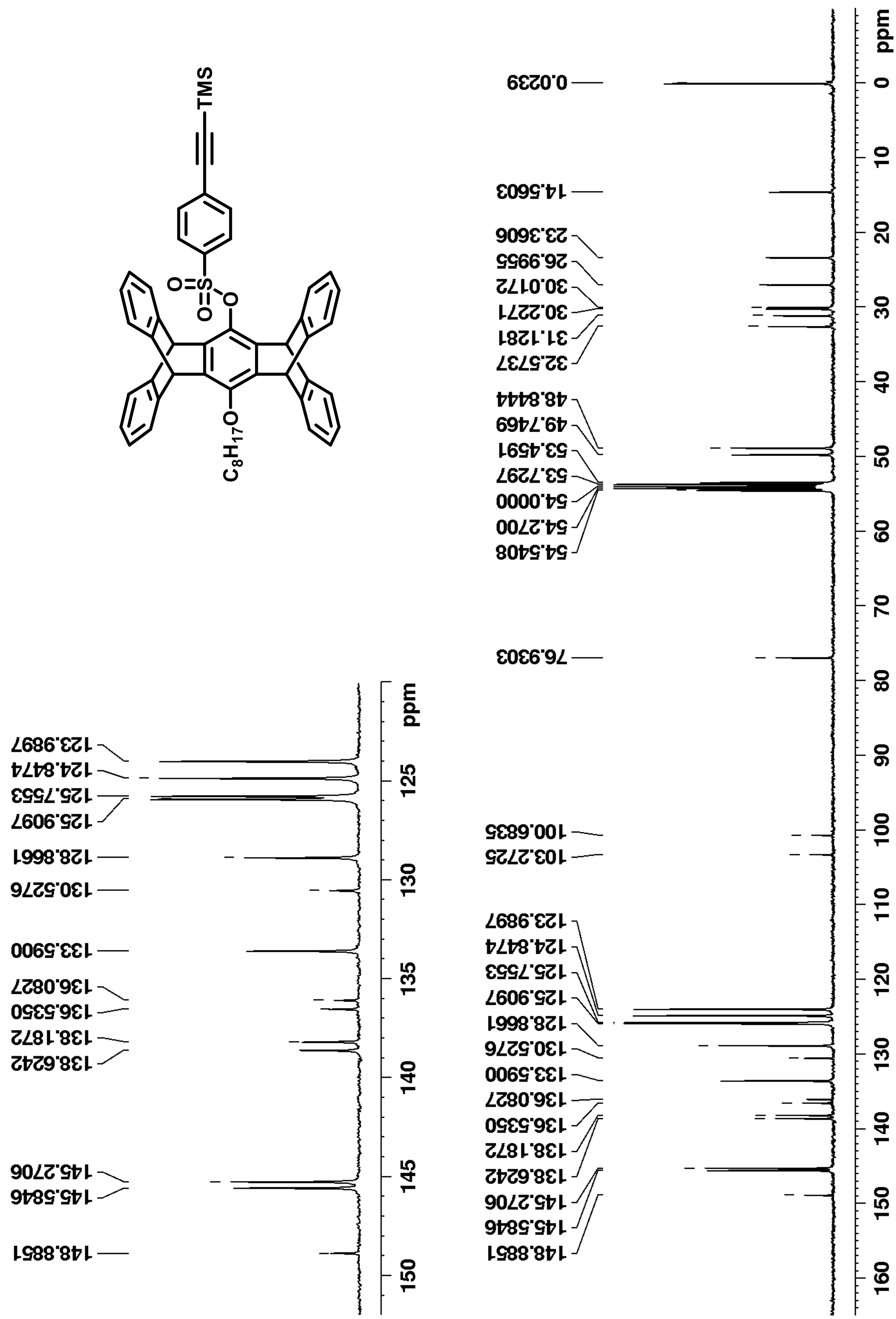

Figure S22. $^{13}C\{^1H\}$-NMR spectrum of compound **10** (100 MHz, CD_2Cl_2)

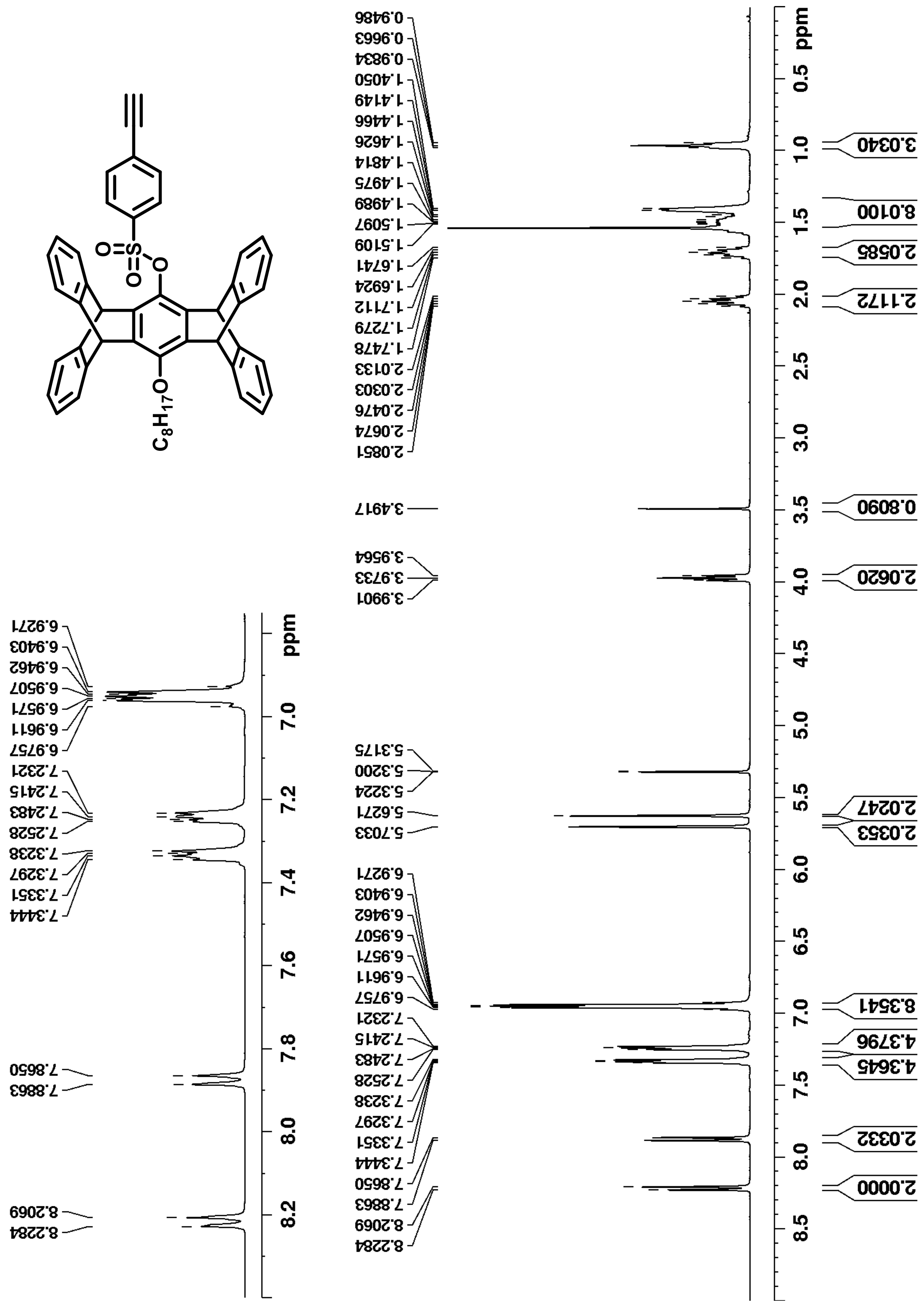

Figure S23. ^{1}H-NMR spectrum of compound **11** (400 MHz, CD_2Cl_2)

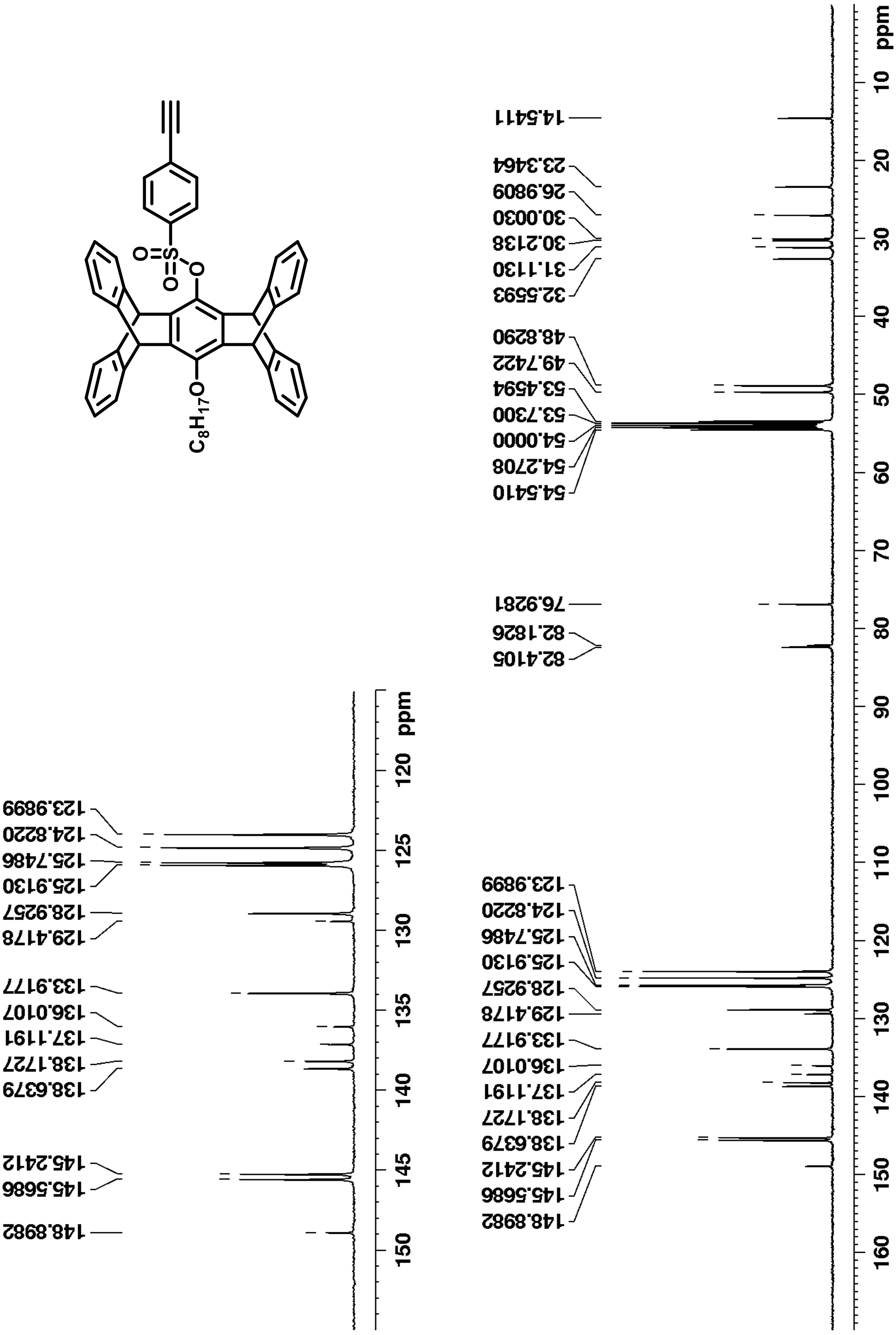

Figure S24. $^{13}C\{^{1}H\}$-NMR spectrum of compound **11** (100 MHz, CD_2Cl_2)

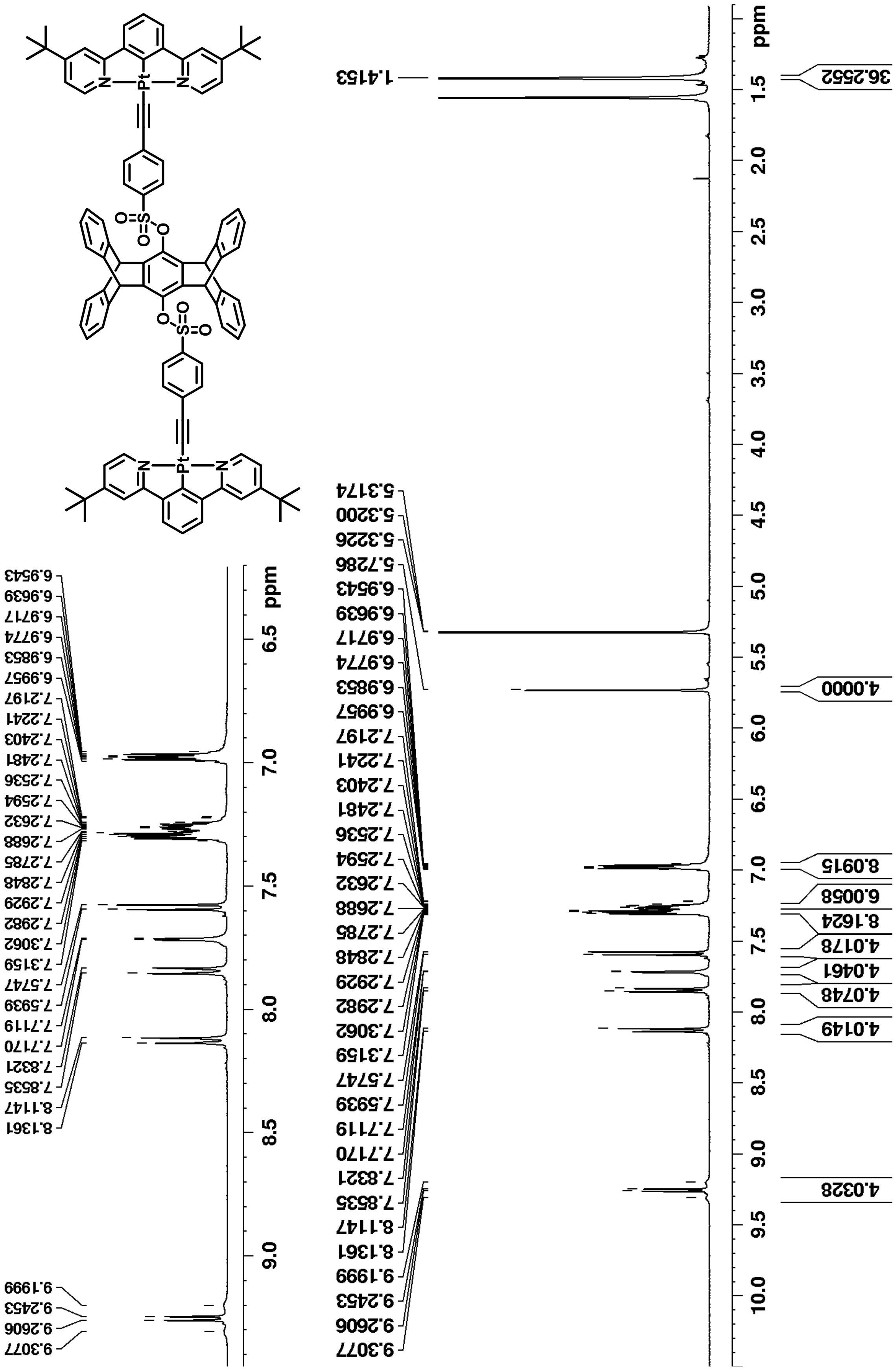

Figure S25. ^{1}H-NMR spectrum of **2** (400 MHz, CD_2Cl_2)

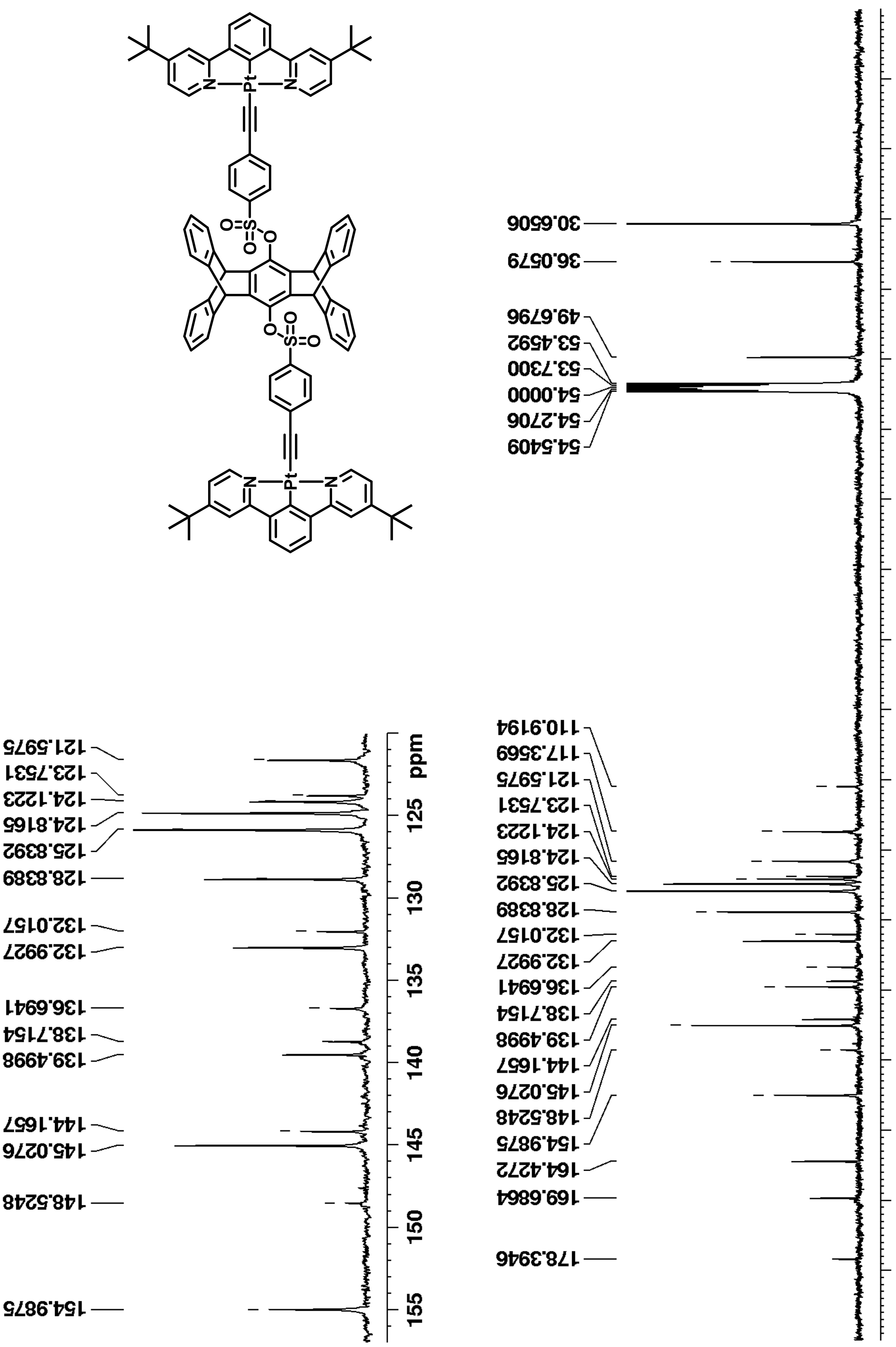

Figure S26. $^{13}C\{^{1}H\}$-NMR spectrum of **2** (100 MHz, CD_2Cl_2)

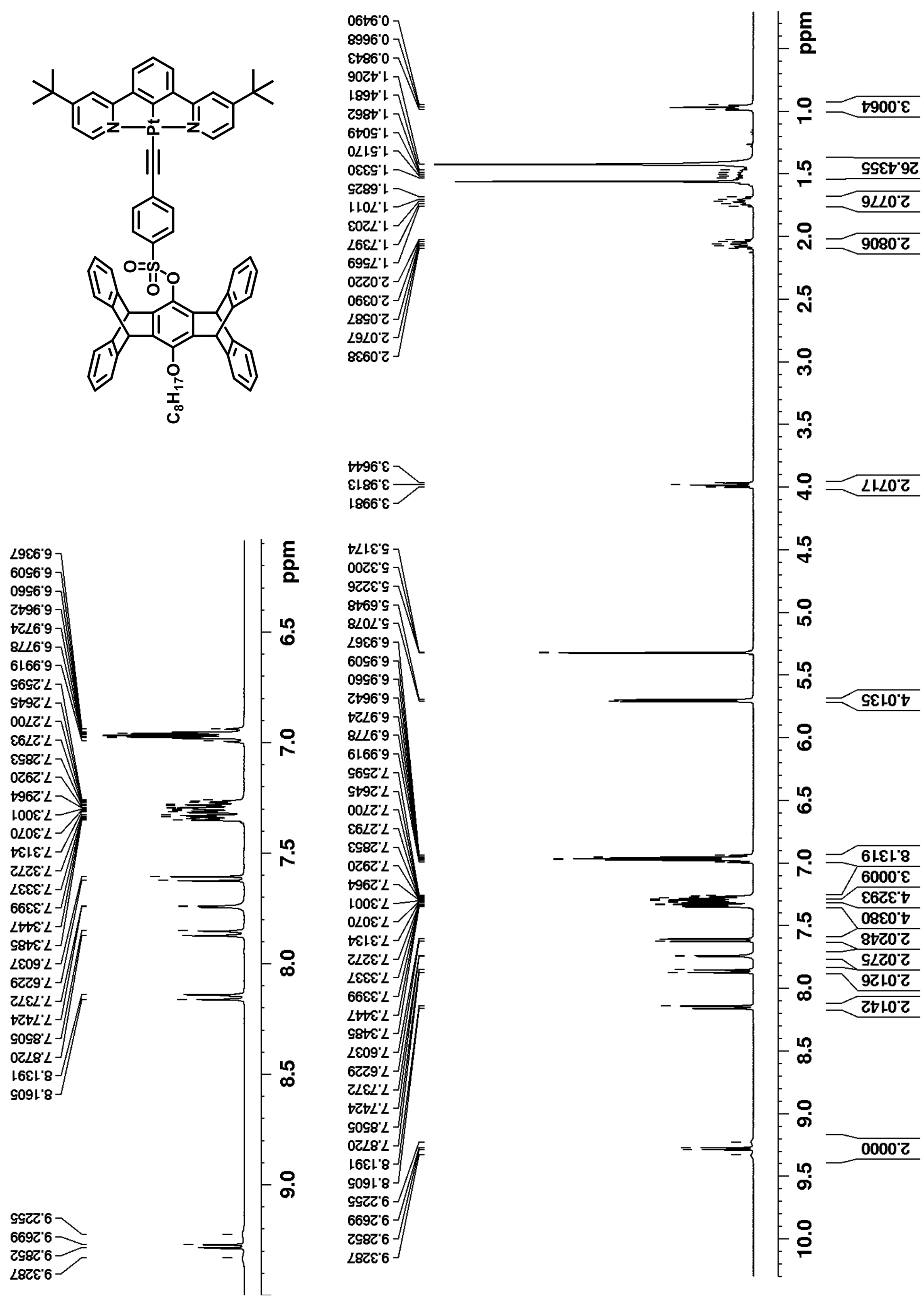

Figure S27. ^{1}H-NMR spectrum of **3** (400 MHz, CD_2Cl_2)

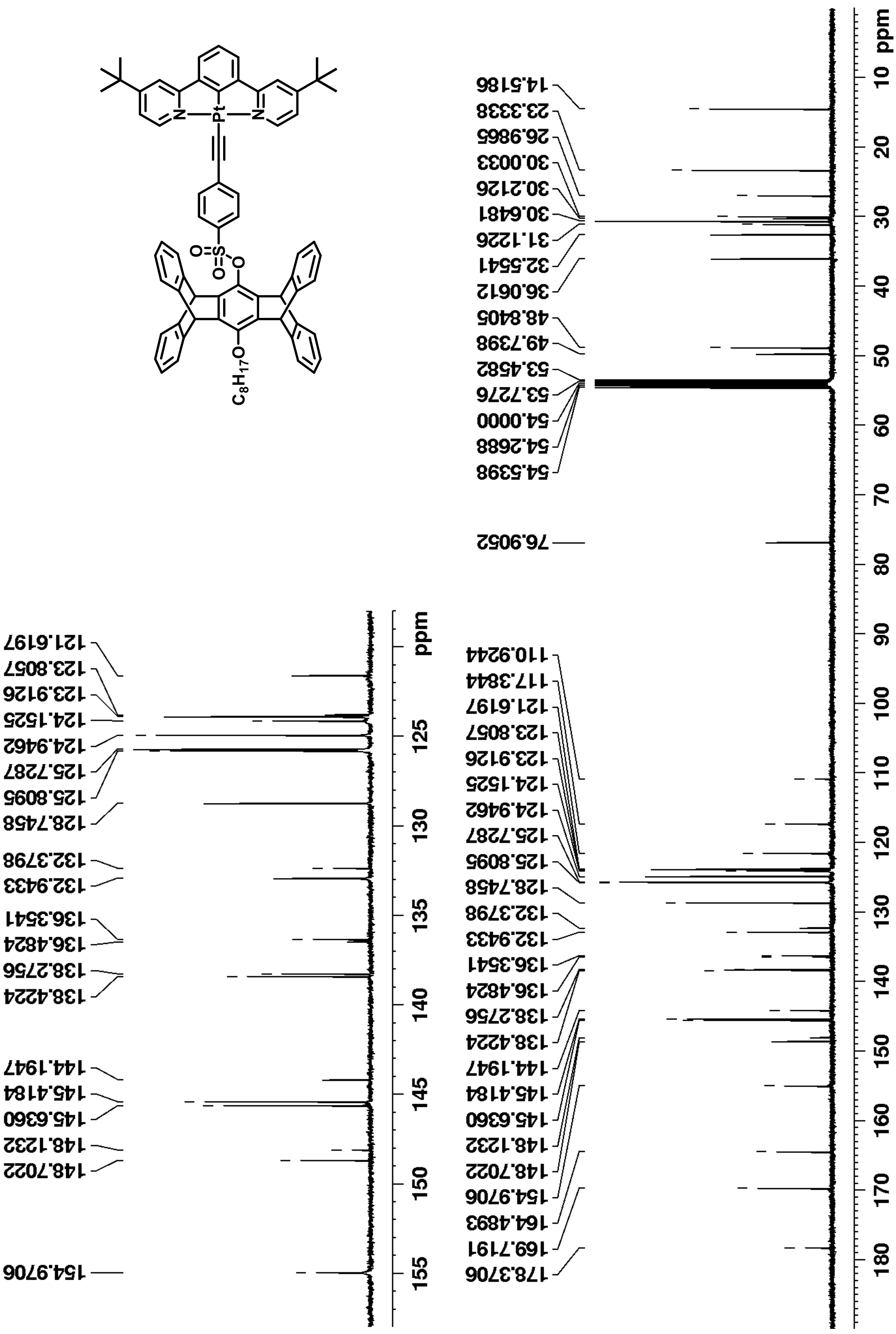

Figure S28. $^{13}C\{^{1}H\}$-NMR spectrum of **3** (100 MHz, CD_2Cl_2)

Table S5. Cartesian coordinates of the ground-state optimized structure of **3**.

Atom	X	Y	Z
Pt	-5.704147	0.076821	-0.000031
S	3.394561	-1.634275	-0.139329
O	3.943337	-0.096888	0.028624
O	3.790137	-2.107097	-1.452477
O	3.772551	-2.372685	1.050995
N	-5.769253	2.152812	0.106421
N	-6.385594	-1.885365	-0.0912
C	-4.710471	2.970797	0.138591
H	-3.744437	2.477442	0.102696
C	-4.844219	4.346614	0.213015
H	-3.947638	4.955703	0.236026
C	-6.118324	4.926325	0.257659
C	-7.208907	4.058724	0.222435
H	-8.222533	4.438865	0.253445
C	-7.028172	2.679226	0.147032
C	-8.11247	1.678052	0.105804
C	-9.493207	1.88805	0.13133
H	-9.916244	2.887743	0.186901
C	-10.352047	0.786191	0.084358
H	-11.42405	0.949533	0.104323
C	-9.862732	-0.521333	0.012686
H	-10.566297	-1.349205	-0.021395
C	-8.482468	-0.733117	-0.013086
C	-7.6274	0.370196	0.03355
C	-7.749815	-2.013251	-0.084162
C	-8.32765	-3.273589	-0.140187
H	-9.41045	-3.343568	-0.133297
C	-7.54375	-4.431289	-0.203404
C	-6.159522	-4.258358	-0.208279
H	-5.480812	-5.099955	-0.254984
C	-5.622599	-2.977483	-0.151623
H	-4.552492	-2.794563	-0.153011
C	-6.26566	6.444054	0.341612
C	-7.733448	6.879097	0.382753
H	-8.250719	6.471193	1.256734
H	-7.786831	7.969508	0.443493

H	-8.271582	6.569149	-0.518444
C	-5.598613	7.078861	-0.890666
H	-4.533343	6.838773	-0.942949
H	-6.07126	6.729655	-1.813302
H	-5.695822	8.167932	-0.844821
C	-5.568947	6.942646	1.619359
H	-4.503024	6.699789	1.62039
H	-5.665903	8.030135	1.693981
H	-6.020257	6.494927	2.509587
C	-8.213283	-5.802758	-0.263208
C	-9.096692	-5.873991	-1.520505
H	-8.498667	-5.733836	-2.425607
H	-9.582428	-6.853027	-1.576996
H	-9.879689	-5.110888	-1.509364
C	-9.084244	-5.990222	0.990855
H	-9.566948	-6.971989	0.96321
H	-8.477752	-5.930528	1.899202
H	-9.869519	-5.232213	1.056629
C	-7.187055	-6.938173	-0.32061
H	-6.55457	-6.865436	-1.210626
H	-6.543892	-6.945603	0.564703
H	-7.709373	-7.897938	-0.360987
C	-3.661913	-0.248864	-0.034046
C	-2.454423	-0.467484	-0.053242
C	-1.051843	-0.735328	-0.073712
C	-0.348131	-0.778749	-1.291973
H	-0.889928	-0.601353	-2.214195
C	1.010314	-1.053199	-1.317525
H	1.557927	-1.107116	-2.252625
C	1.671976	-1.285034	-0.113973
C	1.005792	-1.252822	1.109069
H	1.549546	-1.458996	2.025039
C	-0.352104	-0.976473	1.123847
H	-0.897018	-0.950162	2.060801
C	5.338288	0.040391	0.023678
C	6.004883	0.274606	-1.172373
C	7.387947	0.466553	-1.167083
C	7.969543	0.699571	-2.555324

H	9.049203	0.859426	-2.531763
C	7.200477	1.881812	-3.133988
C	5.813575	1.680161	-3.142541
C	5.400265	0.32938	-2.568937
H	4.322415	0.166653	-2.56913
C	6.161692	-0.721293	-3.373803
C	5.611925	-1.798295	-4.048482
H	4.541217	-1.971684	-4.013237
C	6.457068	-2.668451	-4.743899
H	6.035761	-3.517001	-5.273161
C	7.831567	-2.459179	-4.754741
H	8.479049	-3.141072	-5.296688
C	8.386057	-1.37691	-4.06444
H	9.460221	-1.210999	-4.067038
C	7.547978	-0.514465	-3.376674
C	7.741431	3.059736	-3.622113
H	8.816697	3.213369	-3.611361
C	6.887788	4.046275	-4.124928
H	7.302906	4.972578	-4.508896
C	5.511885	3.846706	-4.133837
H	4.855819	4.617717	-4.524912
C	4.967267	2.657552	-3.639746
H	3.892534	2.499635	-3.642997
O	9.47495	0.563764	0.017989
C	8.107443	0.416211	0.022901
C	7.426398	0.188282	1.214598
C	6.043174	-0.004362	1.219859
C	5.483827	-0.274924	2.610147
H	4.406036	-0.437466	2.607327
C	6.265943	-1.481066	3.125403
C	7.65253	-1.276278	3.131266
C	8.053089	0.096018	2.599782
H	9.132159	0.260123	2.578969
C	7.308336	1.110287	3.460299
C	7.870059	2.145187	4.189581
H	8.945124	2.30031	4.180023
C	7.037454	2.986313	4.933921
H	7.468773	3.800131	5.507907

C	5.661647	2.785997	4.940782
H	5.021935	3.443688	5.520461
C	5.096066	1.741589	4.203141
H	4.02128	1.584012	4.204502
C	5.921523	0.907978	3.466579
C	5.733101	-2.68696	3.549034
H	4.66108	-2.85056	3.50859
C	6.596465	-3.692005	3.995879
H	6.188686	-4.641581	4.327105
C	7.971535	-3.486691	4.01067
H	8.632955	-4.27378	4.358464
C	8.508462	-2.272093	3.572695
H	9.582898	-2.108054	3.578973
C	10.134827	-0.685189	-0.141913
H	9.882009	-1.368969	0.678886
H	11.206558	-0.482275	-0.136968
H	9.850422	-1.159223	-1.090395

Table S6. Cartesian coordinates of the excited-state optimized structure of **3**.

Atom	X	Y	Z
Pt	-5.710168	0.080857	0.005218
S	3.392452	-1.626036	-0.132604
O	3.94308	-0.089757	0.031603
O	3.783637	-2.102329	-1.445693
O	3.769694	-2.363604	1.058361
N	-5.756776	2.123058	0.1077
N	-6.36445	-1.908487	-0.087979
C	-4.726711	2.957912	0.145793
H	-3.747757	2.4885	0.116811
C	-4.874551	4.340211	0.218425
H	-3.980388	4.95144	0.245748
C	-6.176734	4.935321	0.256559
C	-7.247299	4.082677	0.216889
H	-8.263314	4.459583	0.241997
C	-7.078024	2.6617	0.141917
C	-8.098049	1.711647	0.098757
C	-9.528975	1.890399	0.114934
H	-9.967637	2.881449	0.16596

C	-10.338816	0.787785	0.064478
H	-11.415245	0.928714	0.076872
C	-9.841504	-0.561007	-0.004509
H	-10.550414	-1.38129	-0.041186
C	-8.461811	-0.763755	-0.021765
C	-7.609057	0.336928	0.027733
C	-7.726658	-2.043186	-0.08717
C	-8.300476	-3.303716	-0.143237
H	-9.38288	-3.378832	-0.141145
C	-7.509002	-4.458758	-0.200365
C	-6.126748	-4.279388	-0.199174
H	-5.443913	-5.117874	-0.240957
C	-5.595588	-2.994199	-0.142558
H	-4.526199	-2.806394	-0.139363
C	-6.302672	6.4523	0.338093
C	-7.765038	6.903865	0.371193
H	-8.289611	6.499803	1.242738
H	-7.809788	7.994921	0.430305
H	-8.300344	6.595629	-0.532408
C	-5.622385	7.080417	-0.890473
H	-4.561689	6.820034	-0.943942
H	-6.101169	6.741947	-1.81402
H	-5.697293	8.171406	-0.842484
C	-5.607253	6.946521	1.618336
H	-4.546005	6.683255	1.630848
H	-5.68268	8.036297	1.68801
H	-6.074775	6.510764	2.506203
C	-8.172062	-5.833178	-0.260117
C	-9.049542	-5.910952	-1.521221
H	-8.448124	-5.7703	-2.423982
H	-9.530694	-6.89222	-1.577581
H	-9.836089	-5.151423	-1.515401
C	-9.047763	-6.021563	0.990551
H	-9.526165	-7.005394	0.96279
H	-8.445556	-5.957499	1.90144
H	-9.836665	-5.266887	1.051359
C	-7.140569	-6.964101	-0.3104
H	-6.504532	-6.890596	-1.197811

H	-6.501374	-6.966693	0.5778
H	-7.658439	-7.926239	-0.350883
C	-3.661972	-0.230971	-0.021634
C	-2.454726	-0.451841	-0.040105
C	-1.051903	-0.720567	-0.061581
C	-0.350448	-0.76279	-1.280757
H	-0.892716	-0.582991	-2.202214
C	1.007707	-1.039082	-1.307647
H	1.554455	-1.092493	-2.243259
C	1.669782	-1.273196	-0.104951
C	1.00517	-1.241532	1.118738
H	1.549672	-1.449235	2.033884
C	-0.352568	-0.963613	1.135316
H	-0.896414	-0.937314	2.072872
C	5.338578	0.045877	0.023585
C	6.002775	0.277379	-1.174258
C	7.386132	0.467349	-1.171976
C	7.965252	0.697843	-2.561667
H	9.045184	0.85614	-2.540453
C	7.196742	1.880594	-3.140102
C	5.809547	1.681053	-3.145561
C	5.395444	0.33146	-2.569676
H	4.31733	0.170423	-2.567636
C	6.153635	-0.721245	-3.374897
C	5.60089	-1.79803	-4.047469
H	4.52995	-1.969616	-4.010199
C	6.443289	-2.670201	-4.743688
H	6.019636	-3.518564	-5.271373
C	7.818081	-2.463064	-4.757412
H	8.463434	-3.146456	-5.300004
C	8.375595	-1.380955	-4.069297
H	9.449995	-1.216664	-4.074269
C	7.540228	-0.516521	-3.380735
C	7.738455	3.057166	-3.630628
H	8.813962	3.209224	-3.622279
C	6.885216	4.044445	-4.132667
H	7.300888	4.969737	-4.518442
C	5.50899	3.846983	-4.138431

H	4.853255	4.618621	-4.528791
C	4.963622	2.659177	-3.641961
H	3.888632	2.502999	-3.642722
O	9.475528	0.56352	0.008807
C	8.107907	0.417755	0.016677
C	7.428984	0.192727	1.210146
C	6.045507	0.002038	1.218534
C	5.488514	-0.265025	2.610462
H	4.410456	-0.42603	2.610174
C	6.269923	-1.471226	3.126638
C	7.656817	-1.268428	3.129213
C	8.058267	0.102228	2.594259
H	9.13752	0.264751	2.570972
C	7.316628	1.119347	3.454139
C	7.881245	2.155111	4.179946
H	8.956499	2.308718	4.167935
C	7.051258	2.999154	4.923899
H	7.484822	3.813734	5.49509
C	5.675175	2.800872	4.933845
H	5.03751	3.460901	5.513098
C	5.106677	1.755575	4.199726
H	4.031653	1.599687	4.203451
C	5.929556	0.919085	3.463555
C	5.736242	-2.675382	3.55413
H	4.66387	-2.837439	3.516663
C	6.599052	-3.680736	4.00135
H	6.190591	-4.62894	4.335652
C	7.974448	-3.477424	4.012707
H	8.635456	-4.264705	4.360839
C	8.512207	-2.264555	3.570978
H	9.586884	-2.102055	3.574709
C	10.133581	-0.686452	-0.150814
H	9.881657	-1.368817	0.671433
H	11.205554	-0.484809	-0.148364
H	9.846662	-1.161404	-1.098081

Table S7. Cartesian coordinates of the ground-state optimized structure of **2**.

Atom	X	Y	Z
C	5.152196	-1.524498	1.152179
C	6.067825	-0.53324	1.495402
C	7.010578	-0.104784	0.562542
C	7.107131	-0.729186	-0.675676
C	6.155205	-1.692046	-1.029968
C	5.167183	-2.058674	-0.130851
C	6.363889	-2.270516	-2.420505
C	8.173826	-0.526009	-1.751037
C	6.431487	-1.078777	-3.368949
C	7.419805	-0.148373	-3.02035
C	7.628641	0.984107	-3.793373
C	6.822388	1.19608	-4.916329
C	5.82918	0.282028	-5.254078
C	5.63157	-0.865885	-4.480541
C	8.743925	-1.919097	-2.010622
C	7.76999	-2.859372	-2.371218
C	8.113576	-4.178733	-2.614439
C	9.456379	-4.555856	-2.515806
C	10.427514	-3.621478	-2.172265
C	10.073822	-2.293763	-1.912535
C	4.279947	-1.963496	2.31509
C	5.985077	-0.116253	2.961103
C	3.622555	-0.720857	2.896375
C	4.542371	0.268325	3.266494
C	4.114611	1.406938	3.933386
C	2.754152	1.567749	4.211037
C	1.839766	0.592222	3.823271
C	2.271081	-0.559615	3.158977
C	6.199592	-1.422433	3.731029
C	5.282185	-2.421955	3.377132
C	5.349231	-3.674369	3.962096
C	6.333364	-3.923709	4.923798
C	7.236233	-2.929534	5.282353
C	7.175855	-1.668225	4.681068
O	7.841169	0.953054	0.915902
O	4.135381	-2.895241	-0.549694

S	7.516357	2.433387	0.242228
C	5.772573	2.420283	-0.031548
S	4.205748	-4.513106	-0.278
C	2.47021	-4.706999	-0.052017
O	8.189056	2.472788	-1.041965
C	4.919585	2.736995	1.022257
C	3.548825	2.702754	0.811235
C	3.022901	2.368436	-0.450382
C	3.912083	2.045592	-1.491877
C	5.282905	2.069489	-1.2895
C	1.610874	2.403671	-0.679291
C	1.639577	-4.583735	-1.163535
C	0.278852	-4.41825	-0.963276
C	-0.255167	-4.346462	0.337635
C	0.596904	-4.573769	1.433627
C	1.960452	-4.742692	1.243619
O	4.911407	-4.74225	0.966822
C	-1.606302	-3.932997	0.540941
C	0.404403	2.504025	-0.884753
O	7.859172	3.364204	1.293814
O	4.642409	-5.134834	-1.512419
C	-2.696899	-3.395646	0.712954
H	5.602759	-3.004974	-2.688915
H	8.925569	0.209212	-1.465862
H	8.384659	1.706426	-3.501861
H	6.970223	2.083038	-5.523947
H	5.205699	0.457832	-6.124891
H	4.861448	-1.584647	-4.745482
H	7.34466	-4.904508	-2.862501
H	9.740275	-5.586261	-2.703816
H	11.466805	-3.924843	-2.096769
H	10.828694	-1.564912	-1.632087
H	3.5614	-2.734714	2.039806
H	6.706873	0.661838	3.216986
H	4.833986	2.157645	4.250381
H	2.409949	2.451022	4.741046
H	0.787113	0.71037	4.063905
H	1.56309	-1.334473	2.875237

H	4.660783	-4.456758	3.658423
H	6.395804	-4.903314	5.386334
H	7.998891	-3.133935	6.02681
H	7.887315	-0.89268	4.94923
H	5.331913	3.014904	1.986684
H	2.8669	2.932374	1.623909
H	3.513362	1.784191	-2.465776
H	5.967739	1.832709	-2.096576
H	2.067544	-4.580008	-2.160714
H	-0.387775	-4.291565	-1.809676
H	0.178065	-4.563936	2.433854
H	2.635245	-4.870147	2.084043
Pt	-1.614797	2.935712	-1.096968
Pt	-4.350797	-2.160812	0.880441
N	-2.438137	1.076992	-1.545793
N	-1.517522	4.991918	-0.768443
C	-3.497656	3.407824	-1.234646
C	-4.428511	2.403424	-1.511009
C	-5.784796	2.729483	-1.555826
C	-6.176362	4.054958	-1.332889
C	-5.241484	5.061839	-1.080178
C	-3.882269	4.735549	-1.034587
C	-1.761001	-0.058595	-1.749992
C	-2.371821	-1.21331	-2.215769
C	-3.743716	-1.217715	-2.490039
C	-4.444841	-0.041911	-2.210875
C	-3.789229	1.093199	-1.749043
C	-2.732034	5.626763	-0.785107
C	-2.803173	6.993594	-0.555472
C	-1.652624	7.749782	-0.303862
C	-0.435406	7.067891	-0.301603
C	-0.409868	5.698187	-0.537931
C	-4.420226	-2.411648	-3.160605
C	-5.932282	-2.416076	-2.898918
C	-4.175577	-2.279937	-4.676237
C	-3.825798	-3.739318	-2.671094
C	-1.768492	9.250212	-0.043957
C	-2.415255	9.923338	-1.266536

C	-2.651007	9.474222	1.196112
C	-0.401712	9.895037	0.204311
C	-5.842771	-0.916503	0.983535
N	-5.944984	-3.449804	0.514611
N	-3.337139	-0.395974	1.330333
C	-7.172287	-2.838948	0.48982
C	-8.311393	-3.583327	0.215831
C	-8.239817	-4.957378	-0.03867
C	-6.974145	-5.543549	0.003547
C	-5.86033	-4.760558	0.282801
C	-2.019121	-0.264017	1.518995
C	-1.444851	0.918382	1.963906
C	-2.256241	2.021453	2.252086
C	-3.622262	1.885716	1.985325
C	-4.150056	0.684973	1.526561
C	-5.582802	0.425788	1.271659
C	-6.647104	1.328184	1.300889
C	-7.944249	0.86533	1.048739
C	-8.202325	-0.480613	0.778375
C	-7.136753	-1.386222	0.748787
C	-9.512603	-5.742322	-0.348405
C	-10.480979	-5.618238	0.840369
C	-9.224429	-7.22655	-0.592409
C	-10.165867	-5.153603	-1.610742
C	-1.69651	3.274832	2.922384
C	-2.562524	4.503905	2.614422
C	-1.714939	3.021076	4.442422
C	-0.252883	3.554994	2.482868
H	-6.543919	1.973917	-1.742518
H	-7.231248	4.306601	-1.362743
H	-5.587434	6.08055	-0.924071
H	-0.695926	-0.007276	-1.546088
H	-1.762578	-2.096977	-2.359055
H	-5.515253	0.017674	-2.368499
H	-3.779199	7.467504	-0.571308
H	0.502328	7.576581	-0.119641
H	0.513439	5.125183	-0.542403
H	-6.438803	-1.596619	-3.41764

H	-6.36613	-3.35115	-3.266224
H	-6.143592	-2.332019	-1.829573
H	-4.591167	-1.343674	-5.061133
H	-4.652406	-3.11277	-5.203643
H	-3.105468	-2.29683	-4.903321
H	-3.862443	-3.815273	-1.581571
H	-2.782226	-3.85582	-2.978047
H	-4.385523	-4.571079	-3.110797
H	-3.418006	9.533445	-1.461441
H	-2.501533	11.000497	-1.093762
H	-1.809486	9.766327	-2.16363
H	-2.738684	10.545723	1.400493
H	-2.216137	8.990836	2.076076
H	-3.659299	9.076	1.053163
H	-0.532549	10.965585	0.384163
H	0.092634	9.467705	1.082097
H	0.260856	9.780588	-0.658958
H	-9.267835	-3.071187	0.198456
H	-6.829719	-6.600808	-0.176845
H	-4.857699	-5.175328	0.322718
H	-1.431007	-1.153731	1.313771
H	-0.367375	0.961061	2.073915
H	-4.303407	2.713393	2.145402
H	-6.487656	2.385471	1.496719
H	-8.769366	1.569375	1.067431
H	-9.225733	-0.799448	0.597482
H	-11.397345	-6.178672	0.631363
H	-10.759771	-4.578183	1.030087
H	-10.031862	-6.021251	1.752669
H	-8.556818	-7.372955	-1.447078
H	-8.775414	-7.699774	0.286194
H	-10.16108	-7.747381	-0.808894
H	-11.077379	-5.709642	-1.850468
H	-9.487886	-5.219032	-2.46698
H	-10.43928	-4.10383	-1.473594
H	-2.061301	5.406831	2.976299
H	-2.725789	4.60542	1.538701
H	-3.537313	4.452748	3.108963

H	-2.730008	2.816456	4.796645
H	-1.335467	3.902387	4.970099
H	-1.084059	2.166307	4.705669
H	-0.16996	3.626946	1.395755
H	0.436346	2.771971	2.81337
H	0.08412	4.495289	2.931952

Table S8. Cartesian coordinates of the excited-state optimized structure of **2**.

Atom	X	Y	Z
C	5.23612	-1.123518	1.194034
C	6.094246	-0.053943	1.434399
C	6.998981	0.345124	0.452354
C	7.123221	-0.382519	-0.725616
C	6.228851	-1.429057	-0.977888
C	5.270966	-1.768571	-0.036519
C	6.457813	-2.115671	-2.314991
C	8.163802	-0.211994	-1.831776
C	6.442041	-1.01164	-3.366272
C	7.374705	0.004228	-3.117359
C	7.505228	1.073566	-3.990962
C	6.675711	1.13512	-5.11529
C	5.737228	0.136166	-5.354779
C	5.618879	-0.947766	-4.47945
C	8.81588	-1.585808	-1.975838
C	7.897959	-2.611781	-2.236064
C	8.319614	-3.924421	-2.366806
C	9.684067	-4.209342	-2.256541
C	10.599339	-3.190993	-2.012734
C	10.167285	-1.869163	-1.866152
C	4.399438	-1.50417	2.402717
C	5.997463	0.490195	2.856888
C	3.674251	-0.253877	2.876974
C	4.536824	0.816629	3.144152
C	4.047498	1.984764	3.71002
C	2.681934	2.091176	3.9888
C	1.823841	1.032882	3.702774
C	2.317524	-0.147938	3.140647
C	6.294179	-0.725497	3.73952

C	5.434663	-1.804751	3.489357
C	5.579605	-2.992451	4.184753
C	6.583636	-3.095332	5.15269
C	7.428931	-2.021855	5.408141
C	7.290037	-0.826392	4.695633
O	7.752066	1.489533	0.690047
O	4.276291	-2.684421	-0.370078
S	7.279843	2.868557	-0.100888
C	5.538492	2.663773	-0.306231
S	4.396703	-4.266384	0.052771
C	2.663942	-4.474128	0.285671
O	7.910054	2.849906	-1.406915
C	4.690035	2.9872	0.749432
C	3.325462	2.786326	0.601212
C	2.798917	2.276303	-0.600257
C	3.683694	1.960614	-1.648138
C	5.049298	2.149824	-1.507232
C	1.385601	2.130786	-0.767262
C	1.842267	-4.466143	-0.839356
C	0.47942	-4.289586	-0.667199
C	-0.064461	-4.091507	0.616378
C	0.776985	-4.215558	1.737072
C	2.143081	-4.393274	1.575143
O	5.099058	-4.357572	1.317061
C	-1.416088	-3.658207	0.766015
C	0.168509	2.064586	-0.920839
O	7.562217	3.918736	0.851558
O	4.857114	-4.989189	-1.116238
C	-2.500199	-3.090136	0.867665
H	5.740252	-2.915476	-2.505283
H	8.871593	0.590063	-1.62507
H	8.218317	1.863435	-3.776724
H	6.762228	1.971467	-5.801429
H	5.094987	0.19534	-6.227691
H	4.891769	-1.732575	-4.667299
H	7.594326	-4.714623	-2.53726
H	10.028932	-5.233384	-2.356954
H	11.65608	-3.423029	-1.927228

H	10.878738	-1.073873	-1.663528
H	3.725388	-2.337489	2.207514
H	6.673632	1.329311	3.031735
H	4.723653	2.801785	3.948434
H	2.290095	2.997797	4.440525
H	0.767787	1.111313	3.944728
H	1.654392	-0.985249	2.937337
H	4.936278	-3.837938	3.962062
H	6.707014	-4.023288	5.70145
H	8.207645	-2.112794	6.158459
H	7.956486	0.010369	4.883334
H	5.100582	3.393871	1.667809
H	2.649622	3.014636	1.419322
H	3.285021	1.567042	-2.576555
H	5.729585	1.911242	-2.317611
H	2.279293	-4.551774	-1.828902
H	-0.18024	-4.245564	-1.527285
H	0.34929	-4.111319	2.728127
H	2.811583	-4.433142	2.429199
Pt	-1.900067	2.176503	-1.03522
Pt	-4.090244	-1.764449	0.853116
N	-2.45449	0.183062	-1.263731
N	-2.097418	4.246508	-0.894815
C	-3.834395	2.352758	-1.086385
C	-4.617079	1.199042	-1.210937
C	-6.009515	1.318856	-1.192219
C	-6.584163	2.590221	-1.060443
C	-5.801077	3.74378	-0.96073
C	-4.405427	3.626183	-0.978305
C	-1.625885	-0.856175	-1.413607
C	-2.079594	-2.127197	-1.735017
C	-3.448884	-2.358839	-1.915254
C	-4.303575	-1.277364	-1.692612
C	-3.803118	-0.018414	-1.37595
C	-3.395068	4.693647	-0.885881
C	-3.660658	6.05271	-0.772432
C	-2.62703	6.988874	-0.665364
C	-1.320935	6.494353	-0.684606

C	-1.099783	5.126551	-0.801279
C	-3.971042	-3.697553	-2.433149
C	-5.448876	-3.898082	-2.071861
C	-3.829794	-3.67771	-3.967448
C	-3.157112	-4.87106	-1.870941
C	-2.954587	8.474646	-0.530703
C	-3.759351	8.925825	-1.761513
C	-3.793805	8.687015	0.741066
C	-1.689666	9.332367	-0.431612
C	-5.51385	-0.443117	0.773156
N	-5.742254	-3.004056	0.58905
N	-2.99353	-0.014127	1.131717
C	-6.935289	-2.337865	0.461839
C	-8.107219	-3.050044	0.241046
C	-8.103867	-4.445367	0.143826
C	-6.871779	-5.086693	0.286493
C	-5.723536	-4.335089	0.507405
C	-1.67527	0.071439	1.343037
C	-1.04896	1.266011	1.67043
C	-1.806442	2.435086	1.810151
C	-3.170648	2.339889	1.519862
C	-3.751708	1.122832	1.182092
C	-5.188288	0.909383	0.916354
C	-6.204034	1.862742	0.813252
C	-7.518945	1.439771	0.578203
C	-7.843658	0.086617	0.454258
C	-6.827286	-0.870884	0.556867
C	-9.409789	-5.194904	-0.111517
C	-10.396183	-4.88864	1.028464
C	-9.195896	-6.709857	-0.17998
C	-10.003042	-4.720671	-1.449421
C	-1.195902	3.725727	2.353526
C	-1.986469	4.955082	1.885404
C	-1.264075	3.645775	3.891136
C	0.270801	3.880842	1.928773
H	-6.654852	0.446733	-1.260667
H	-7.66504	2.681282	-1.041174
H	-6.28919	4.710926	-0.871795

H	-0.57132	-0.631321	-1.28741
H	-1.35203	-2.922093	-1.841562
H	-5.377546	-1.390218	-1.782567
H	-4.696663	6.375484	-0.764482
H	-0.462798	7.149608	-0.610883
H	-0.101477	4.697708	-0.817115
H	-6.100598	-3.214954	-2.624835
H	-5.754703	-4.917355	-2.32695
H	-5.613543	-3.743903	-1.002118
H	-4.402474	-2.853582	-4.403657
H	-4.201798	-4.618052	-4.387647
H	-2.783045	-3.559525	-4.263043
H	-3.124242	-4.846847	-0.778928
H	-2.125636	-4.862673	-2.235437
H	-3.606641	-5.813987	-2.198478
H	-4.700649	8.377295	-1.854199
H	-3.998015	9.99063	-1.678427
H	-3.185198	8.772683	-2.679815
H	-4.031327	9.749081	0.856303
H	-3.244939	8.359174	1.62897
H	-4.736561	8.134485	0.701163
H	-1.97089	10.384841	-0.337869
H	-1.090659	9.068191	0.445264
H	-1.064067	9.23269	-1.323816
H	-9.03428	-2.495312	0.139816
H	-6.780327	-6.163522	0.229448
H	-4.74593	-4.793576	0.621252
H	-1.131022	-0.864393	1.255878
H	0.02634	1.265877	1.806778
H	-3.809729	3.213979	1.566945
H	-5.992922	2.925971	0.893961
H	-8.305315	2.182356	0.494395
H	-8.878525	-0.197821	0.281851
H	-11.336342	-5.422136	0.858051
H	-10.623247	-3.821063	1.092915
H	-9.989294	-5.208048	1.992224
H	-8.518426	-6.985489	-0.994022
H	-8.792123	-7.102759	0.758156

H	-10.153999	-7.203887	-0.362852
H	-10.937432	-5.253521	-1.650789
H	-9.310858	-4.916632	-2.273776
H	-10.223061	-3.649665	-1.438146
H	-1.44598	5.866158	2.15973
H	-2.118037	4.943443	0.800618
H	-2.974163	5.009653	2.353297
H	-2.296864	3.534398	4.235572
H	-0.850113	4.560236	4.328824
H	-0.686813	2.794881	4.266251
H	0.383206	3.826167	0.843321
H	0.908721	3.105771	2.364376
H	0.647262	4.847204	2.280631

References

[S1] Frisch, M. J.; Trucks, G. W.; Schlegel, H. B.; Scuseria, G. E.; Robb, M. A.; Cheeseman, J. R.; Scalmani, G.; Barone, V.; Petersson, G. A.; Nakatsuji, H.; Li, X.; Caricato, M.; Marenich, A. V.; Bloino, J.; Janesko, B. G.; Gomperts, R.; Mennucci, B.; Hratchian, H. P.; Ortiz, J. V.; Izmaylov, A. F.; Sonnenberg, J. L.; Williams-Young, D.; Ding, F.; Lipparini, F.; Egidi, F.; Goings, J.; Peng, B.; Petrone, A.; Henderson, T.; Ranasinghe, D.; Zakrzewski, V. G.; Gao, J.; Rega, N.; Zheng, G.; Liang, W.; Hada, M.; Ehara, M.; Toyota, K.; Fukuda, R.; Hasegawa, J.; Ishida, M.; Nakajima, T.; Honda, Y.; Kitao, O.; Nakai, H.; Vreven, T.; Throssell, K.; Montgomery, J. A., Jr.; Peralta, J. E.; Ogliaro, F.; Bearpark, M. J.; Heyd, J. J.; Brothers, E. N.; Kudin, K. N.; Staroverov, V. N.; Keith, T. A.; Kobayashi, R.; Normand, J.; Raghavachari, K.; Rendell, A. P.; Burant, J. C.; Iyengar, S. S.; Tomasi, J.; Cossi, M.; Millam, J. M.; Klene, M.; Adamo, C.; Cammi, R.; Ochterski, J. W.; Martin, R. L.; Morokuma, K.; Farkas, O.; Foresman, J. B.; Fox, D. J., Gaussian 16, Revision A.03, Gaussian, Inc., Wallingford, CT, **2016**.

[S2] Zhao, Y. and Truhlar, D. G. *Theor. Chem. Acc*. **2008**, *120*, 215−241.

[S3] D. Andrae, U. Häußermann, M. Dolg, H. Stoll and H. Preuss, *Theor. Chim. Acta*, **1990**, *77*, 123

[S4] Martin, R. L., Natural transition orbitals. *J. Chem. Phys.* **2003**, *118*, 4775-4777.

[S5] Lu, T.; Chen, F., Multiwfn: A multifunctional wavefunction analyzer. *J. Comput. Chem.* **2012,** *33*, 580-592.

[S6] Lien, C.-Y.; Hsu, Y.-F.; Liu, Y.-H.; Peng, S.-M.; Shinmyozu, T.; Yang, J.-S., Steric Engineering of Cyclometalated Pt(II) Complexes toward High-Contrast Monomer–Excimer-Based Mechanochromic and Vapochromic Luminescence. *Inorg. Chem.* **2020,** *59*, 11584-11594.
